# NiCo_2_O_4_/g-C_3_N_4_: an efficient heterogeneous photocatalyst for visible-light-driven tandem synthesis of 2,3-disubstituted quinolines

**DOI:** 10.1039/d6ra05047f

**Published:** 2026-10-08

**Authors:** Venkatesan Narasimman, Gunabalan Madhumitha

**Affiliations:** a Department of Chemistry, School of Advanced Sciences, Vellore Institute of Technology Vellore Tamil Nadu-632014 India madhumitha.g@vit.ac.in

## Abstract

The development of sustainable photocatalytic platforms for heterocyclic scaffolds remains a central challenge in synthetic chemistry. In this work, we report the synthesis of a series of nanocomposites by loading 10, 20, and 30 wt% of nickel cobaltite (NiCo_2_O_4_) into graphitic carbon nitride (g-C_3_N_4_) nanosheets. Among these nanocomposites, 20 wt% NiCo_2_O_4_-loaded g-C_3_N_4_ achieves a yield of 82% over five recyclability cycles in the synthesis of 2,3-disubstituted quinolines. The composite was engineered by anchoring spinel NiCo_2_O_4_ nanoparticles onto two-dimensional g-C_3_N_4_ nanosheets synthesized *via* a hydrothermal method. Comprehensive characterization by XRD, FT-IR spectroscopy, XPS, FE-SEM, HR-TEM, UV-vis DRS, and PL spectroscopy collectively confirmed the heterostructure formation. The objective is to evaluate the photocatalytic activity of these catalysts for synthesizing 2,3-disubstituted quinolines under 12 W blue LED light. Scavenging experiments confirm the formation of radicals under light irradiation and provide crucial mechanistic insights. The methodology demonstrates broad substrate scope, requires mild reaction conditions, and allows the use of an earth-abundant, eco-friendly catalyst, offering a sustainable alternative to conventional metal-catalysed quinoline syntheses.

## Abstract 2

A NiCo_2_O_4_/g-C_3_N_4_ heterojunction nanocomposite was prepared as a photocatalyst for synthesizing quinolines via tandem condensation-cyclization-aromatization. The optimized 20NC catalyst gave an 82% yield under mild conditions.

## Graphical abstract

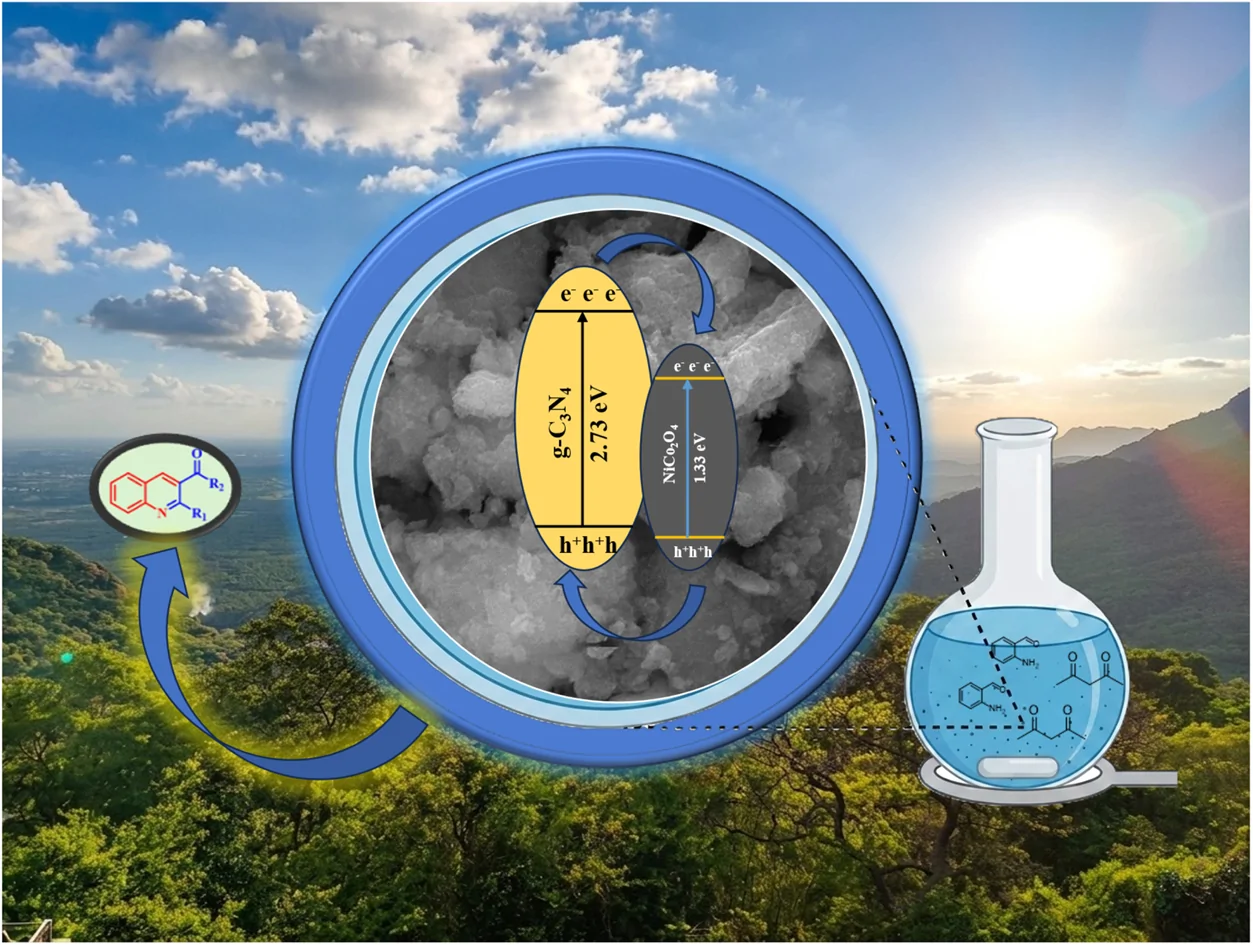

## 1 Introduction

Quinolines have unique structural scaffolds, and they occur in natural products and pharmacologically important compounds. Several quinoline derivatives exhibit excellent biological activities,^1^ including antitubercular,^2^ antifungal,^3^ anticancer,^4^ and neurodegenerative properties.^5^ Additionally, many types of quinoline derivatives have been effectively positioned as necessary drugs, such as montelukast, hydroxychloroquine, and tafenoquine.^6^ Due to their importance, various synthetic procedures, such as Friedländersynthesis, Gould–Jacob synthesis, and Skraup synthesis, have been developed. While these reactions are generally reliable, most involve the use of pre-functionalized starting materials, which are frequently unstable and unavailable for purchase.^7^ Visible light represents the most abundant form of electromagnetic radiation in our natural environment, yet most reactant molecules cannot directly absorb it to initiate chemical reactions. Visible light-mediated reactions require photocatalytic materials to act as agents that transfer energy from light to the chemical substrates.^8^ Photocatalysts fall into two broad categories: homogeneous systems, such as ruthenium and iridium complexes along with organic dyes, and heterogeneous systems, which encompass semiconductor materials, plasmonic metal nanoparticles, and emerging photoelectric substances.^9^

Semiconductor-based photocatalysis has experienced substantial growth over recent decades, offering practical solutions for both synthetic chemistry and environmental remediation. Researchers have explored numerous semiconductor materials, focusing on enhancing three key properties: light absorption capacity, charge carrier separation, and surface chemical reactivity.^10^

Modern approaches have demonstrated that engineered nanostructures and composite materials can effectively address the limitations inherent to conventional semiconductors. These strategies not only boost catalytic performance but also broaden the scope of potential applications, ranging from organic synthesis to environmental pollutant removal.^11–13^ Furthermore, investigations into heterojunction architectures and chemical doping methods have provided valuable insights for designing photocatalysts that are both highly active and operationally stable. Collectively, these advancements underscore the potential of semiconductor photocatalysis for developing efficient, environmentally friendly catalytic technologies.^14^

Due to their flexible layered structure and appropriate bandgap (2.7 eV) for visible-light absorption, g-C_3_N_4_ nanosheets have attracted a lot of attention. In addition, g-C_3_N_4_ has been employed as a major supporting matrix because of its high surface area, strong thermal stability and chemical durability, low cost, and nontoxic nature.^15^ Unfortunately, pristine and bulk g-C_3_N_4_ exhibits limited visible-light absorption and inefficient charge separation, which result in relatively low photocatalytic performance. Composites of g-C_3_N_4_ with additional semiconductor materials were created to increase the photocatalytic activity.^16^ NiCo_2_O_4_ has a spinel structure and the advantages of being inexpensive, eco-friendly, having good electrical conductivity, and showing rich redox chemistry, which qualify NiCo_2_O_4_ as a suitable component of composites with g-C_3_N_4_.^17^ NiCo_2_O_4_ has unique structural properties: Ni occupies octahedral positions, and Co occupies both octahedral and tetrahedral positions. The catalytic activity of NiCo_2_O_4_ can be improved by the formation of Co_3p_/Co_2p_ and Ni_3p_/Ni_2p_ during redox reactions. The interfacial charge transfer in the NiCo_2_O_4_/g-C_3_N_4_ heterostructure suppresses recombination and increases the lifetime of the photocarriers.^18^ These synergistic processes facilitate the activation of the carbonyl substrate and promote condensation and cyclization in the Friedländer annulation; moreover, because of this activity, NiCo_2_O_4_ was incorporated as the primary active component responsible for driving quinoline formation. As illustrated in Scheme 1, traditional approaches for the synthesis of 2,3-disubstituted quinolines have employed catalysts such as Nafion NR50,^19^ manganese oxide,^20^ and FeCl_3_,^21^ which typically require harsh acidic conditions and high temperatures, often resulting in longer reaction times and limited catalyst reusability. In contrast, the present work introduces a visible-light-driven NiCo_2_O_4_/g-C_3_N_4_ (20NC) heterojunction photocatalyst that enables the same transformation under mild conditions. This approach offers several advantages over previous methods, including high product yield, an eco-friendly aqueous-organic solvent system, and excellent catalyst reusability. Under mild, visible-light-mediated photocatalytic conditions.

**Scheme 1:**
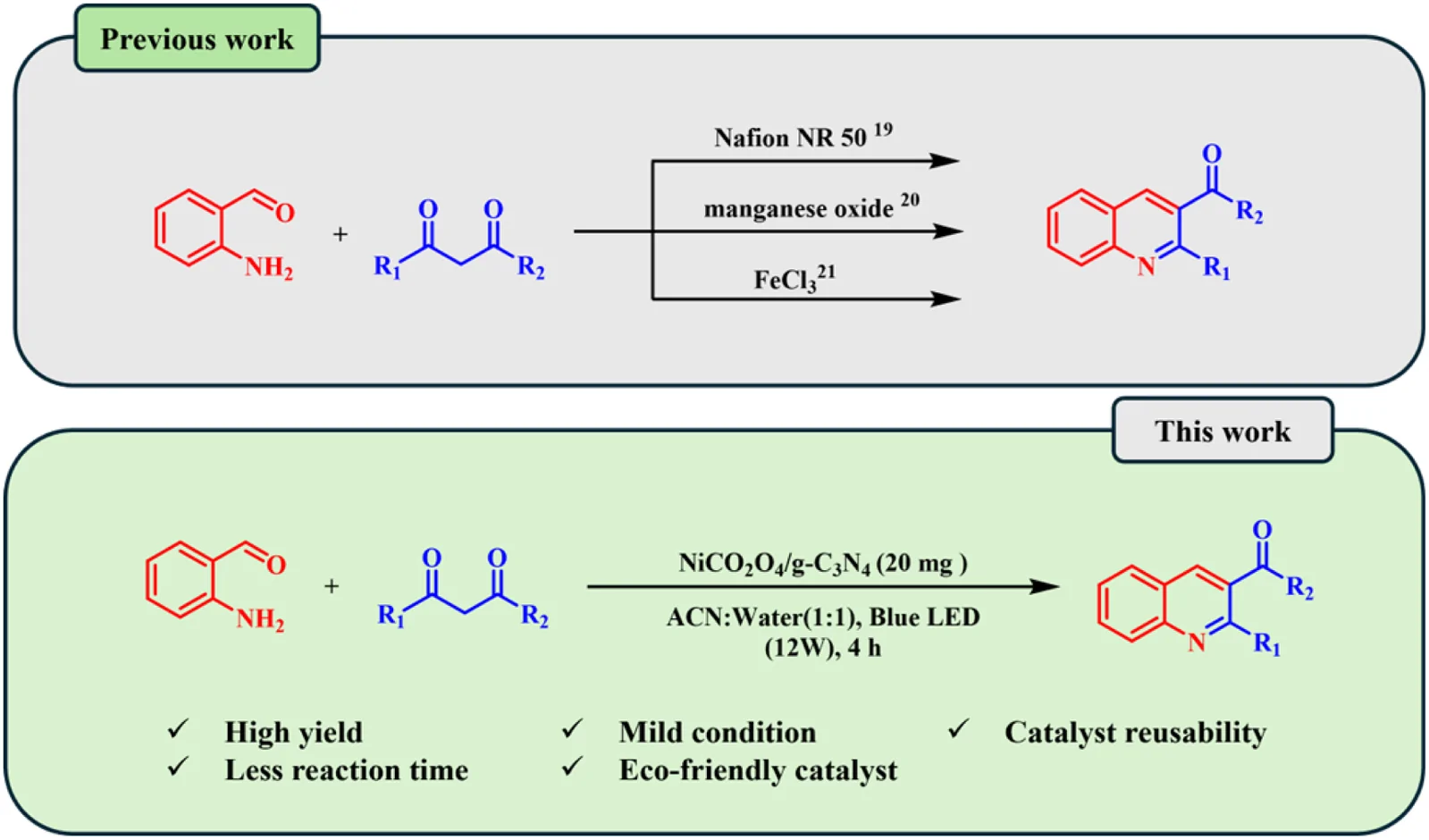
Schematic of the contrast between the present study and the existing research.

## 2 Experimental

### 2.1 Materials

For more information, please refer to the SI document (Text S1).

### 2.2 Synthesis of the g-C_3_N_4_ nanosheets

The g-C_3_N_4_ synthesis was achieved using thermal polymerization. Initially, 20 g of urea was calcined at 550 °C for 120 min in a furnace under ambient air. After the crucible naturally cooled, the resulting material was thoroughly ground into a powder. The obtained yellow powder was again calcined in a furnace at 500 °C for 180 min to obtain g-C_3_N_4_ powder.^22^

### 2.3 Synthesis of NiCo_2_O_4_

NiCo_2_O_4_ was prepared by a hydrothermal approach. Co(NO_3_)_2_·6H_2_O (2 mmol), Ni(NO_3_)_2_·6H_2_O (1 mmol) and urea (9 mmol) were dissolved in 30 mL of deionized water and stirred for 25 minutes to obtain a homogeneous solution. The mixture was transferred to a Teflon-lined stainless-steel autoclave, which was sealed and heated at 160 °C for 6 hours. After cooling to room temperature naturally, the precipitated precursor was collected, washed several times with deionized water using ultrasonic treatment to remove residual salts, and then dried. The dried material was finely ground and calcined in a furnace at 350 °C for 3 hours to yield the final NiCo_2_O_4_ powder, which was allowed to cool to room temperature before characterization.^23^

### 2.4 Synthesis of the NiCo_2_O_4_/g-C_3_N_4_ composite

The NiCo_2_O_4_/g-C_3_N_4_ composite material synthesis is shown in Fig. 1. Initially, 500 mg of g-C_3_N_4_ and 50 mg of NiCo_2_O_4_ were mixed and calcined at 300 °C for 120 min in a tube furnace. The obtained 10% NiCo_2_O_4_/g-C_3_N_4_ powder was collected and denoted as 10NC. Similarly, NiCo_2_O_4_/g-C_3_N_4_ hybrids with NiCo_2_O_4_ loadings of 10, 20, and 30% were synthesized and denoted as 10NC, 20NC, and 30NC, respectively.^22^

**Fig. 1:**
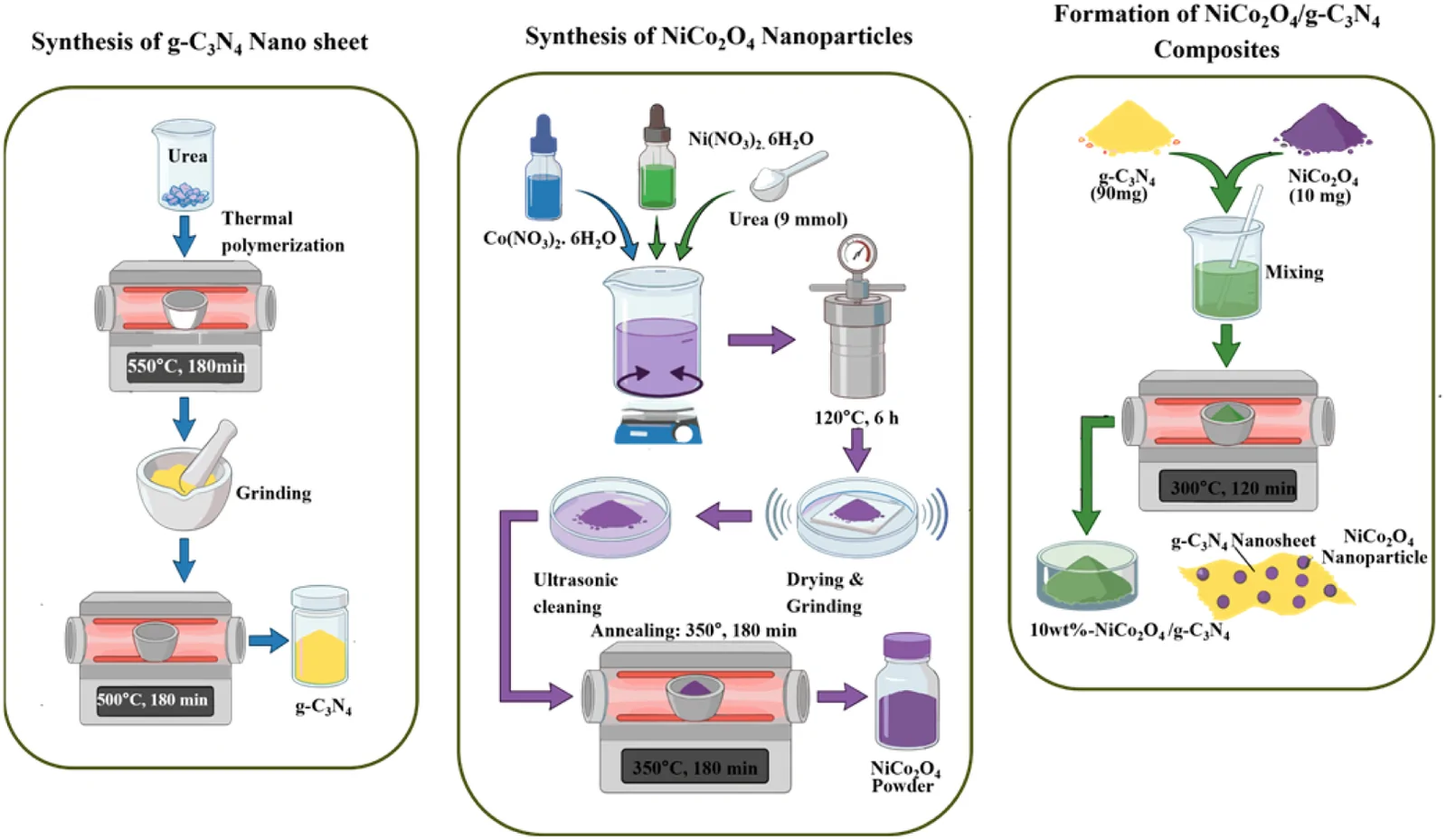
Schematic of the synthesis of NiCo_2_O_4_/g-C_3_N_4_.

### 2.5 Characterization

For more information, please refer to the SI document (Text S2).

### 2.6 Synthesis of 2,3-disubstituted quinolines

To a magnetically stirred mixture of 2-aminobenzyl aldehyde (1 mmol) and 1,3 cyclohexanediones (1.1 mmol) solubilized in acetonitrile, water (1 : 1) (3 mL) and 20 mg of 20NC photocatalyst were added, and the reaction mixture was taken in a 15 mL borosilicate glass vial and irradiated using a 12 W blue LED lamp (450–495 nm) positioned at a distance of 3 cm for a total irradiation time of 4 h at room temperature. The crude products were then purified by column chromatography, employing a petroleum ether/ethyl acetate (PET : EA) mixture in a 9 : 1 (v/v) ratio as the eluent to obtain pure compounds.

## 3 Results and discussion

### 3.1 Structural studies

The crystalline structures of NiCo_2_O_4_, g-C_3_N_4_ and NiCo_2_O_4_/g-C_3_N_4_ with different loading percentages of 10NC, 20NC, and 30NC were analysed by X-ray diffraction (XRD). According to Fig. 2a, the two diffraction peaks at 2*θ* = 27.2° and 13.3° for g-C_3_N_4_ are well matched with data in JCPDS file number 87-1526. The NiCo_2_O_4_ contained different percentages of g-C_3_N_4_. It originated from multiple tri-*s*-triazine patterns. The (100) and (002) planes of the usual graphite layer structure corresponded to these peaks.^24^ The main diffraction peaks of NiCo_2_O_4_ appeared at 2*θ* = 18.91°, 31.15°, 36.70°, 44.62°, 59.09°, and 64.98°, which signify the (111), (220), (311), (400), (511), and (440) crystal faces, respectively, according to the NiCo_2_O_4_ standard card JCPDS 20-0781. When the NiCo_2_O_4_ loading percentage increased from 10% to 30%, the characteristic diffraction peaks of NiCo_2_O_4_ became more intense. This indicates that NiCo_2_O_4_ was successfully introduced into g-C_3_N_4_*via* the annealing process. Fig. 2b shows the FT-IR spectra of the pristine and nanocomposite samples. For g-C_3_N_4_, the bands in the range of 3000 to 3500 cm^−1^ are due to the N–H stretching vibrations from residual amino groups and O–H bond vibrations of adsorbed H_2_O molecules.^25^ The peaks at 1624.0 and 1202.3 cm^−1^ were assigned to the carbon–nitrogen framework, and the peak at 800 cm^−1^ was associated with the triazine units present in g-C_3_N_4_. The FT-IR spectra of the NiCo_2_O_4_ nanoparticles show two strong metal–oxygen peaks at 648.8 and 548.4 cm^−1^, corresponding to Co–O and Ni–O, respectively. The minor amount of metal–oxygen peaks detected in the NiCo_2_O_4_/g-C_3_N_4_ composites is due to reduced NiCo_2_O_4_ content and high dispersion of NiCo_2_O_4_ on g-C_3_N_4_.^26^

**Fig. 2:**
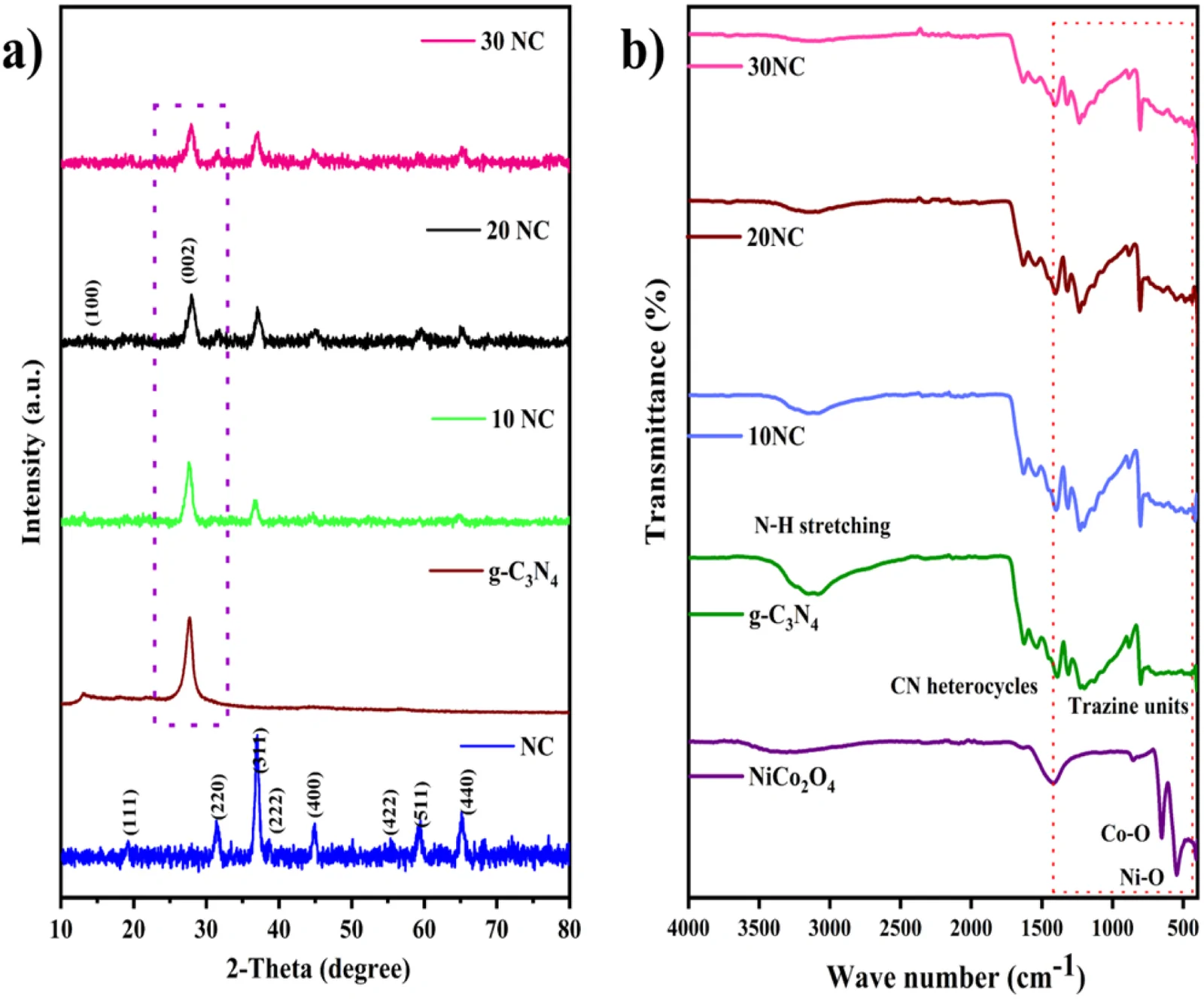
(a) XRD patterns and (b) FTIR spectra of NiCo_2_O_4_, g-C_3_N_4_, and the NiCo_2_O_4_/g-C_3_N_4_ nanocomposite.

XPS was used to determine the chemical composition of the NiCo_2_O_4_/g-C_3_N_4_ composites. Signals for Ni, Co and O originate from the spinel oxide, whereas C and N arise from the g-C_3_N_4_ nanosheets (Fig. 3a). The Co_2p_ region (Fig. 3b) displays two main species plus their associated satellite features. Peaks located at 777.5 and 792.5 eV are assigned to Co^2+^, while those appearing at 790.3 and 794.4 eV are attributed to Co^3+^. Deconvolution of the Ni 2p spectrum (Fig. 3c) reveals two spin–orbit doublets: the components at 857.7 and 875.7 eV correspond to Ni^2+^, and those at 855.7 and 873.3 eV to Ni^3+^. Two nickel shake-up satellites are observed near 862.1 and 880.4 eV. The O 1s envelope (Fig. 3d) can be fitted with three contributions: the low-energy peak at 529.4 eV is assigned to lattice metal–oxygen bonds, the component at 530.8 eV to adsorbed water or loosely bound oxygen species, and the higher-energy feature at 532.6 eV to surface hydroxyl groups. In the C 1s spectrum (Fig. 3e), peaks at 285.3, 286.8 and 288.8 eV are consistent with graphitic carbon (C–C), carbon bound to nitrogen (C–N), and sp^2^-hybridized carbon in the g-C_3_N_4_ network, respectively. The N 1s spectrum Fig. 3f is resolved into three peaks at 396.9, 398.7 and 401.2 eV, which are assigned to sp^2^-hybridized C=N–C, tertiary (sp^3^) nitrogen (N–(C)^3^) and C–N–H (amino-type nitrogen), respectively. Together, these XPS features confirm the coexistence of mixed-valence Ni and Co in the oxide phase, and the 20NC composite binding energies show slight shifts compared to pristine NiCo_2_O_4_ and g-C_3_N_4_, respectively. These shifts indicate strong electronic interactions at the interface and is consistent with interfacial charge distribution between NiCo_2_O_4_ and g-C_3_N_4_.

**Fig. 3:**
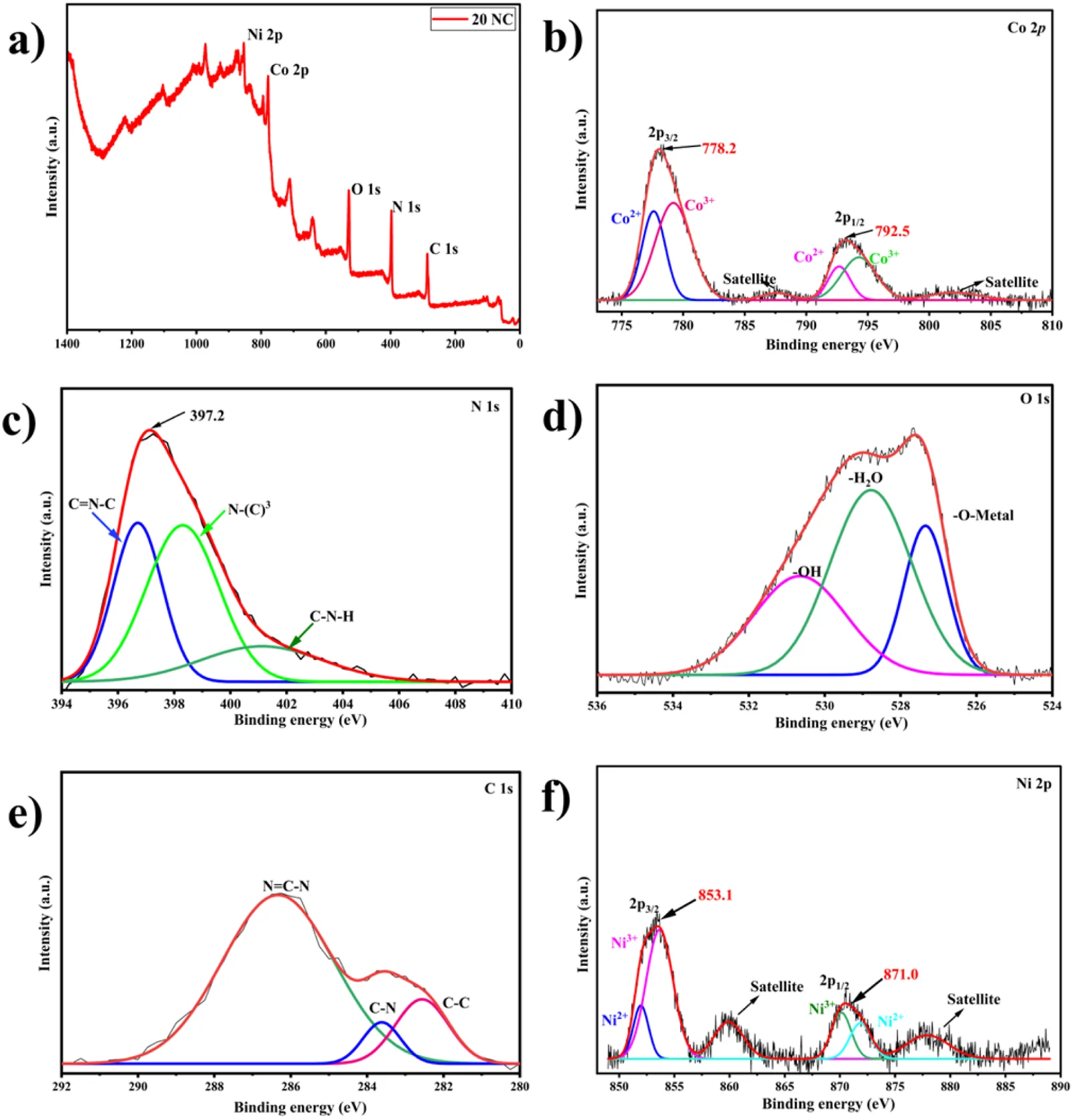
(a) Survey spectrum of 20NC, and deconvoluted spectra of (b) cobalt 2p, (c) nickel 2p, (d) oxygen 1s, (e) carbon 1s, and (f) nitrogen 1s.

### 3.2 Morphological studies

FESEM was used to examine the morphology of the pristine NiCo_2_O_4_ and composite 20NC catalyst. Pristine NiCo_2_O_4_ shows a well-defined urchin-like structure with uniformly dispersed features. These structures provide a larger and more effective heterojunction interface (Fig. 4a and b). The NiCo_2_O_4_-anchored g-C_3_N_4_ composite (20NC) exhibits an irregular, layered flake-like structure (Fig. 4c). NiCo_2_O_4_ particles are observed decorating the surface of the g-C_3_N_4_ nanosheets, consistent with the formation of a NiCo_2_O_4_/g-C_3_N_4_ heterostructure. The EDS spectrum in Fig. 4d shows the uniform distribution of the elements, and Fig. 4e confirms the presence of the following elements with weight percentages Co (5.4%), Ni (2.9%), C (31.8%), N (46.0%), O (14.0%). Moreover, elemental mapping (Fig. 4f–j) shows those elements are distributed across the sample, indicating good spatial correlation between the oxide particles and the carbon nitride matrix. Together, the morphological and compositional data support successful anchoring of NiCo_2_O_4_ onto the g-C_3_N_4_ nanosheets and the creation of an intimate interfacial contact that can facilitate charge transfer in photocatalytic applications.

**Fig. 4:**
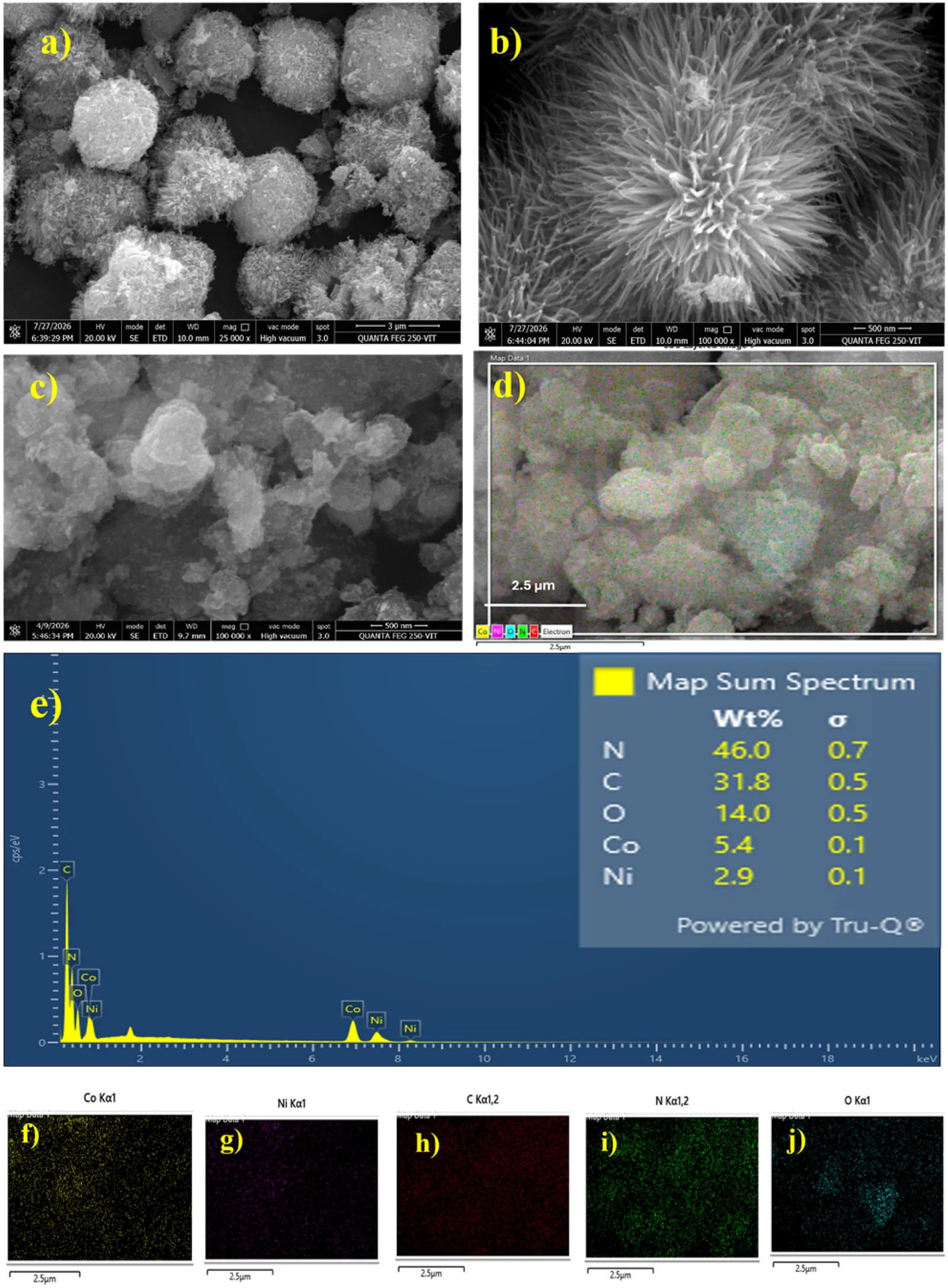
(a and b) FESEM micrograph of pristine NiCo_2_O_4_, (c) FESEM micrograph of the 20NC composite, (d) scanned area of 20NC, (e) 20NC nanocomposite EDS spectrum, and (f) elemental mapping of cobalt (Co), (g) nickel (Ni), (h) carbon (C), (i) nitrogen (N), and (j) oxygen (O).

HR-TEM micrograph analysis was also performed to analyse the morphological characteristics and heterojunction formation. Fig. 5a and 4b illustrate the strong contact between NiCo_2_O_4_ particles and the g-C_3_N_4_ nanosheets. Fig. 5c illustrates the construction of a heterojunction between the g-C_3_N_4_ sheets and NiCo_2_O_4_ nanoparticles. The lattice fringes corresponding to NiCo_2_O_4_ (0.46 nm) indicate the existence of the (111) plane, which is seen to be directly anchored on the g-C_3_N_4_ surface. This close interfacial contact without any gap confirms the formation of a heterojunction rather than a simple physical mixture, and provides the necessary structural pathway for interfacial charge transfer, as shown in (Fig. 5d). The nanocomposite SAED pattern is depicted in (Fig. 5e); this pattern reveals that the nanocomposite possesses crystalline characteristics. The particle size histogram (Fig. 5f), obtained from around 50 particles measured across the TEM micrographs, shows a narrow, near-Gaussian size distribution ranging from 6 to 20 nm, with most particles concentrated between 10 and 14 nm. The average particle size was determined to be 12 nm, indicating that a relatively uniform particle distribution formed during synthesis.

**Fig. 5:**
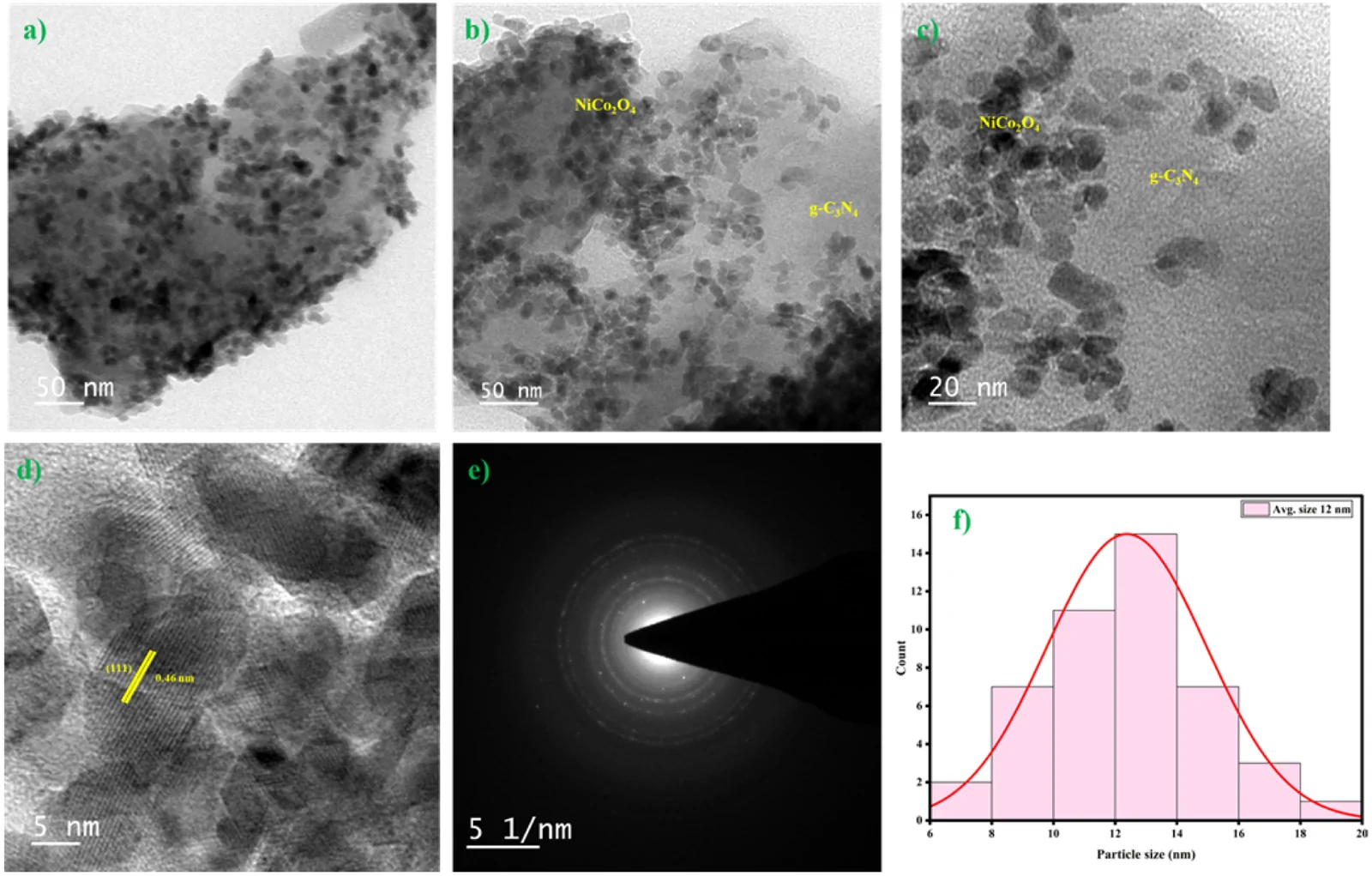
(a and b) TEM micrographs, (c) heterojunction of 20NC, (d) *d*-spacing of NiCo_2_O_4_, (e) SAED pattern, and (f) average particle size.

### 3.3 Optical studies

UV-vis diffuse reflectance spectroscopy (DRS) was used to compare the optical properties of pristine NiCo_2_O_4_, g-C_3_N_4_, and composites containing 10, 20 and 30 wt% NiCo_2_O_4_ (10NC, 20NC, 30NC). As shown in Fig. 6a, g-C_3_N_4_ exhibits an absorption edge near 450 nm, consistent with its limited visible-light response. In contrast, the 20NC composite shows excellent absorption at 380–455 nm, originating from g-C_3_N_4_, superimposed on the broad background absorption of NiCo_2_O_4_ that extends throughout the visible region. This strong absorption in the blue-light region overlaps well with the emission of the 12 W blue LED (450–460 nm), supporting its selection as the optimal irradiation source for this photocatalytic system. Band-gap energies were estimated from Tauc plots (Fig. 6b). The derived *E*_g_ values are 1.33 eV for NiCo_2_O_4_, 2.73 eV for g-C_3_N_4_, and 1.95, 2.19, and 1.87 eV for 10NC, 20NC, and 30NC, respectively. These results indicate that incorporating NiCo_2_O_4_ narrows the effective optical band gap of the composites and enhances their visible-light absorption compared with bare g-C_3_N_4_.

**Fig. 6:**
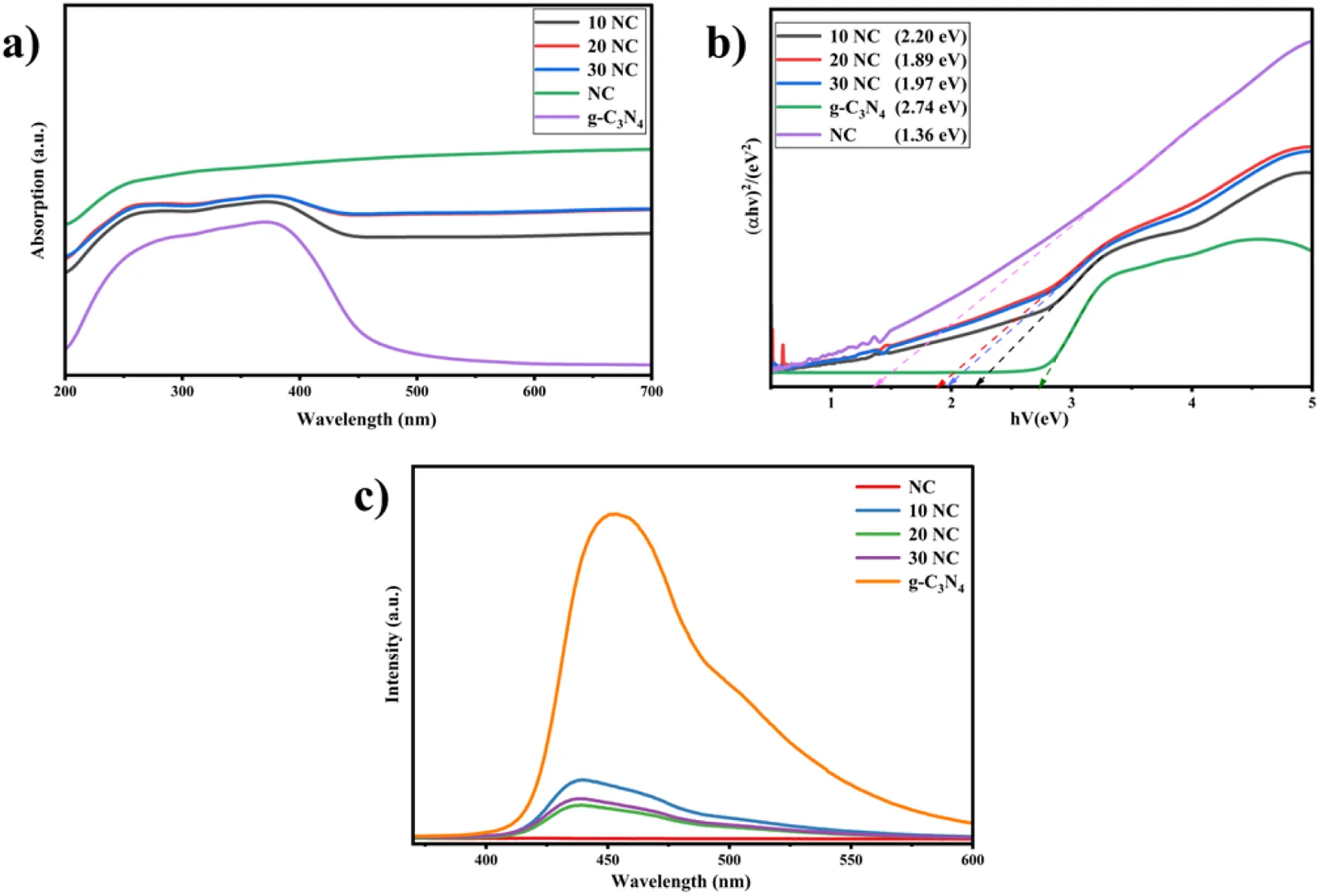
(a) UV-vis spectra, (b) band gap spectra, and (c) PL spectra of the NiCo_2_O_4_/g-C_3_N_4_ nanocomposite.

Photoluminescence (PL) spectroscopy was used to investigate the electron–hole recombination rate; a high intensity was associated with a high recombination rate and low photocatalytic efficiency. As shown in Fig. 6c, the emission peaks were obtained at 475 nm. g-C_3_N_4_ shows a highly intense fluorescence emission, whereas NiCo_2_O_4_ showed no PL due to rapid non-radiative relaxation pathways. After loading NiCo_2_O_4_, the emission intensity decreased. This quenching is indicative of efficient charge transfer across the NiCo_2_O_4_/g-C_3_N_4_ interface. The excited electrons from g-C_3_N_4_ are transferred to NiCo_2_O_4_, which confirms heterojunction-driven charge dynamics rather than physical coexistence.^27^

### 3.4 Photocatalytic application of the NiCo_2_O_4_/g-C_3_N_4_ nanocomposite

The reaction was conducted using 2-aminobenzaldehyde (1 mmol) (1) and 1,3-cyclohexadione (1.1 mmol) (2), catalyzed by the 20NC nanocomposite under optimized conditions under blue light irradiation to obtain the product 3a (Table 1). The reaction was optimized by adjusting several parameters, including catalyst, light and solvent. Initially, the reaction was optimized by employing several different photocatalysts, including NiO, Co_3_O_4_, and g-C_3_N_4_, which gave moderate yields of approximately 36–55% (Table 4). The nanocomposite 20NC exhibited a notable yield of 82% within 4 h (Table 4, entry 7). The reaction was optimized using several solvent systems, including water (H_2_O), ethanol (EtOH), methanol (MeOH), dimethyl formamide (DMF), tetrahydrofuran (THF), and acetonitrile (ACN). The combination of acetonitrile and water (1 : 1) showed a higher yield than the other solvents (Table 2, entry 8). Moreover, the light source was optimized by conducting the experiment in the dark and under various colours of light. In the dark, a yield of less than 10% was obtained due to the mild intrinsic Lewis acid catalytic.

**Table 1:** Substrate scope of 2,3-disubstituted quinolines^a^

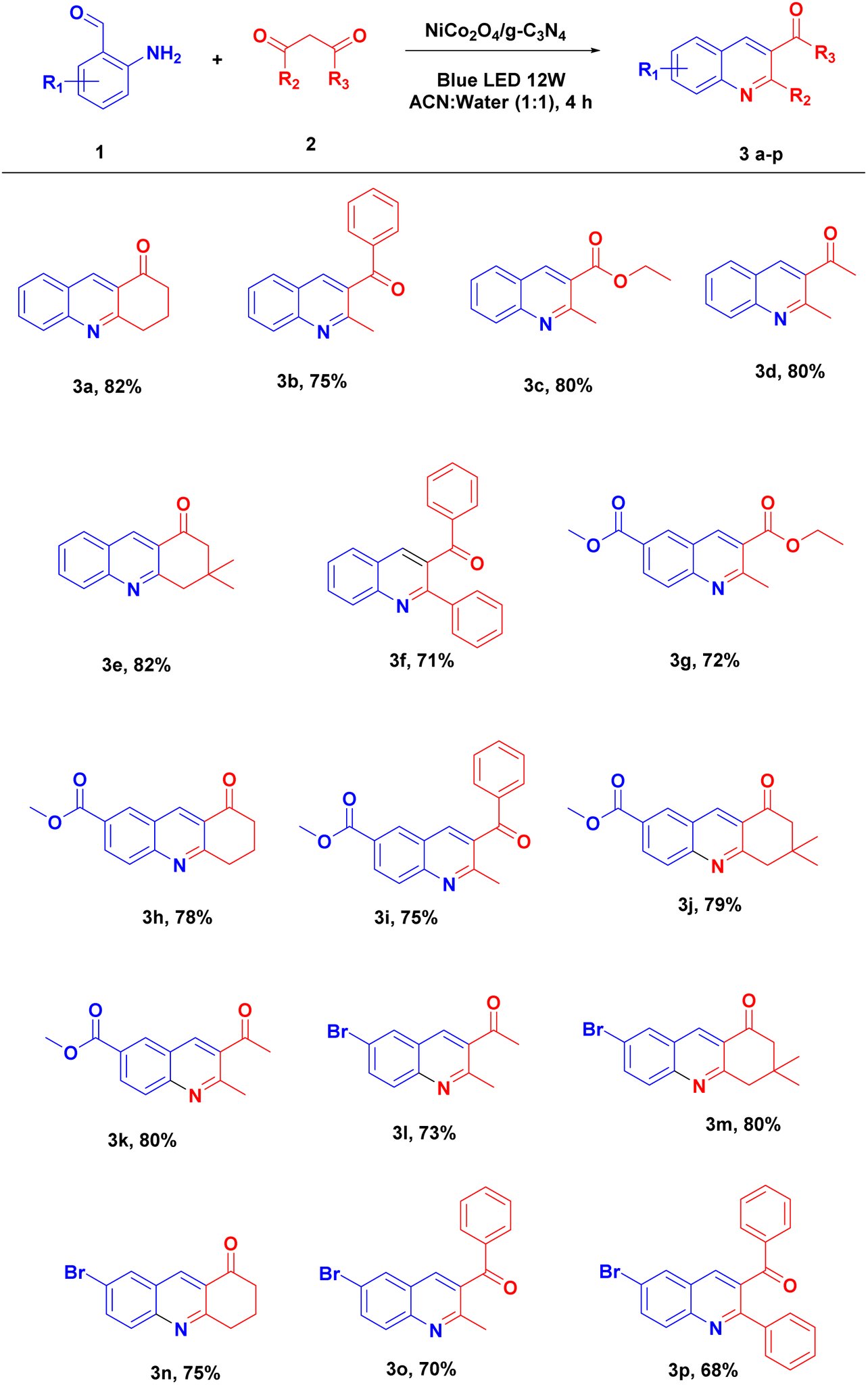

a Reaction conditions: 2-aminobenzaldehyde (1 mmol), 1,3-cyclohexadione (1.1 mmol), catalyst (20 mg), solvent (3 mL), 12 W blue LED, reaction time-4 h, NR-no reaction; isolated yields are given.

**Table 2:** Solvent optimization^a^

Entry	Solvent	Catalyst	Yield (%)
1	Water	20NC	40
2	EtOH	20NC	32
3	MeOH	20NC	28
4	THF	20NC	47
5	ACN	20NC	75
7	DMF	20NC	68
8	ACN : water (1 : 1)	20NC	82
9	DMSO	20NC	62
10	1,3-Dioxane	20NC	60
11	Toluene	20NC	65

a Reaction conditions: 2-aminobenzaldehyde (1 mmol), 1,3-cyclohexadione (1.1 mmol), catalyst (20 mg), solvent (3 mL), 12 W blue LED, reaction time-4 h, NR-no reaction; isolated yields are given.

Activity of the NiCo_2_O_4_ surface, which facilitates slow thermal condensation between the reactants. A substantially higher yield was obtained under 12 W blue light irradiation, which confirms that visible-light-induced charge carrier generation is the primary driving force for efficient product formation (Table 3). Various percentages of the NiCo_2_O_4_-doped catalysts were used to test the reaction, including 10NC, 20NC, and 30NC. Among all these catalysts, 20NC showed the highest yield (82%). The products were analysed using ^1^H and ^13^C NMR and GC-MS.

**Table 3:** Optimization with various light sources^a^

Entry	Catalyst	Light source	Yield (%)
1	20NC	Dark	10
2	20NC	Visible light (300 W)	70
3	20NC	Green LED	72
4	20NC	Blue LED	82

a Reaction conditions: 2-aminobenzaldehyde (1 mmol), 1,3-cyclohexadione (1.1 mmol), catalyst (20 mg), solvent (3 mL), 12 W blue LED, reaction time-4 h, NR-no reaction; isolated yields are given.

**Table 4:** Optimization of the photocatalyst

Entry	Catalyst	Light	Source	Time (h)	Yield^a^ (%)
1	Without catalyst	Blue LED	0	8	Trace
2	NiO	Blue LED	20	7	32
3	Co_3_O_4_	Blue LED	20	8	35
4	NiCo_2_O_4_	Blue LED	20	7	58
5	g-C_3_N_4_	Blue LED	20	8	35
6	20NC	Blue LED	10	4.5	74
7	20NC	Blue LED	30	4	82
8	20NC	Blue LED	30	4	80

a Reaction conditions: 2-aminobenzaldehyde (1 mmol), 1,3-cyclohexadione (1.1 mmol), catalyst (20 mg), solvent (3 mL), 12 W blue LED, NR-no reaction; isolated yields are given.

### 3.5 Mechanistic pathway

The photocatalytic mechanism starts with light excitation, which generates electron–hole pairs (h+/e−), as shown in Fig. 7. The holes oxidize the β-dicarbonyl compound (a) into a radical intermediate (b). This radical reacts with 2-aminobenzaldehyde to form the intermediate moiety (c), which subsequently undergoes radical-mediated transformation to form (d). Dehydration of this intermediate leads to the formation of the imine-like structure (e), followed by an intramolecular nucleophilic attack by the amino group on the carbonyl carbon to close the ring. A final dehydration step (f) restores aromaticity to obtain the substituted quinoline derivative (g). This dual-catalyst system enhances efficiency by promoting charge separation, allowing the reaction to proceed under mild conditions.^28^

**Fig. 7:**
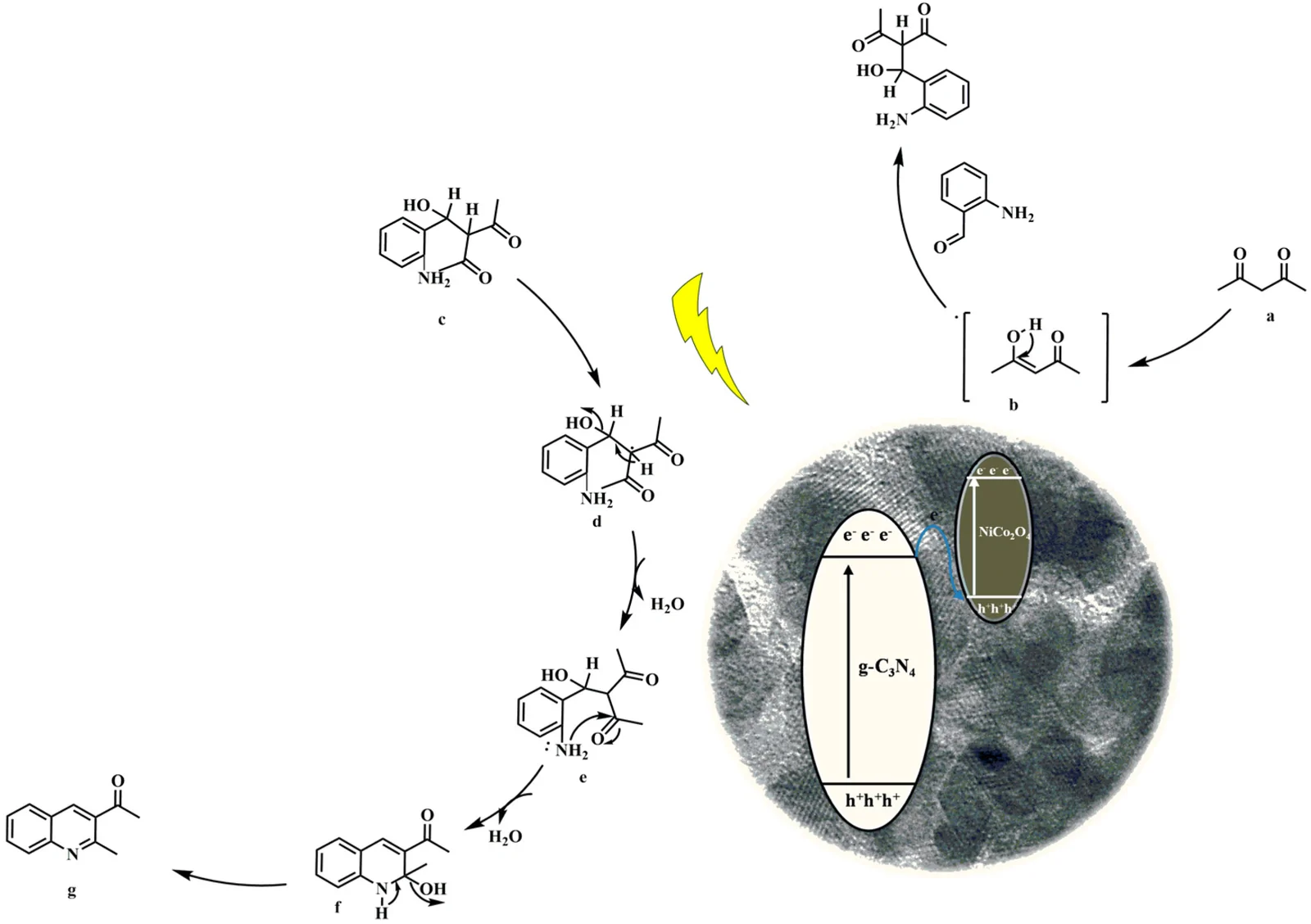
Proposed mechanism for the formation of 2,3-disubstituted quinolines.

### 3.6 Trapping study

Radical-trapping experiments prove the photocatalytic mechanism. AgNO_3_, triethanolamine (TEOA), and 2,2,6,6-tetramethylpiperidine-1-oxyl (TEMPO) were employed to quench electrons, holes, and radical intermediates, respectively. Addition of AgNO_3_ or TEOA suppressed product formation, as monitored by TLC monitoring, indicating the involvement of both photogenerated electrons and holes. TEMPO trapped the radical derived from substrate 3b to give a TEMPO-3b adduct, which was confirmed by high-resolution mass spectrometry (Fig. S52), with only trace amounts of product detected.

### 3.7 Reusability study

Reusability tests evaluated the stability of 20NC. The model reaction was repeated for five consecutive cycles using the recovered catalyst (Fig. 8b). After the fifth run, the product yield fell to 75%, indicating a small loss of activity. The used sample was analysed by XRD and compared with fresh 20NC (Fig. 8a). The main diffraction peaks of NiCo_2_O_4_ and g-C_3_N_4_ remain present after cycling, with only minor changes in peak intensity and slight broadening, suggesting preserved crystal phases.

**Fig. 8:**
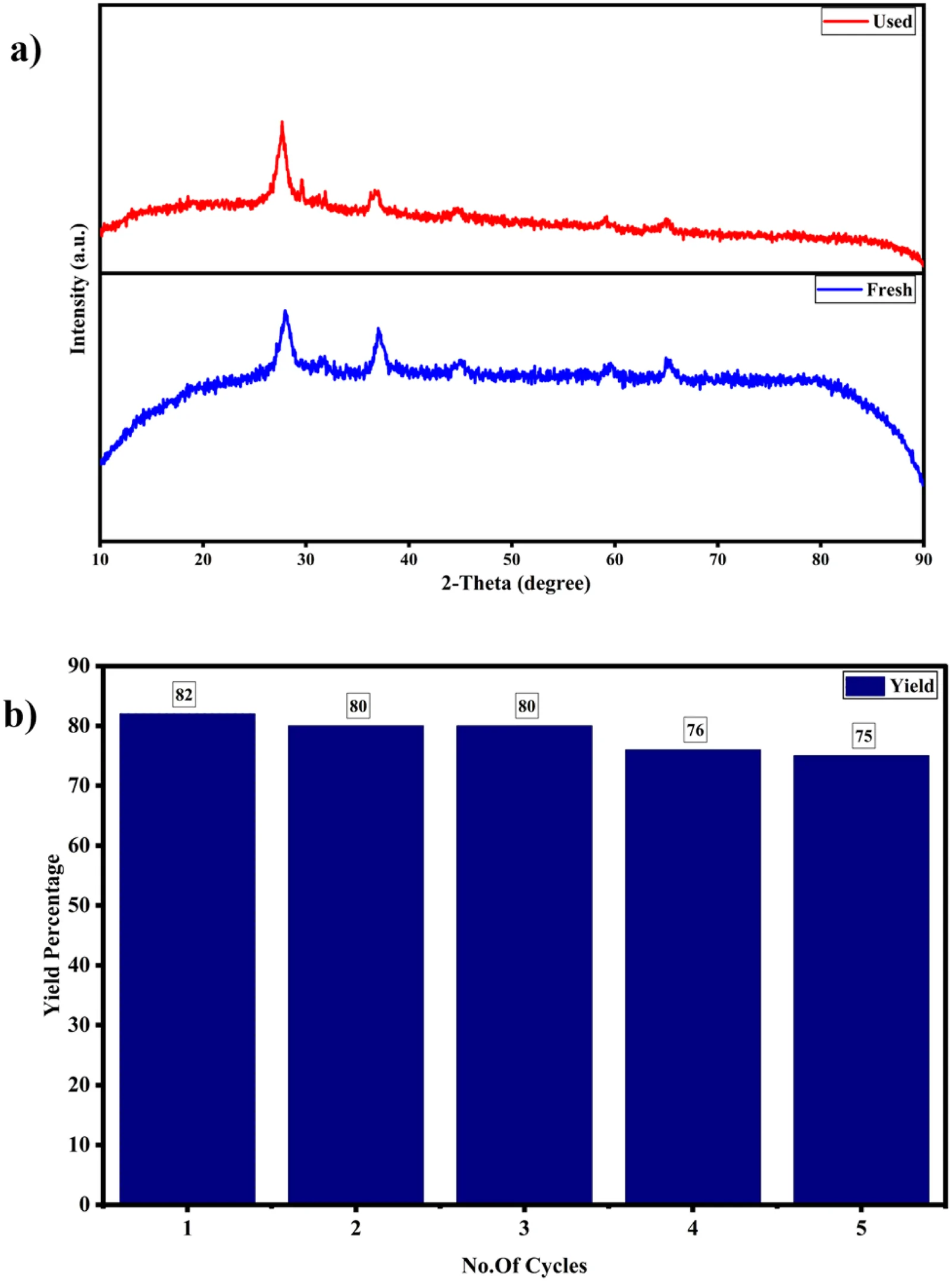
(a) XRD spectra of fresh and used 20NC. (b) Reusability of 20NC.

## 4 Conclusion

The NiCo_2_O_4_/g-C_3_N_4_ heterojunction nanocomposite was successfully fabricated and demonstrated to be an efficient visible-light photocatalyst for the synthesis of 2,3-disubstituted quinoline derivatives. The optimized 20NC nanocomposite delivered a yield of 82% under 12 W blue LED irradiation within 4 hours. The reaction proceeded through a visible-light-driven condensation followed by a cyclization and aromatization cascade under mild conditions without requiring noble metals or harsh reagents. The earth-abundant nature, broad substrate scope, and operational simplicity of this photocatalytic system make a sustainable and practical platform for the construction of pharmacologically important nitrogen heterocycles.

## Author contributions

Venkatesan Narasimman: writing—original draft, data curation, formal analysis, investigation. Gunabalan Madhumitha: writing—review & editing, supervision, conceptualization, methodology.

## Conflicts of interest

The authors declare that they have no conflicts of interest.

## Supplementary Material

RA-OLF-D6RA05047F-s001

## Data Availability

The data supporting the findings of this study are available within the article and its supplementary information (SI). Supplementary information is available. See DOI: https://doi.org/10.1039/d6ra05047f.
