# Supplementary material for: NiCo2O4/g-C3N4: an efficient heterogeneous photocatalyst for visible-light-driven tandem synthesis of 2,3-disubstituted quinolines
Source: RSC Adv. 2026 Oct 8;16(46):60975–82. doi: 10.1039/d6ra05047f (PMC13647312; doi:10.1039/d6ra05047f)
Supplement: Supplementary file 1 [file RA-016-D6RA05047F-s001.pdf]

Supplementary Information (SI) for RSC Advances.
This journal is © The Royal Society of Chemistry

NiCo₂O₄/g-C₃N₄: An Efficient Heterogeneous Photocatalyst for Visible-Light-Driven Tandem Oxidative Synthesis of 2,3-Disubstituted Quinolines

Venkatesan Narasimman, Gunabalan Madhumitha*

*Department of Chemistry, School of Advanced Sciences, Vellore Institute of Technology, Vellore, Tamil Nadu-632014.

Corresponding author: madhumitha.g@vit.ac.in

Supporting Information

Table of Contents

1. General information-- S1

2. Characterization Details--S2

3. Spectral and analytical characterization ---S3

4. ^{1}H, ^{13}C NMR and Mass Spectra of Products (3a-p) --S4

5. Radical scavenging study---S52

S1. Chemicals used:

Cobalt nitrate hexahydrate from Thermo Fisher Scientific. Nickel(II) nitrate hexahydrate from TCI chemicals, 2-amino benzaldehyde purchased from Chemscene Pharma, and substituted Di-ketones were purchased from Avra Synthesis Pvt. Ltd. All reagents were of analytical grade and used without further purification.

S2. General characterization details:

The Bruker D8 Advance X-ray diffraction technique using Cu Kα radiation (λ = 0.154 nm) in the range of 2 theta from 10 to 90° at a scan speed of 2° min^{-1} was used to acquire X-ray diffraction patterns for all samples. JASCO-Model name V-670 was used to record UV-DRS. The surface chemical composition was confirmed using X-ray photoelectron spectra (XPS) (TPHI 5000 Versa Probe IV) with an Al Kα X-ray source. Field emission scanning electron microscopy (Quanta FEG 250) was used to assess the morphology, EDAX analysis and elemental mapping of the prepared nanomaterials. Transmission electron microscopy (Tecnai G2-20 dual field emission microscope) was used to examine the morphology of the prepared samples. ^{1}H and ^{13}C NMR spectra were recorded on a BRUKER (400 MHz) spectrometer. Tetramethyl silane (TMS) is used as an internal standard. Perkin Elmer Clarus 680&600 model was used to analyze mass of the compounds through GC-MS.

S3. Spectral and analytical characterization of the 1-(2-methylquinolin-3-yl)ethan-1-one and 3,4-dihydroacridin-1(2H)-one derivatives (3a-p)

3a	3,4-dihydroacridin-1(2H)-one	
Molecular Formula<br>Molecular Weight (g/mol)<br>Colour	$C_{13}H_{11}NO$<br>197.0841<br>White solid	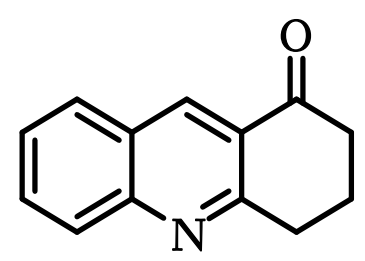
^{1}H-NMR (400 MHz, $CDCl_3$, Me_4Si) (ppm): ▱	8.85 (s, 1H), 7.06-7.04 (d, 1H), 7.95-7.92 (d, 1H) 7.83- 7.79 (m, 1H), 7.57-7.53 (m, 1H), 3.34-3.311 (m, 2H), 2.82-2.79 (m, 2H), 2.31-2.25 (m, 2H).	
^{13}C-NMR (100 MHz, $CDCl_3$, Me_4Si) (ppm) : ▱	197.96, 161.97, 149.68, 137.13, 132.36, 129.74, 128.58, 126.83, 126.70, 126.33, 39.09, 33.46, 21.78	
Mass(ESI)m/z: (M+)	Calculated<br>197.2370	Found<br>197.3243
3b	(2-methylquinolin-3-yl) (phenyl)methanone	
Molecular Formula<br>Molecular Weight (g/mol)<br>Colour	$C_{17}H_{13}NO$<br>247.0997<br>White solid	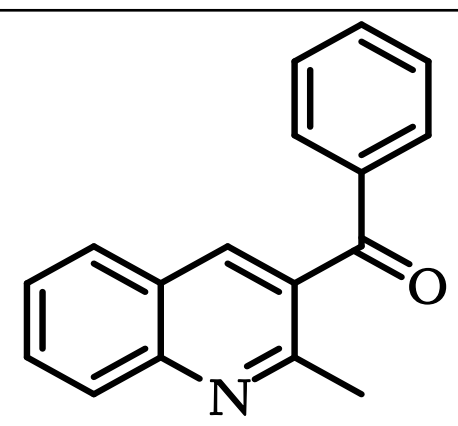
^{1}H-NMR (400 MHz, $CDCl_3$, Me_4Si) (ppm): ▱	8.05-8.01 (m, 2H), 7.79-7.69 (m, 4H), 7.59-7.41 (m, 4H), 2.67 (s, 3H).	
^{13}C-NMR (100 MHz, $CDCl_3$, Me_4Si) (ppm) : ▱	196.71, 156.70, 147.90, 137.26, 136.93, 133.76, 132.26, 131.18, 130.19, 128.78, 128.56, 128.12, 126.7, 125.32, 24.16	
Mass(ESI)m/z: (M+)	Calculated<br>247.2970	Found<br>247.4592

3c	ethyl 2-methylquinoline-3-carboxylate	
Molecular Formula<br>Molecular Weight (g/mol)<br>Colour	$C_{13}H_{13}NO_2$<br>215.0946<br>White solid	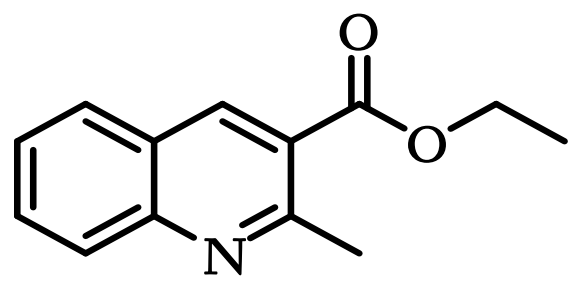
^{1}H-NMR (400 MHz, $CDCl_3$, Me_4Si) (ppm): □	8.66 (s, 1H), 7.98-7.96 (d, 1H), 7.80-7.68 (m, 2H) 7.48- 7.19 (m, 1H), 4.38-4.36 (m, 2H), 2.92 (s, 3H), 1.40-1.36 (m, 3H).	
^{13}C-NMR (100 MHz, $CDCl_3$, Me_4Si) (ppm) : □	166.52, 158.47, 148.51, 139.96, 131.71, 128.46, 126.55, 123.97, 61.42, 25.63, 14.33	
Mass(ESI)m/z: (M+)	Calculated<br>215.2520	Found<br>215.4044

3d	1-(2-methylquinolin-3-yl) ethan-1-one	
Molecular Formula<br>Molecular Weight (g/mol)<br>Colour	$C_{12}H_{11}NO$<br>185.0841<br>White solid	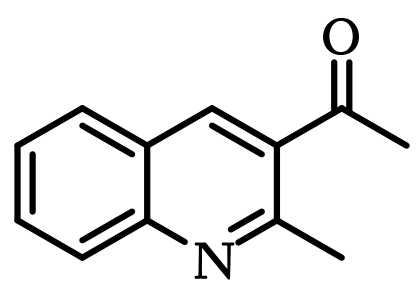
^{1}H-NMR (400 MHz, $CDCl_3$, Me_4Si) (ppm): □	8.43 (s, 1H), 8.02-8.00 (d, 1H), 7.81-7.79 (d, 1H) 7.75- 7.71 (m, 1H), 7.52-7.48 (m, 1H), 2.86 (s, 3H), 2.65 (s, 3H).	
^{13}C-NMR (100 MHz, $CDCl_3$, Me_4Si) (ppm) : □	199.90, 157.61, 148.19, 138.26, 131.77, 131.11, 128.52, 128.35, 126.71, 125.62, 29.23, 25.61	
Mass(ESI)m/z: (M+)	Calculated<br>185.2260	Found<br>185.3624

3f	phenyl(2-phenylquinolin-3-yl)methanone	
**Molecular Formula**<br>**Molecular Weight (g/mol)**<br>**Colour**	$C_{22}H_{15}NO$<br>309.3680<br>White solid	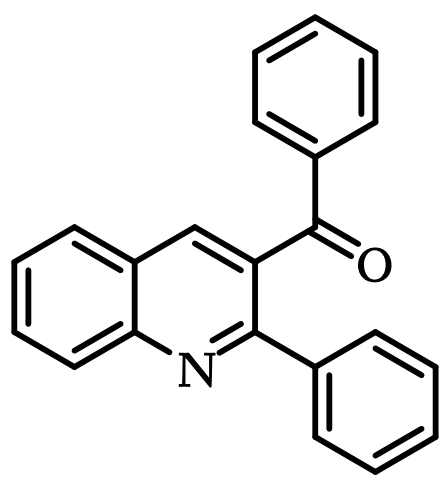
**^{1}H-NMR (400 MHz, $CDCl_3$, Me_4Si) (ppm): δ**	8.27 (s, 1H), 8.18-8.16 (d, 1H), 7.84-7.76 (m, 2H), 7.65- 7.63 (m, 2H), 7.56-7.53 (m, 3H), 7.42-7.38 (m, 1H), 7.27-7.18 (m, 5H).	
**^{13}C-NMR (100 MHz, $CDCl_3$, Me_4Si) (ppm) : δ**	196.98, 157.48, 148.36, 139.71, 137.64, 137.04, 131.21, 129.99, 129.68, 129.28, 128.86, 128.42, 128.40, 127.32, 125.81.	
**Mass(ESI)m/z: (M+)**	Calculated<br>309.3680	Found<br>309.2813

3g	3-ethyl 6-methyl 2-methylquinoline-3,6-dicarboxylate	
Molecular Formula<br><br>Molecular Weight (g/mol)<br><br>Colour	$C_{15}H_{15}NO_4$<br><br>273.1004<br><br>White solid	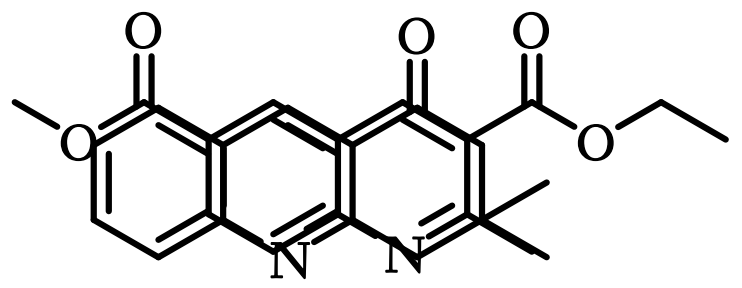
^{1}H-NMR (400 MHz, $CDCl_3$, Me_4Si) (ppm): ▱	8.76 (s, 1H), 7.99-7.97 (d, 1H), 7.88-7.86 (d, 1H), 7.75-7.71 (m, 1H), 7.57-7.46 (m, 1H), 3.12 (s, 2H), 2.58 (s, 2H), 1.38 (s, 6H)	
^{13}C-NMR (100 MHz, $CDCl_3$, Me_4Si) (ppm) : ▱	165.26, 164.87, 149.88, 146.65, 142.26, 139.57, 138.20, 126.67, 125.20, 123.87, 123.81, 32.78, 28.38, 24.72, 13.27	
Mass(ESI)m/z: (M+)	Calculated<br><br>273.1001	Found<br><br>273.3263

3h	methyl 8-oxo-5,6,7,8-tetrahydroacridine-2-carboxylate	
Molecular Formula<br><br>Molecular Weight (g/mol)<br><br>Colour	$C_{15}H_{13}NO_3$<br><br>255.0895<br><br>White solid	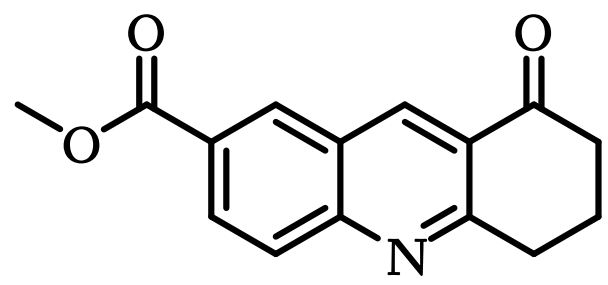
^{1}H-NMR (400 MHz, $CDCl_3$, Me_4Si) (ppm): ▱	8.94 (s, 1H), 8.65 (s, 1H), 8.36-8.34 (d, 1H), 8.16-8.14 (m, 1H), 3.94 (s, 3H), 3.36-3.34 (m, 2H), 2.78-2.75 (m, 2H), 2.26-2.23 (m, 2H)	
^{13}C-NMR (100 MHz, $CDCl_3$, Me_4Si) (ppm) : ▱	197.21, 166.17, 164.24, 138.55, 132.73, 131.84, 128.74, 128.46, 127.00, 126.05, 52.58, 38.96, 33.44, 21.50.	
Mass(ESI)m/z: (M+)	Calculated<br><br>255.0895	Found<br><br>255.3860

3i	methyl 3-benzoyl-2-methylquinoline-6-carboxylate	
Molecular Formula<br>Molecular Weight (g/mol)<br>Colour	$C_{19}H_{15}NO_3$<br>305.1052<br>White solid	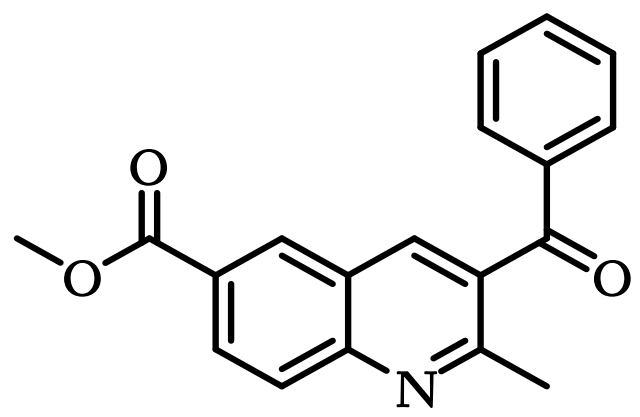
^{1}H-NMR (400 MHz, $CDCl_3$, Me_4Si) (ppm): □	8.50 (s, 1H), 8.31-8.29 (d, 1H), 8.15 (s, 1H) 8.10-8.09 (d, 1H), 7.78-7.76 (d, 2H), 7.61-7.57 (m, 1H), 7.46-7.42 (m, 2H), 3.91 (s, 3H), 2.71 (s, 3H)	
^{13}C-NMR (100 MHz, $CDCl_3$, Me_4Si) (ppm) : □	196.08, 166.32, 159.29, 137.92, 136.91, 134.01, 133.02, 131.16, 130.73, 130.18, 128.88, 128.40, 124.56, 52.52, 24.31	
Mass(ESI)m/z: (M+)	Calculated<br>305.1052	Found<br>305.3864

3k	methyl 8-oxo-5,6,7,8-tetrahydroacridine-2-carboxylate	
Molecular Formula<br>Molecular Weight (g/mol)<br>Colour	$C_{14}H_{13}NO_3$<br>243.0895<br>White solid	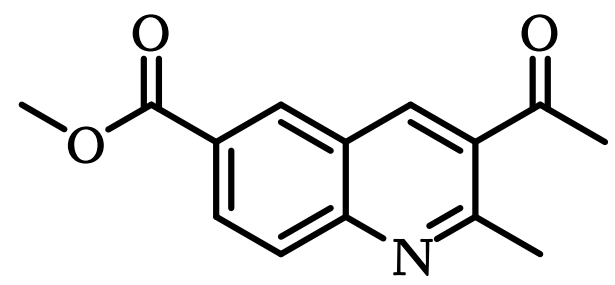
^{1}H-NMR (400 MHz, $CDCl_3$, Me_4Si) (ppm): □	8.55-8.50 (m, 2H), 8.29 (bs, 1H), 8.00 (bs, 1H), 3.93 (s, 3H), 2.86 (s, 3H), 2.66 (s, 3H).	
^{13}C-NMR (100 MHz, $CDCl_3$, Me_4Si) (ppm) : □	99.57, 166.31, 160.18, 149.97, 139.10, 133.45, 131.45, 128.90, 128.29, 124.87, 52.58, 29.30, 25.82.	
Mass(ESI)m/z: (M+)	Calculated<br>243.0895	Found<br>243.3567

3j	methyl 6,6-dimethyl-8-oxo-5,6,7,8-tetrahydroacridine-2-carboxylate	
Molecular Formula<br>Molecular Weight (g/mol)<br>Colour	$C_{17}H_{17}NO_3$<br>283.1208<br>White solid	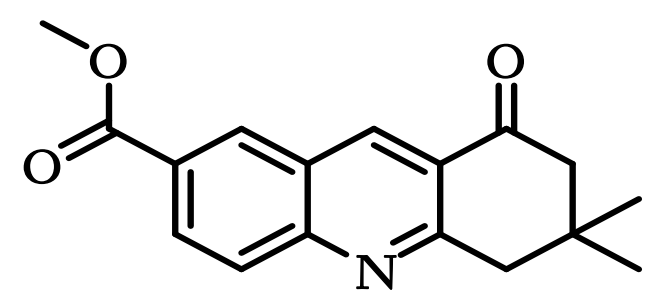
^{1}H-NMR (400 MHz, $CDCl_3$, Me_4Si) (ppm): □	8.94 (s, 1H), 8.62-8.61 (d, 1H), 8.32-8.29 (m, 1H), 8.02-8.00-(m, 1H), 3.94 (s, 3H), 3.15 (s, 2H), 2.60 (s, 2H), 1.09 (s, 6H).	
^{13}C-NMR (100 MHz, $CDCl_3$, Me_4Si) (ppm) : □	197.21, 166.17, 164.24, 138.55, 132.73, 131.84, 128.74, 128.46, 127.00, 126.05, 52.58, 38.96, 33.44, 21.50.	
Mass(ESI)m/z: (M+)	Calculated<br>283.1208	Found<br>283.2705

3m	7-bromo-3,3-dimethyl-3,4-dihydroacridin-1(2H)-one	
Molecular Formula<br>Molecular Weight (g/mol)<br>Colour	$C_{17}H_{17}NO_3$<br>283.1208<br>White solid	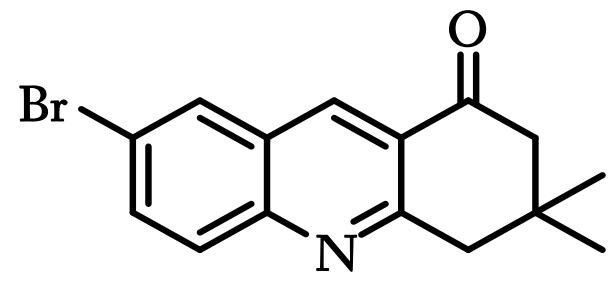
^{1}H-NMR (400 MHz, $CDCl_3$, Me_4Si) (ppm): ▱	8.65 (s, 1H), 8.01-8.00 (d, 1H), 7.86-7.76 (m, 2H), 3.10 (s, 2H), 2.59 (s, 2H), 1.08 (s, 6H).	
^{13}C-NMR (100 MHz, $CDCl_3$, Me_4Si) (ppm) : ▱	197.58, 161.26, 148.54, 135.51, 135.38, 131.45, 130.33, 127.91, 125.85, 120.47, 52.44, 47.16, 32.74, 28.39.	
Mass(ESI)m/z: (M+)	Calculated<br>303.0259	Found<br>303.1607

3l	1-(6-bromo-2-methylquinolin-3-yl) ethan-1-one	
Molecular Formula<br>Molecular Weight (g/mol)<br>Colour	$C_{12}H_{10}BrNO$<br>262.9946<br>White solid	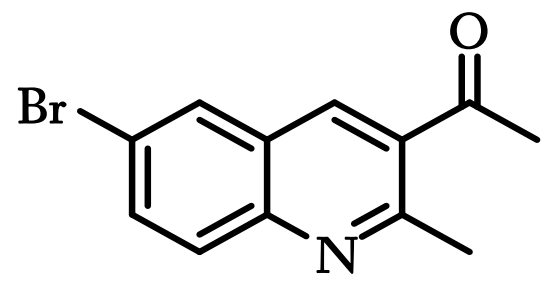
^{1}H-NMR (400 MHz, $CDCl_3$, Me_4Si) (ppm): ▱	8.27 (bs, 1H), 7.92-75 (m, 3H), 2.79 (s, 3H), 2.62 (s, 3H)	
^{13}C-NMR (100 MHz, $CDCl_3$, Me_4Si) (ppm) : ▱	199.70, 158.02, 146.75, 136.93, 135.00, 131.91, 130.33, 130.24, 126.78, 120.40, 29.38, 25.59	
Mass(ESI)m/z: (M+)	Calculated<br>262.9946	Found<br>263.2434

3n	7-bromo-3,4-dihydroacridin-1(2H)-one	
Molecular Formula<br>Molecular Weight (g/mol)<br>Colour	$C_{13}H_{10}BrNO$<br>274.9946<br>White solid	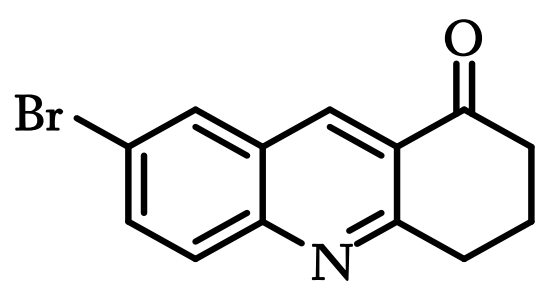
^{1}H-NMR (400 MHz, $CDCl_3$, Me_4Si) (ppm): □	8.74 (s, 1H), 8.08 (s, 1H), 7.93-7.90 (m, 1H), 7.87-7.84 (m, 1H), 3.31-3.28 (m, 2H), 2.82-2.79 (m, 2H), 2.31-2.25 (m, 2H)	
^{13}C-NMR (100 MHz, $CDCl_3$, Me_4Si) (ppm) : □	197.54, 162.38, 148.17, 135.98, 135.66, 131.42, 130.30, 127.94, 126.86, 120.47, 39.02, 33.38, 21.60	
Mass(ESI)m/z: (M+)	Calculated<br>274.9946	Found<br>275.2734

3o	(6-bromo-2-methylquinolin-3-yl)(phenyl)methanone)	
Molecular Formula<br>Molecular Weight (g/mol)<br>Colour	$C_{17}H_{12}BrNO$<br>325.0102<br>White solid	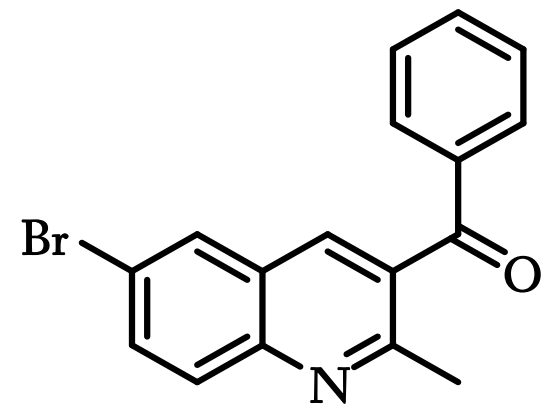
^{1}H-NMR (400 MHz, $CDCl_3$, Me_4Si) (ppm): δ	7.95-7.88 (m, 3H), 7.78-7.74 (m, 3H), 7.57-7.56 (m, 1H), 7.45-7.41 (m, 2H), 2.65 (s, 3H).	
^{13}C-NMR (100 MHz, $CDCl_3$, Me_4Si) (ppm) : δ	196.29, 157.15, 146.53, 136.94, 135.47, 133.97, 133.07, 130.38, 130.16, 130.01, 128.85, 126.48, 120.49, 24.17.	
Mass(ESI)m/z: (M+)	Calculated<br>325.0102	Found<br>325.2795
3p	(6-bromo-2-phenylquinolin-3-yl)(phenyl)methanone)	
Molecular Formula<br>Molecular Weight (g/mol)<br>Colour	$C_{22}H_{14}BrNO$<br>387.0259<br>White solid	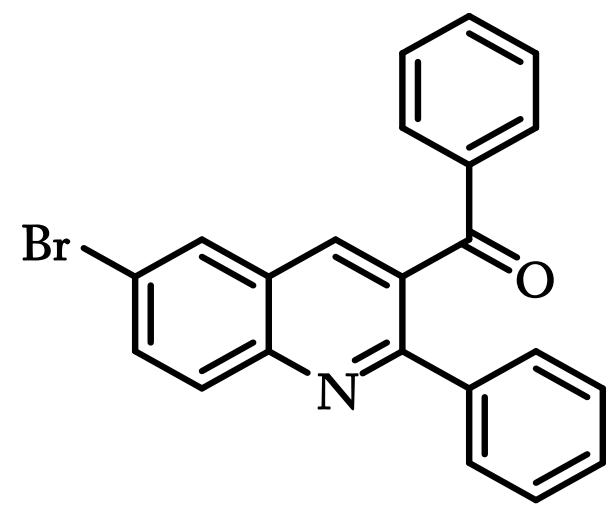
^{1}H-NMR (400 MHz, $CDCl_3$, Me_4Si) (ppm): δ	8.16 (s, 1H), 8.05-7.99 (m, 2H), 7.83-7.81 (m, 1H), 7.64-7.61 (m, 2H), 7.55-7.52 (m, 2H), 7.47-7.40 (m, 2H), 7.29-7.19 (m, 3H).	
^{13}C-NMR (100 MHz, $CDCl_3$, Me_4Si) (ppm) : δ	196.50, 157.78, 148.88, 139.27, 137.66, 136.73, 136.43, 135.64, 133.56, 133.47, 132.47, 131.02, 130.30, 129.97, 129.22, 128.78, 128.73, 128.49, 127.18, 126.31, 121.20,	

**Mass(ESI)m/z: (M+)**	Calculated<br><br>387.0259	Found<br><br>387.3423

S1. ^{1}H NMR, ^{13}C NMR and GC-MS Spectra of Products

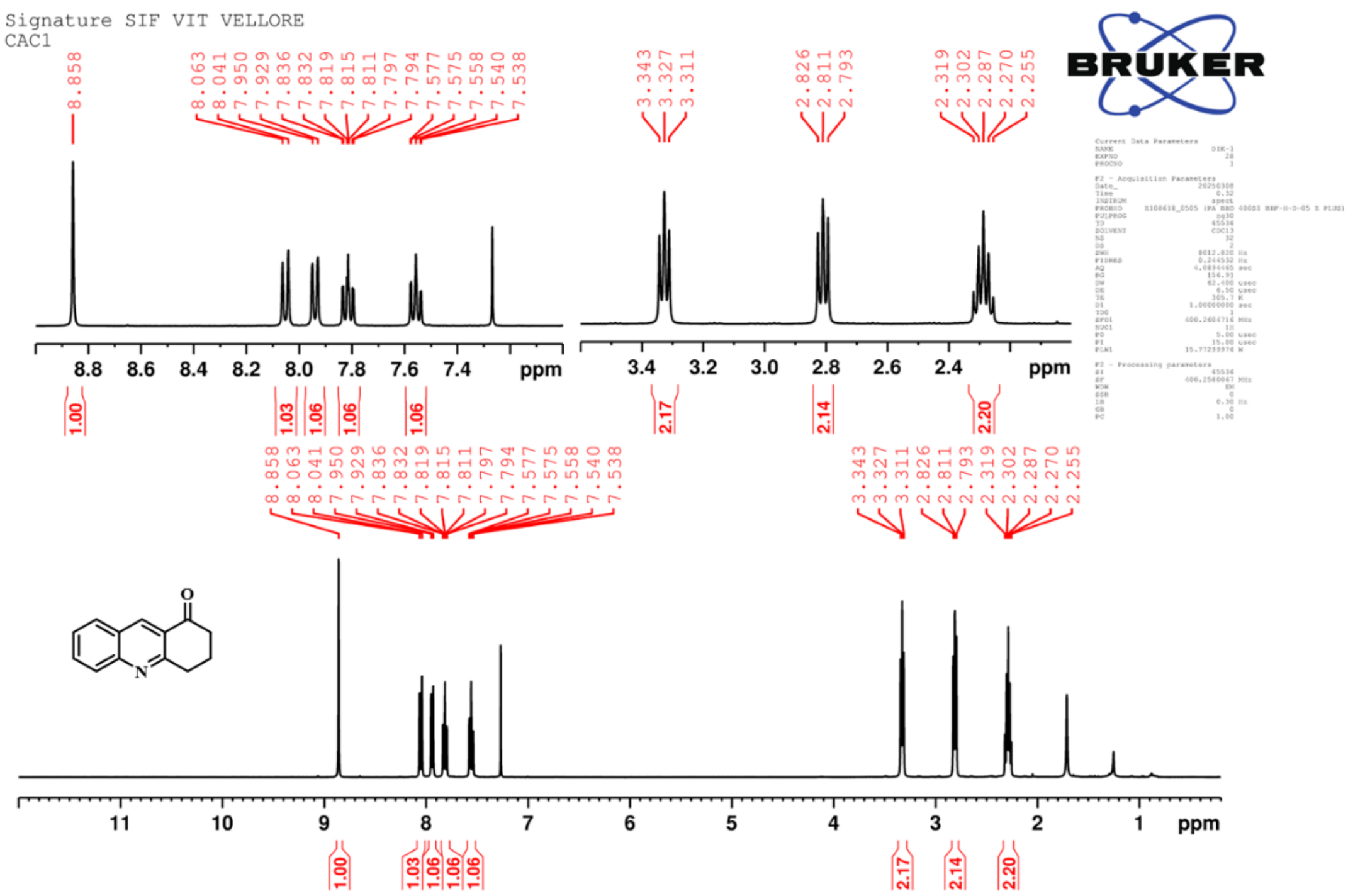

Figure S4. ^{1}H NMR spectrum of compound 3a

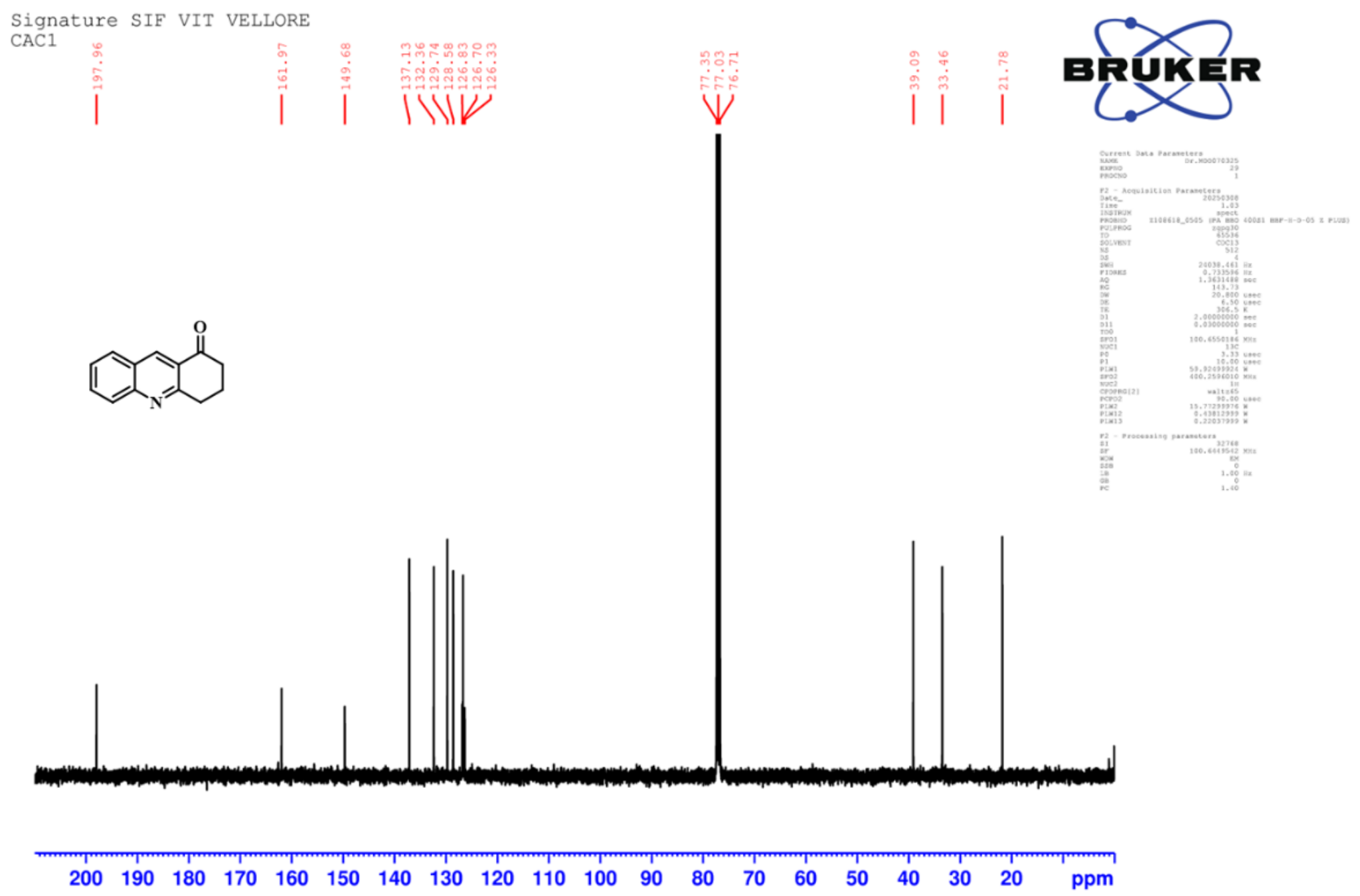

Figure S5. ¹³C NMR spectrum of compound 3a

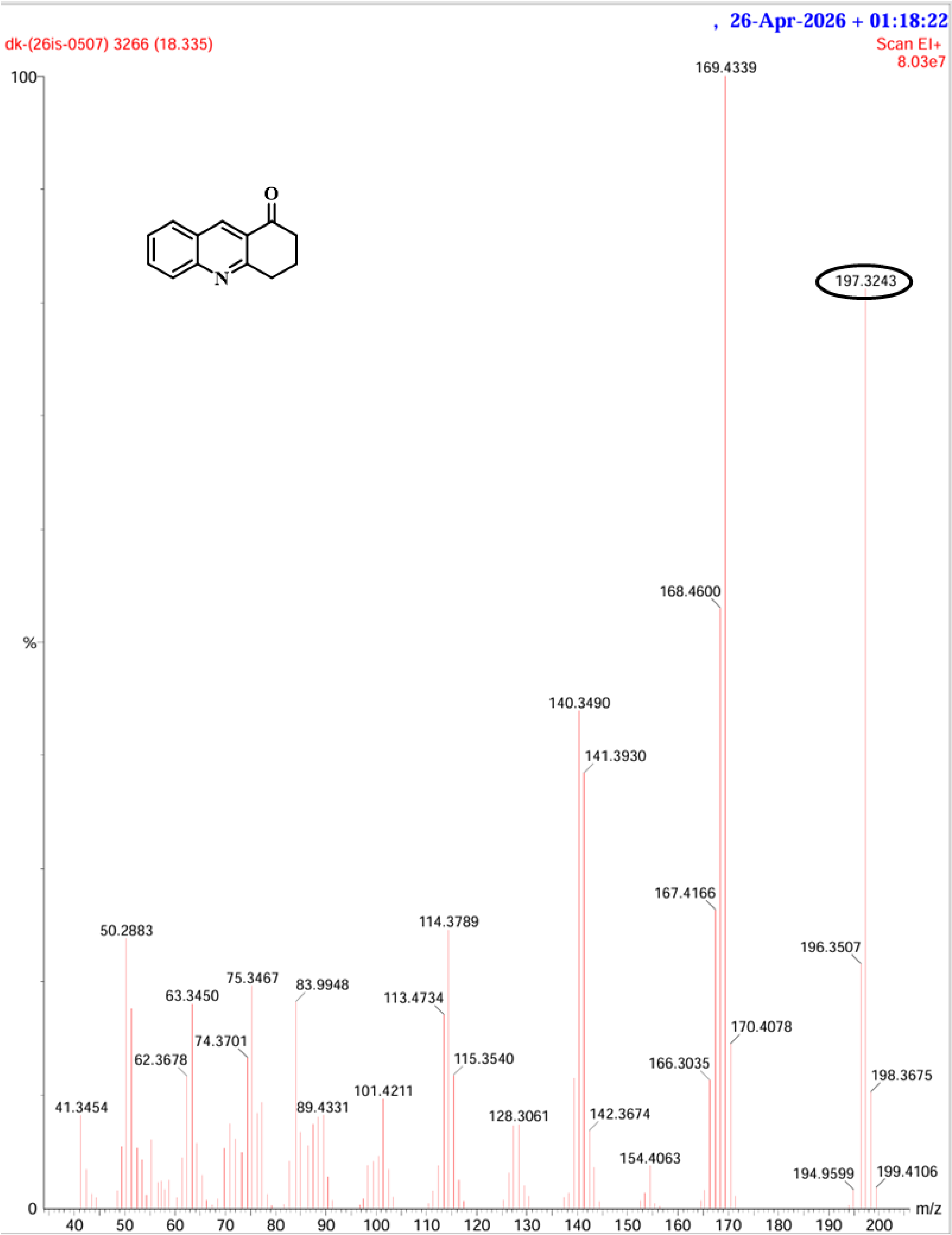

Figure S6. GC-MS spectrum of 3a

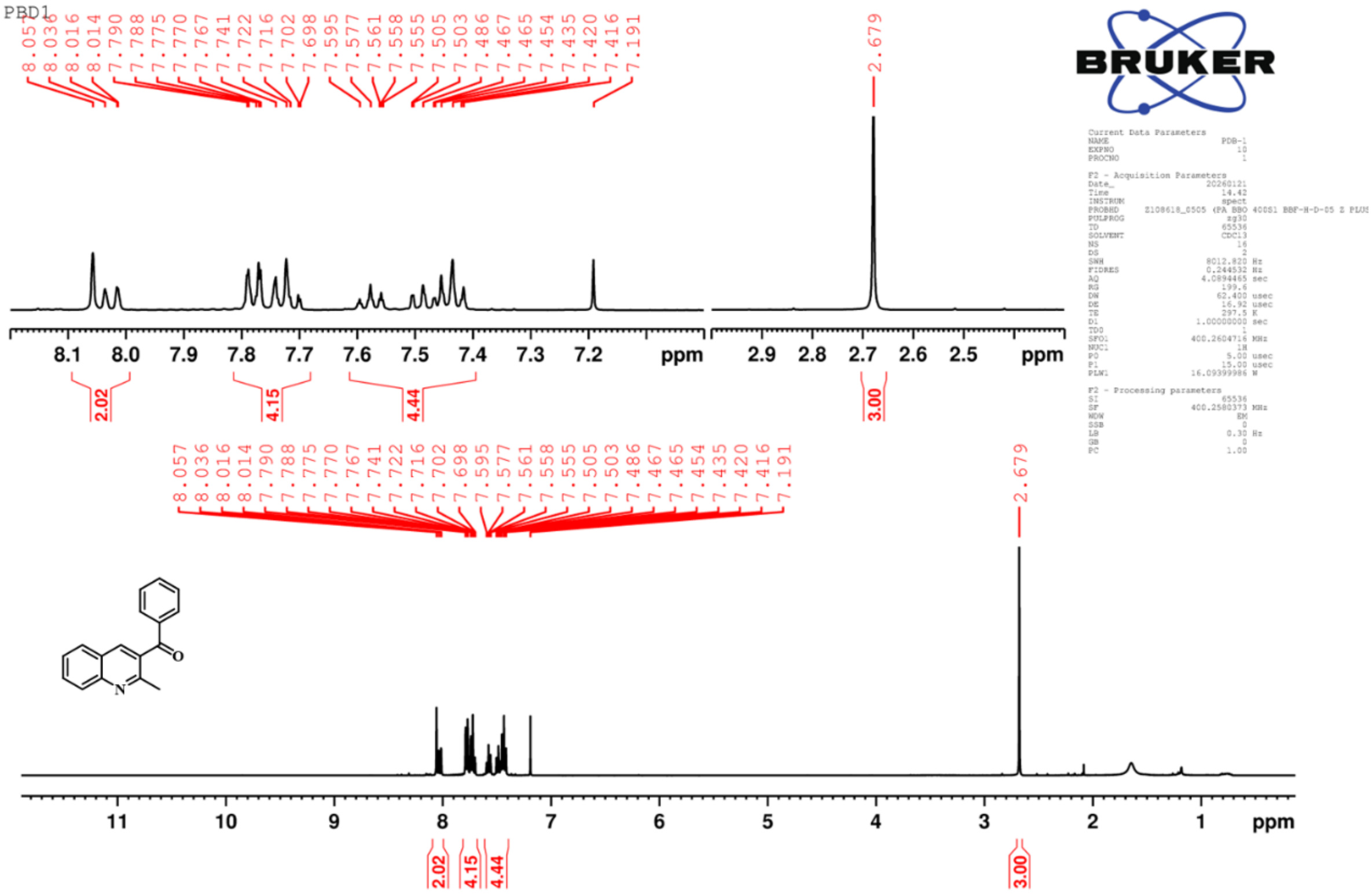

Figure S7. ^{1}H NMR spectrum of compound 3b

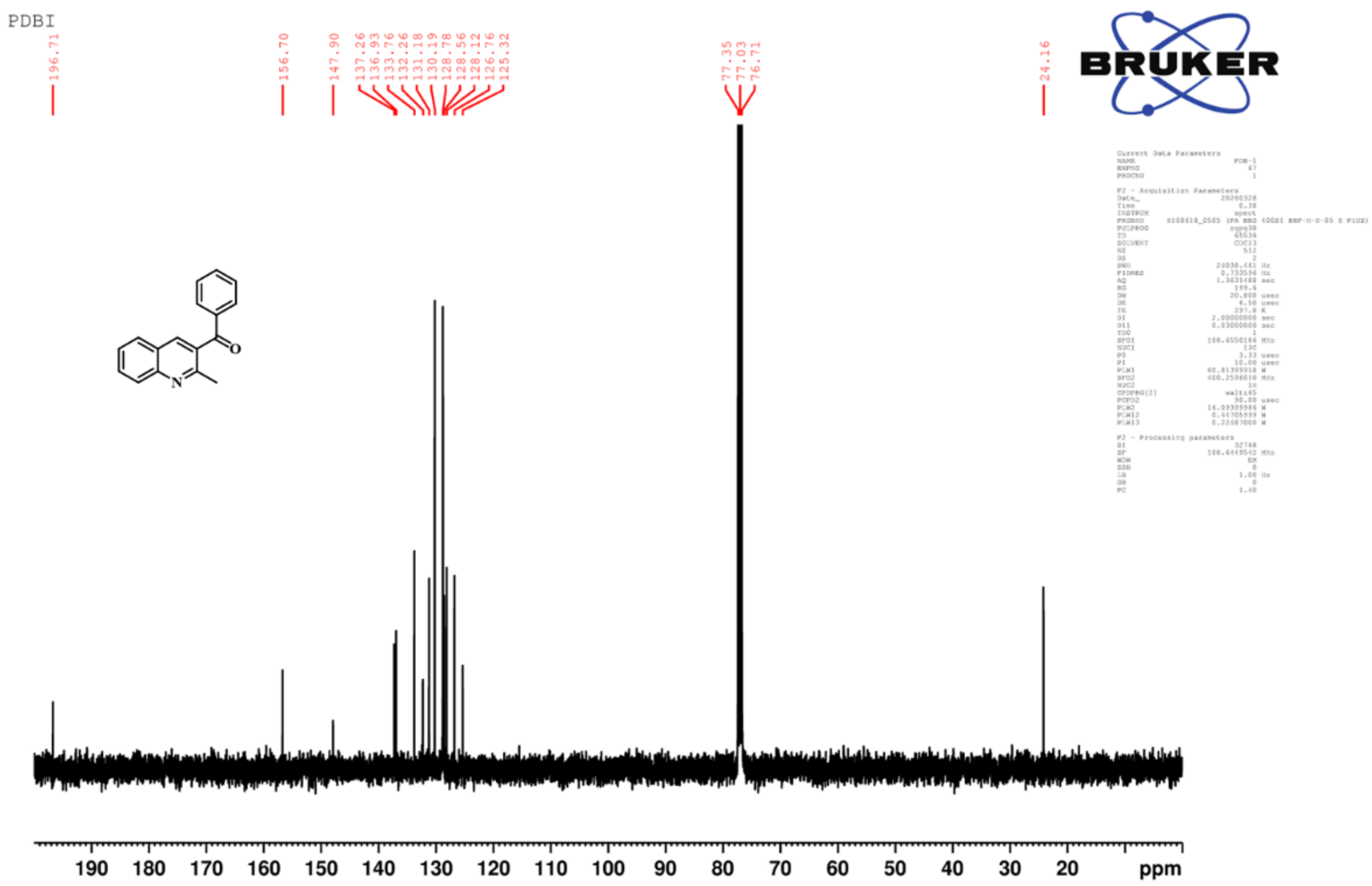

Figure S8. ^{13}C NMR spectrum of compound 3b

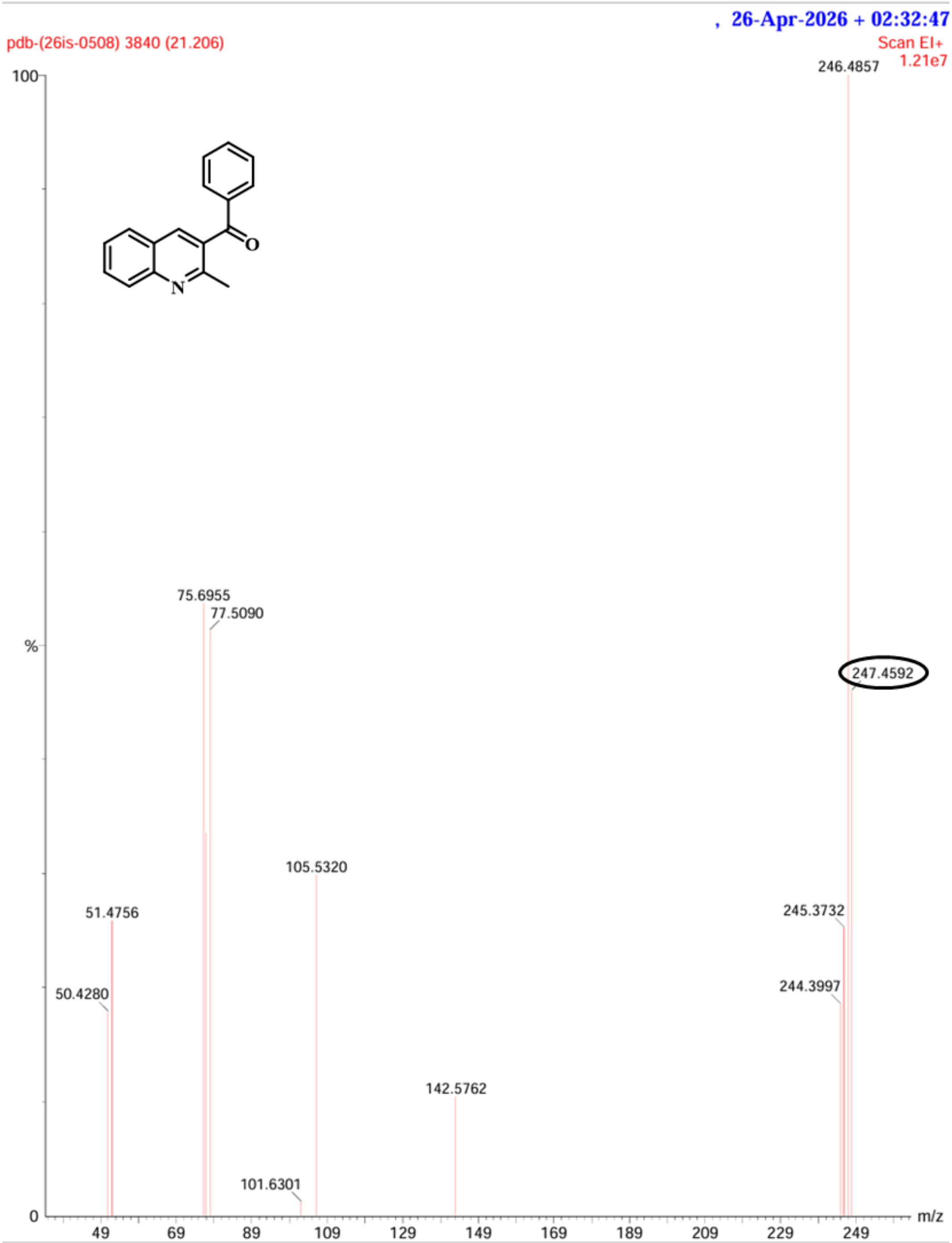

Figure S9. GC-MS spectrum of 3b

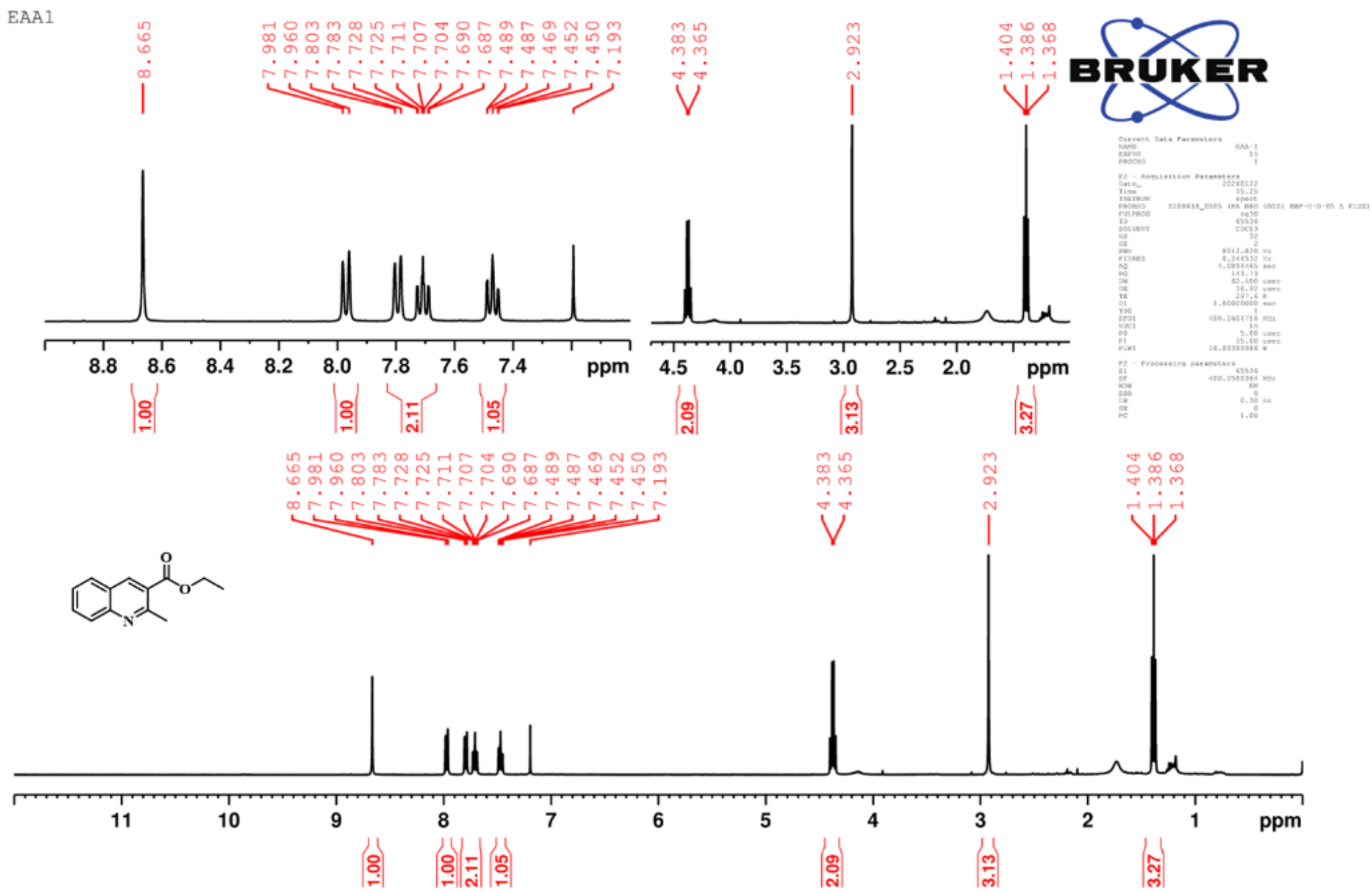

Figure S10. ¹H NMR spectrum of compound 3c

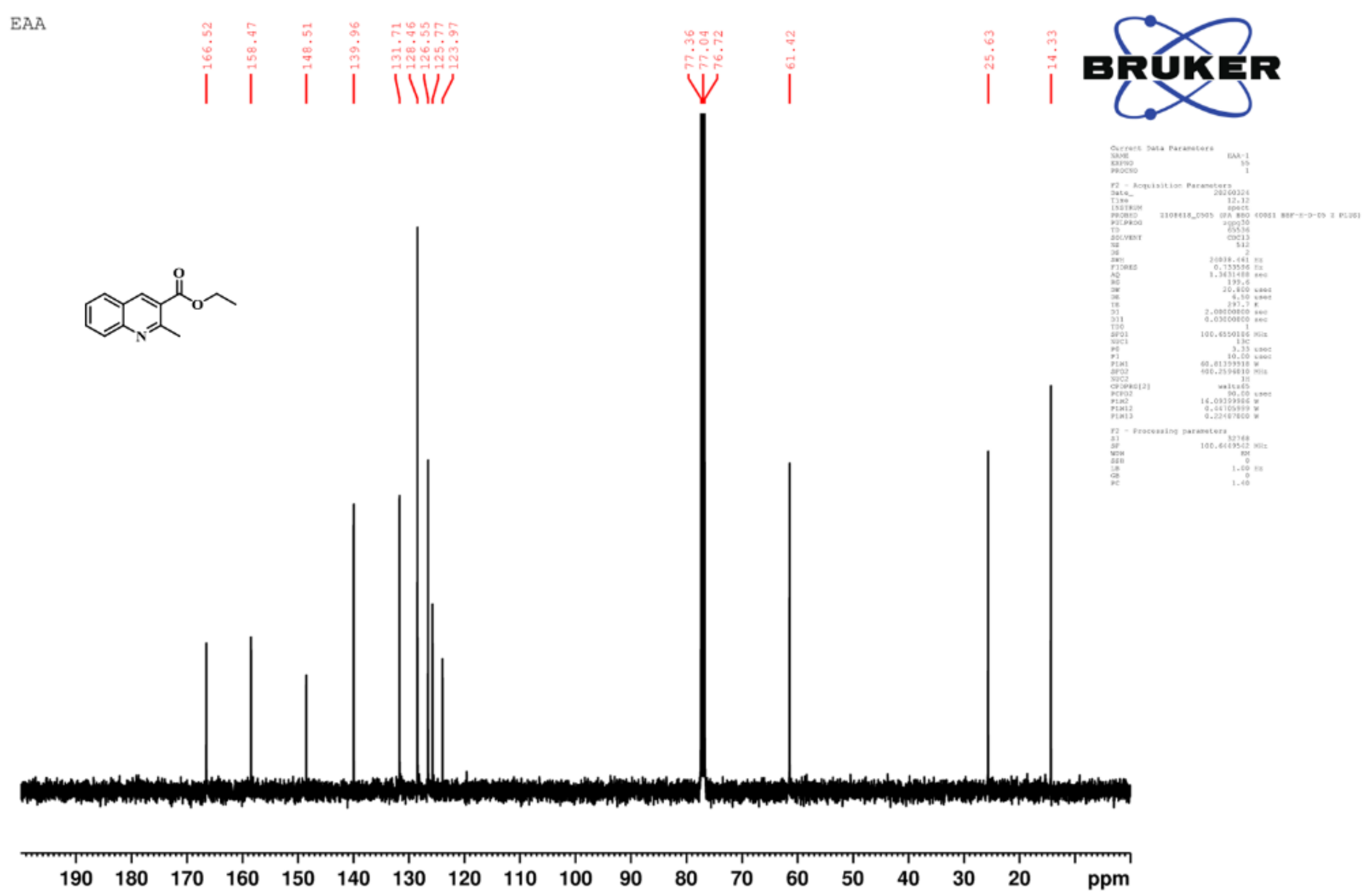

Figure S11. ^{13}C NMR spectrum of compound 3c

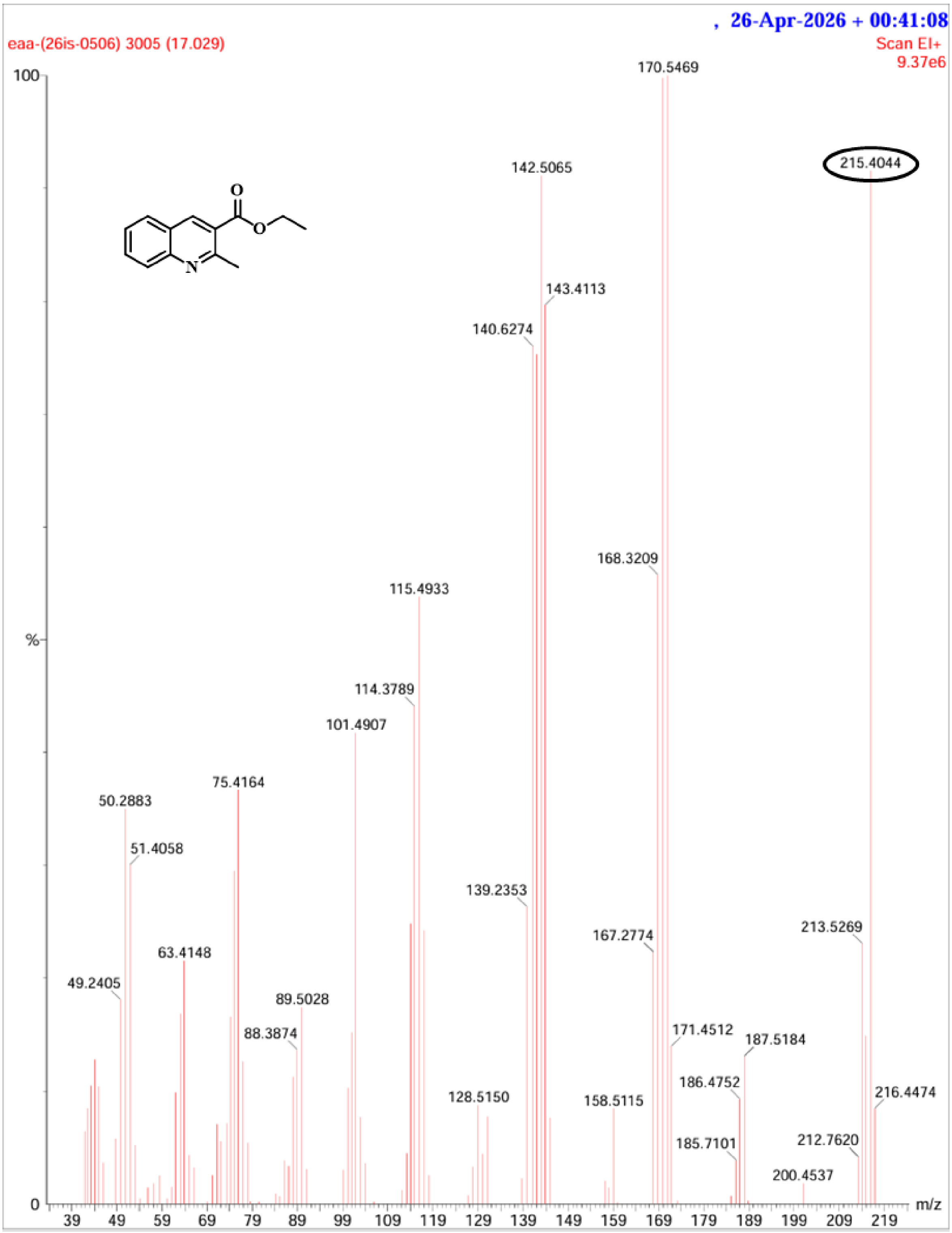

Figure S12. GC-MS spectrum of 3c

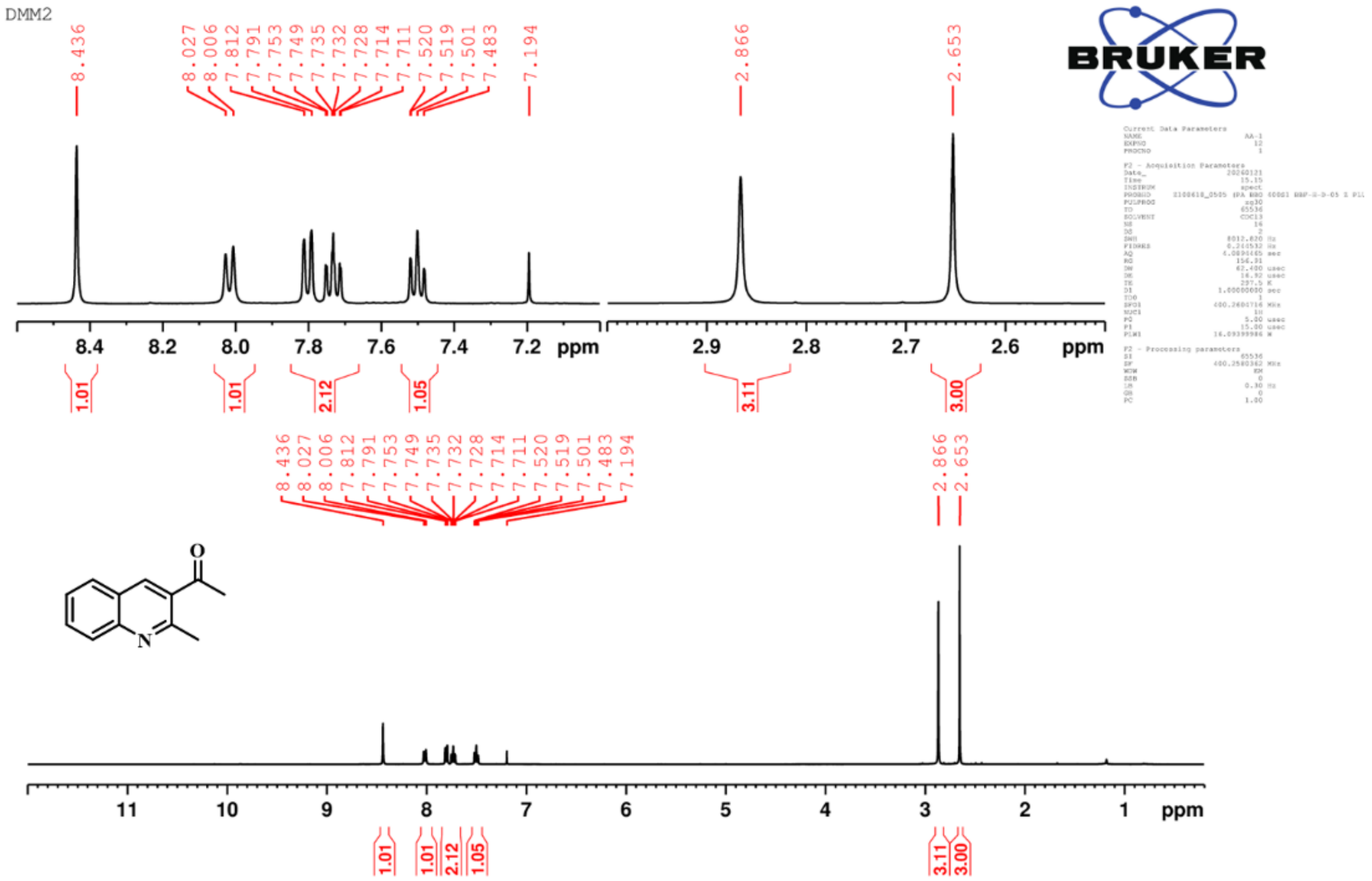

Figure S13. ^{1}H NMR spectrum of compound 3d

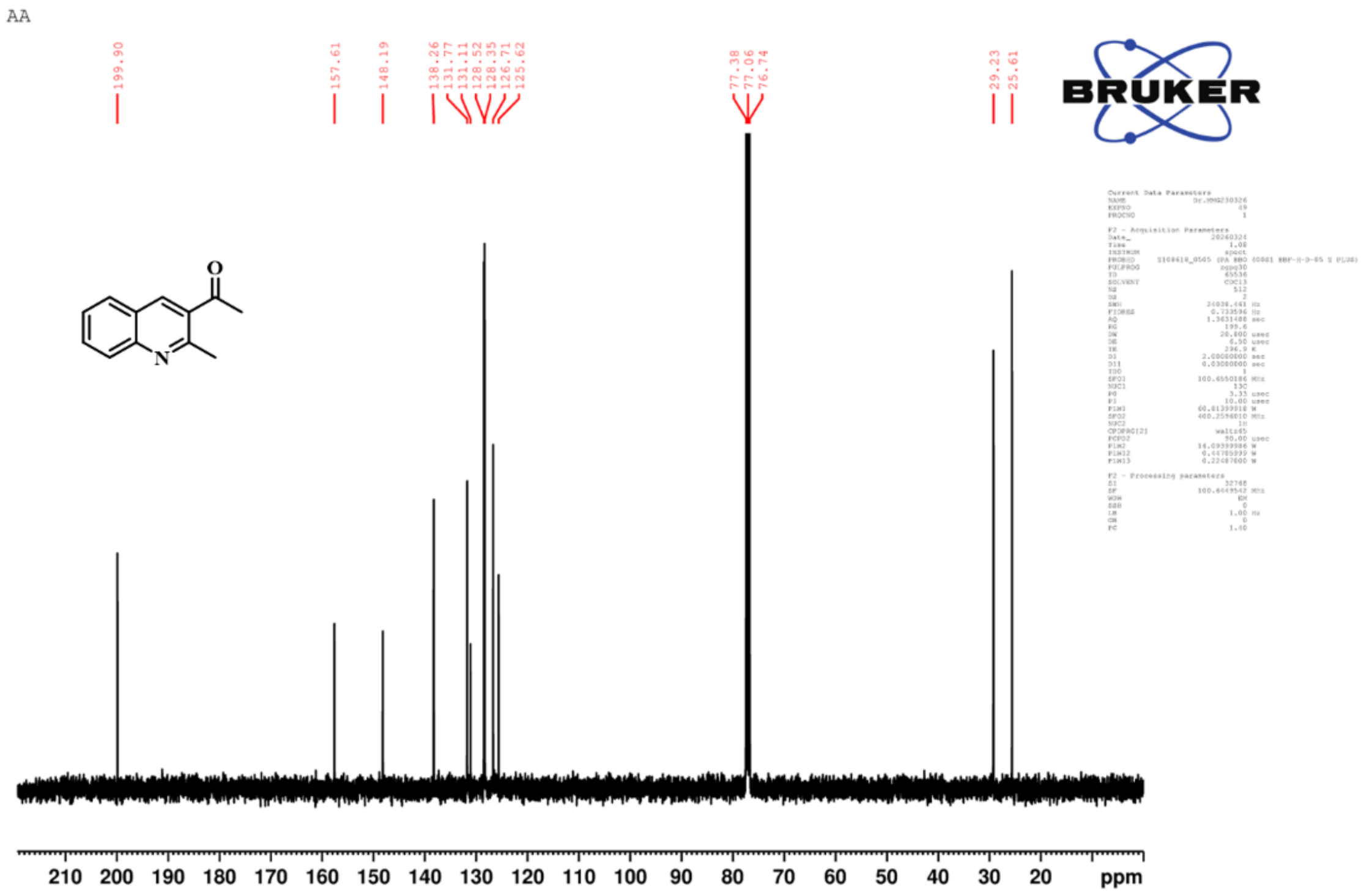

Figure S14. ^{13}C NMR spectrum of compound 3d

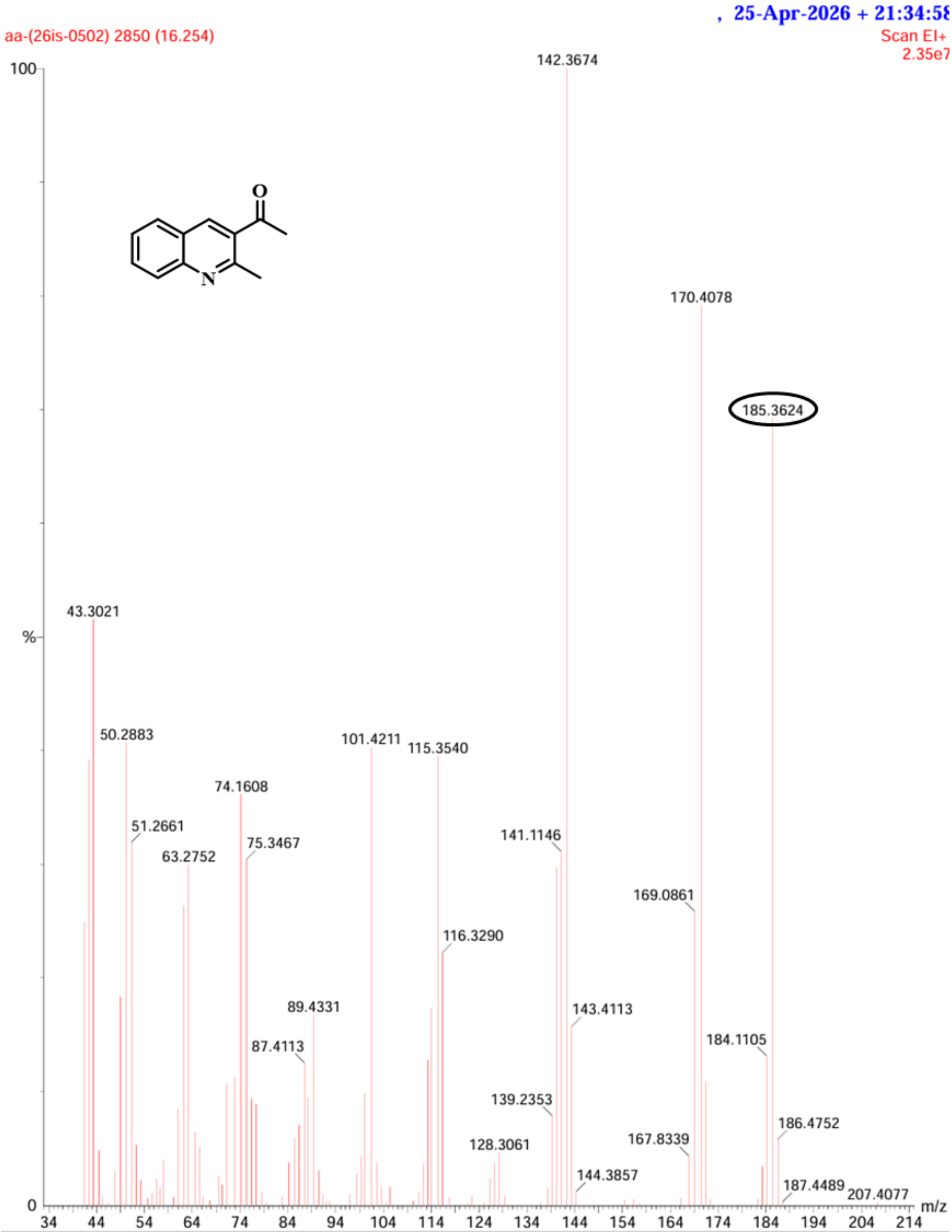

Figure S15. GC-MS spectrum of 3d

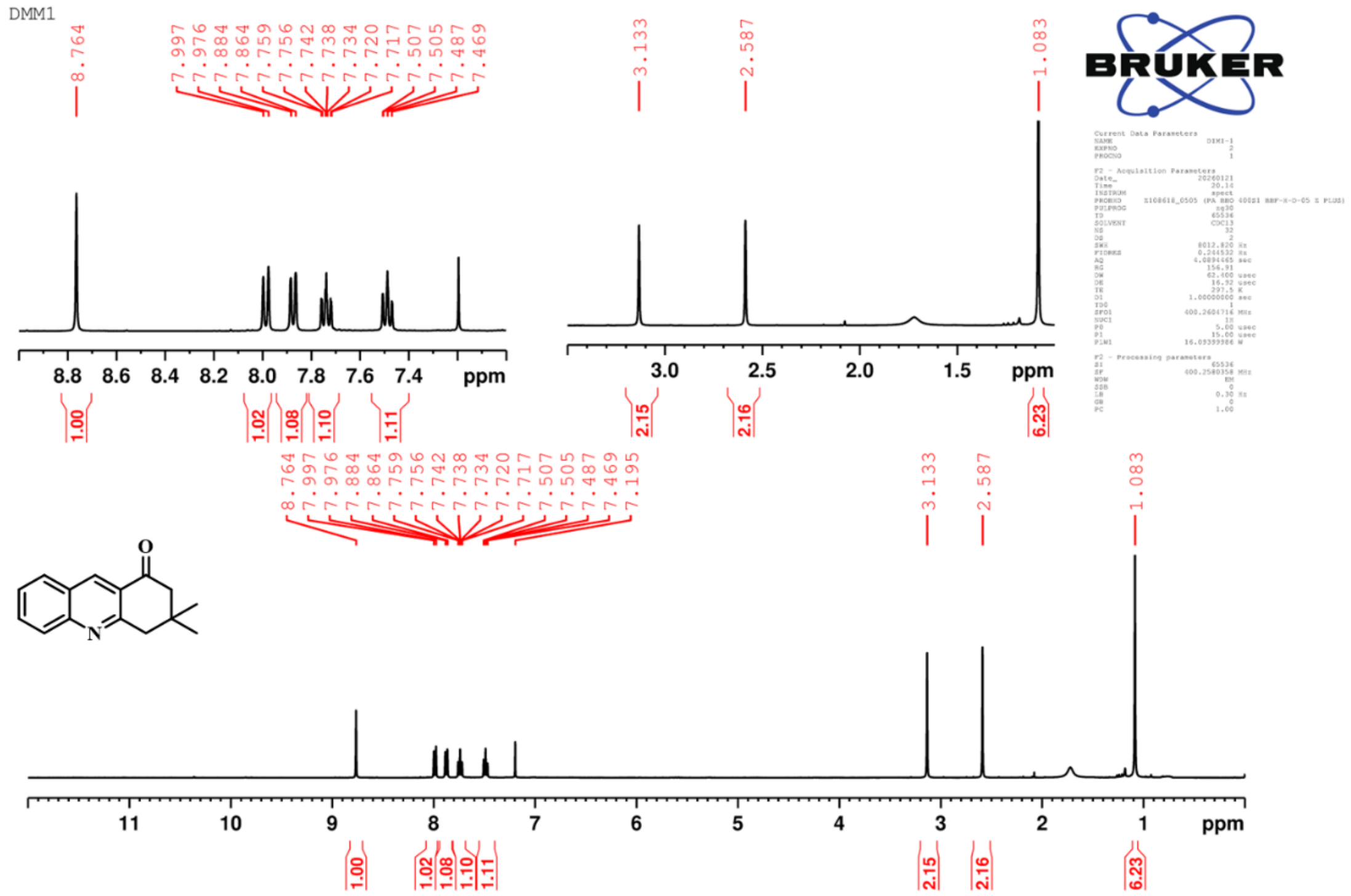

Figure S16. ^{1}H NMR spectrum of compound 3e

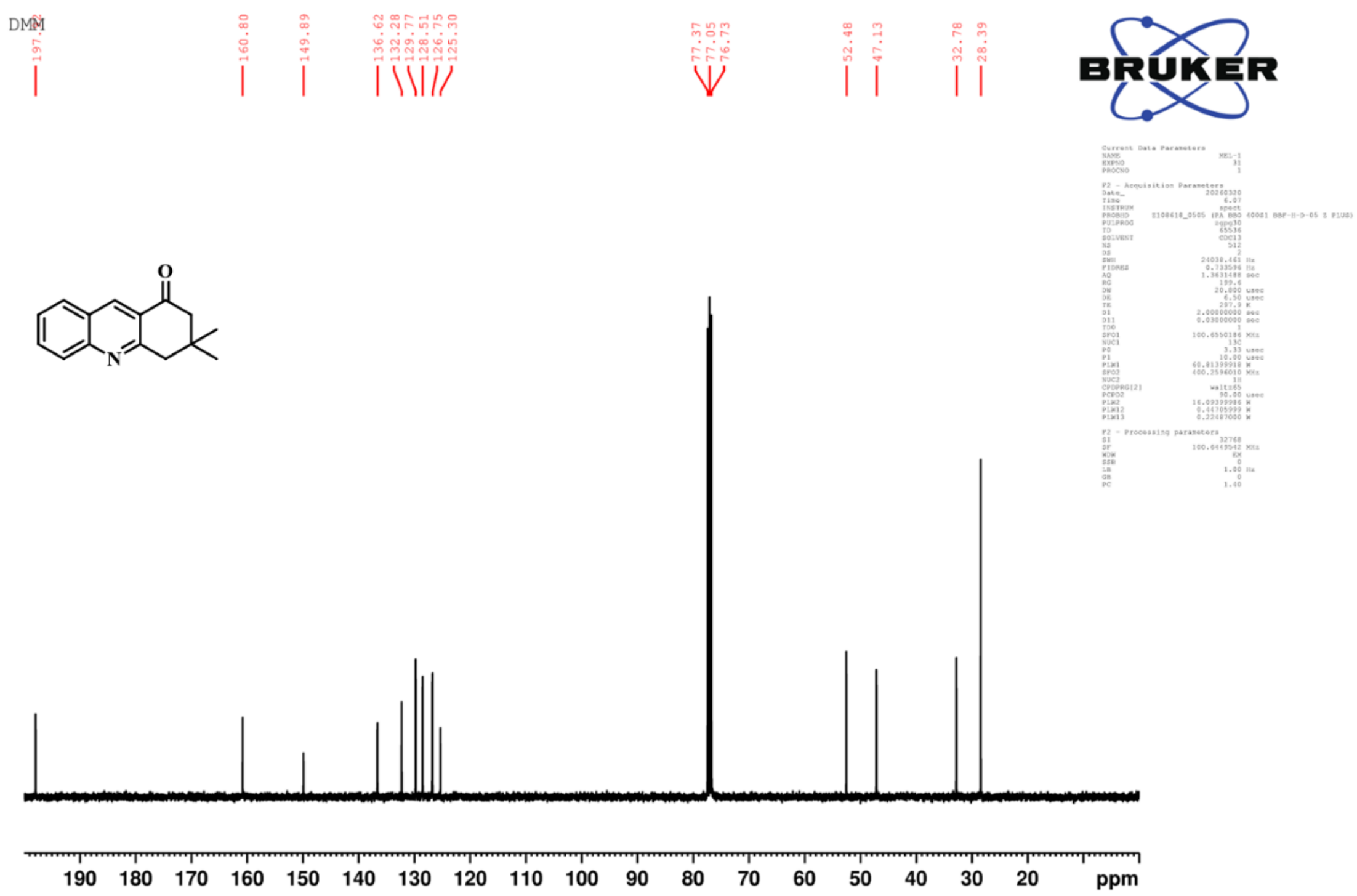

Figure S17. ^{13}C NMR spectrum of compound 3e

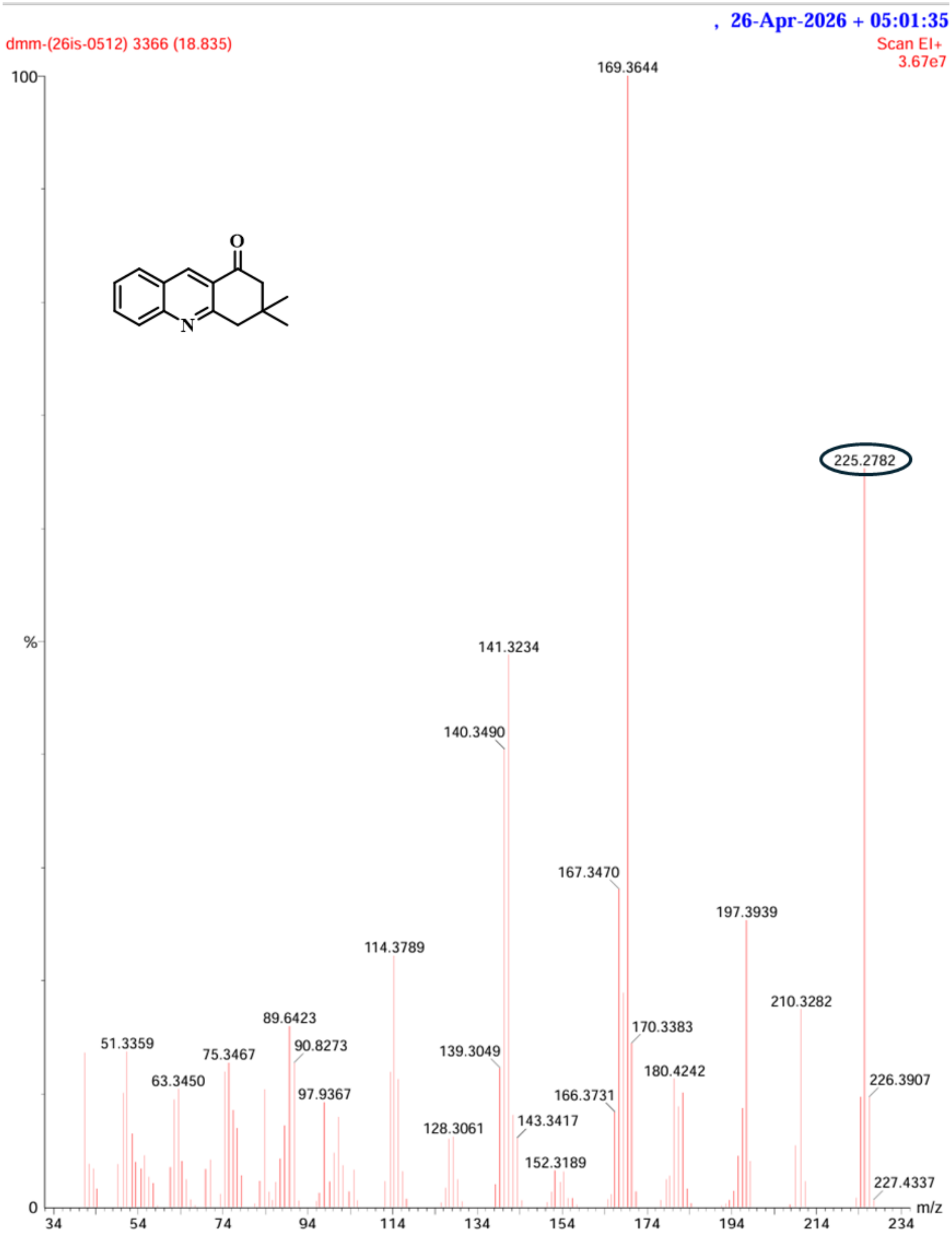

Figure S18. GC-MS spectrum of 3e

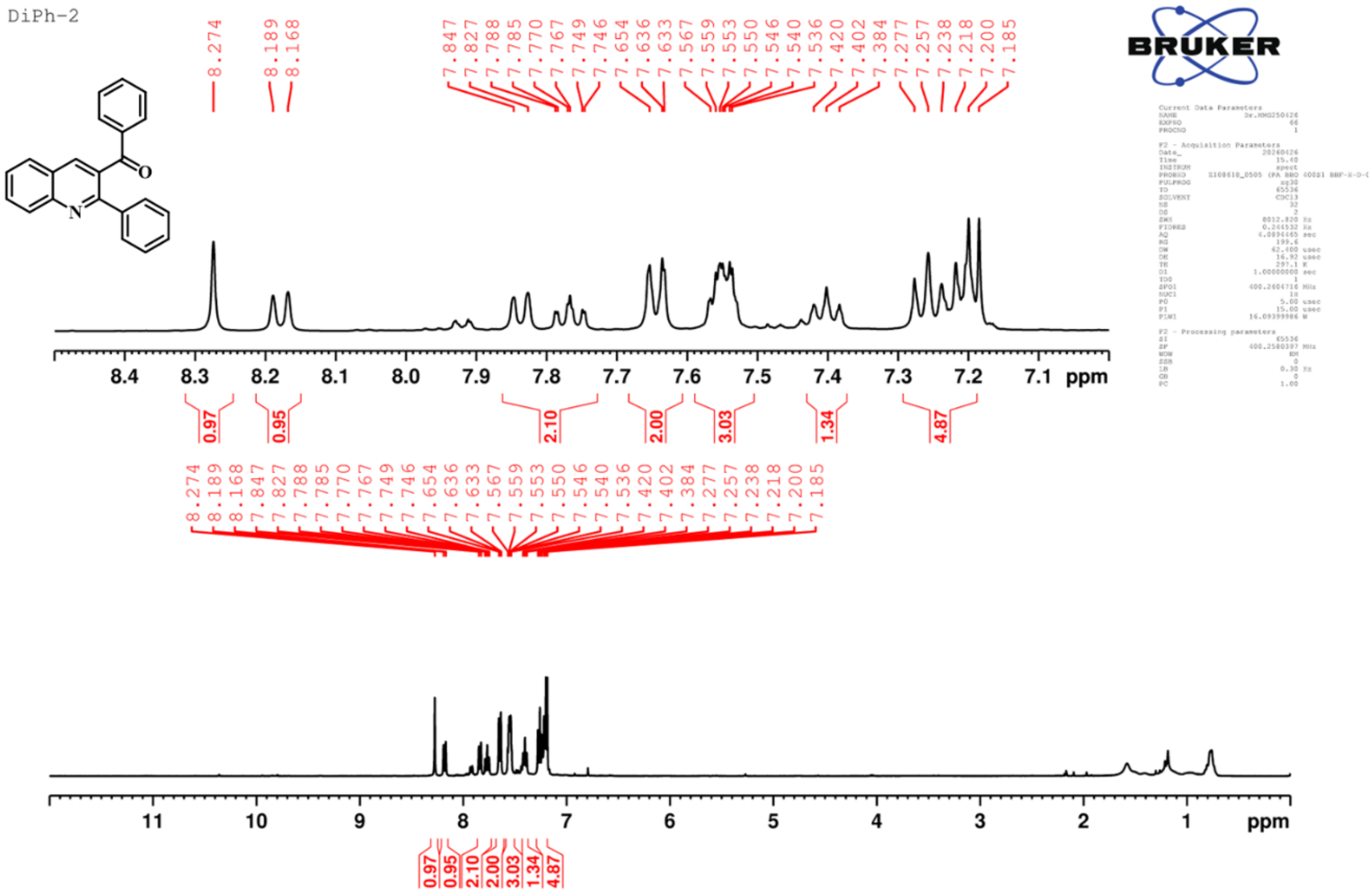

Figure S19. ^{1}H NMR spectrum of compound 3f

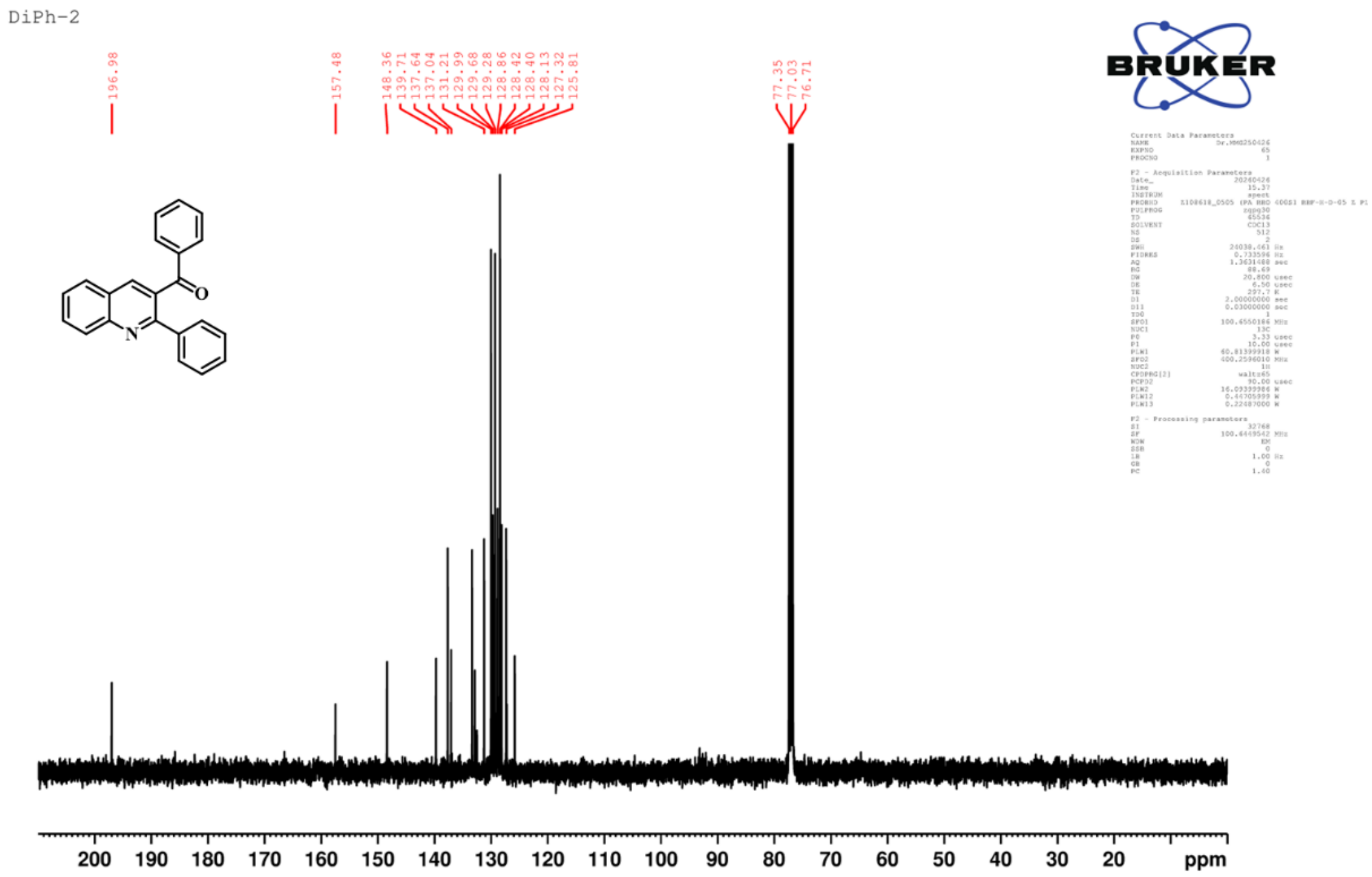

Figure S20. ^{13}C NMR spectrum of compound 3f

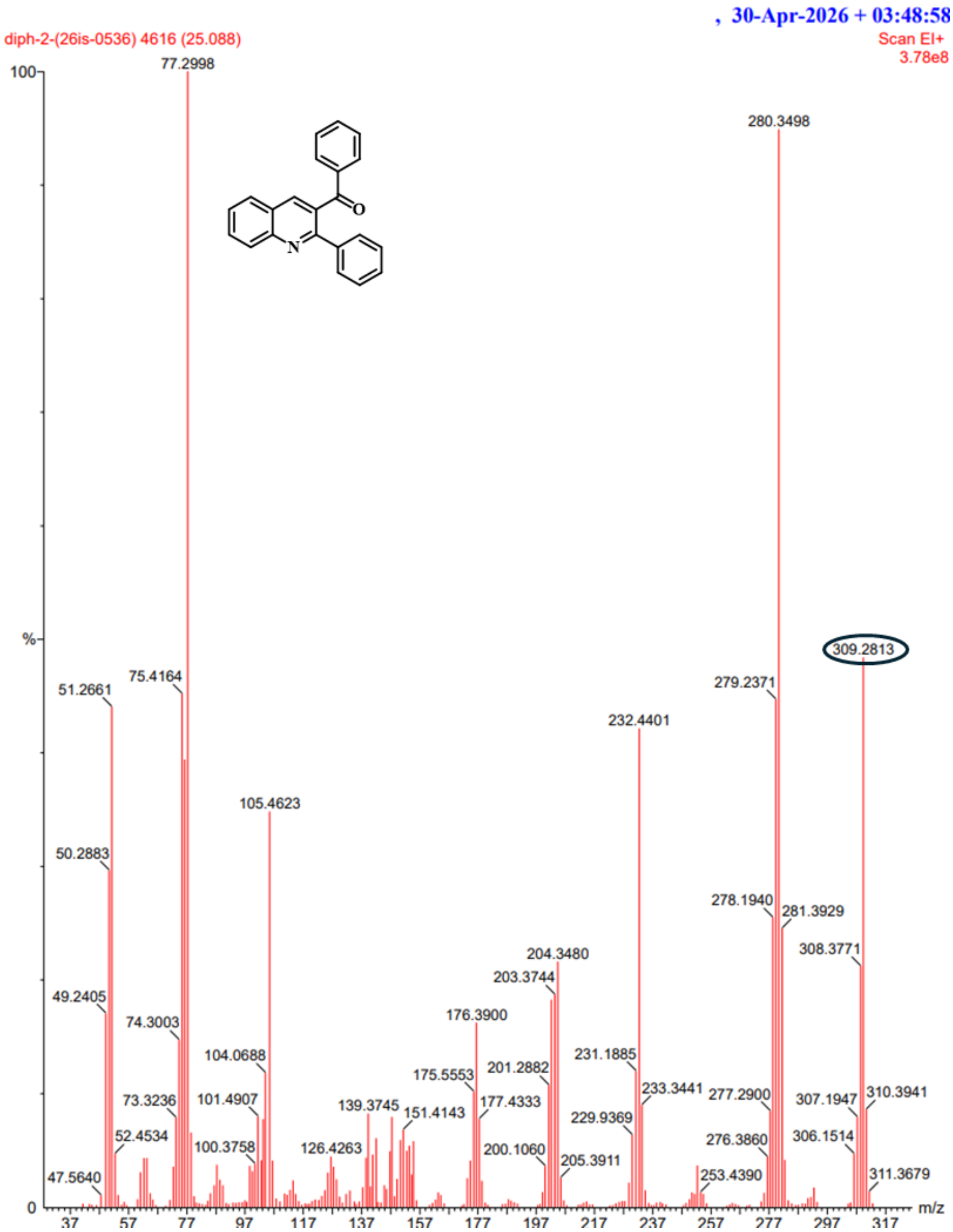

Figure S21. GC-MS spectrum of 3f

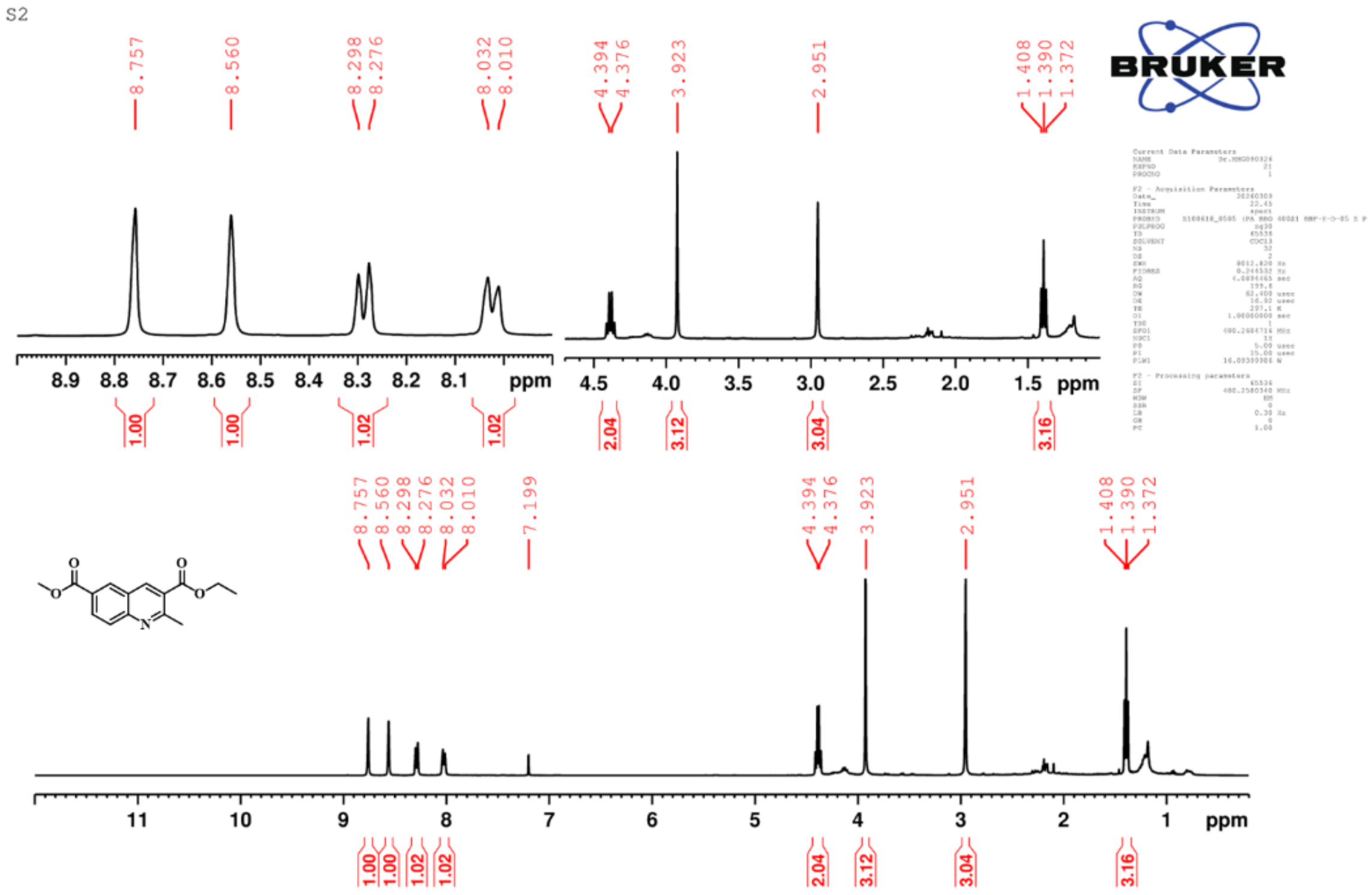

Figure S22. ^{1}H NMR spectrum of compound 3g

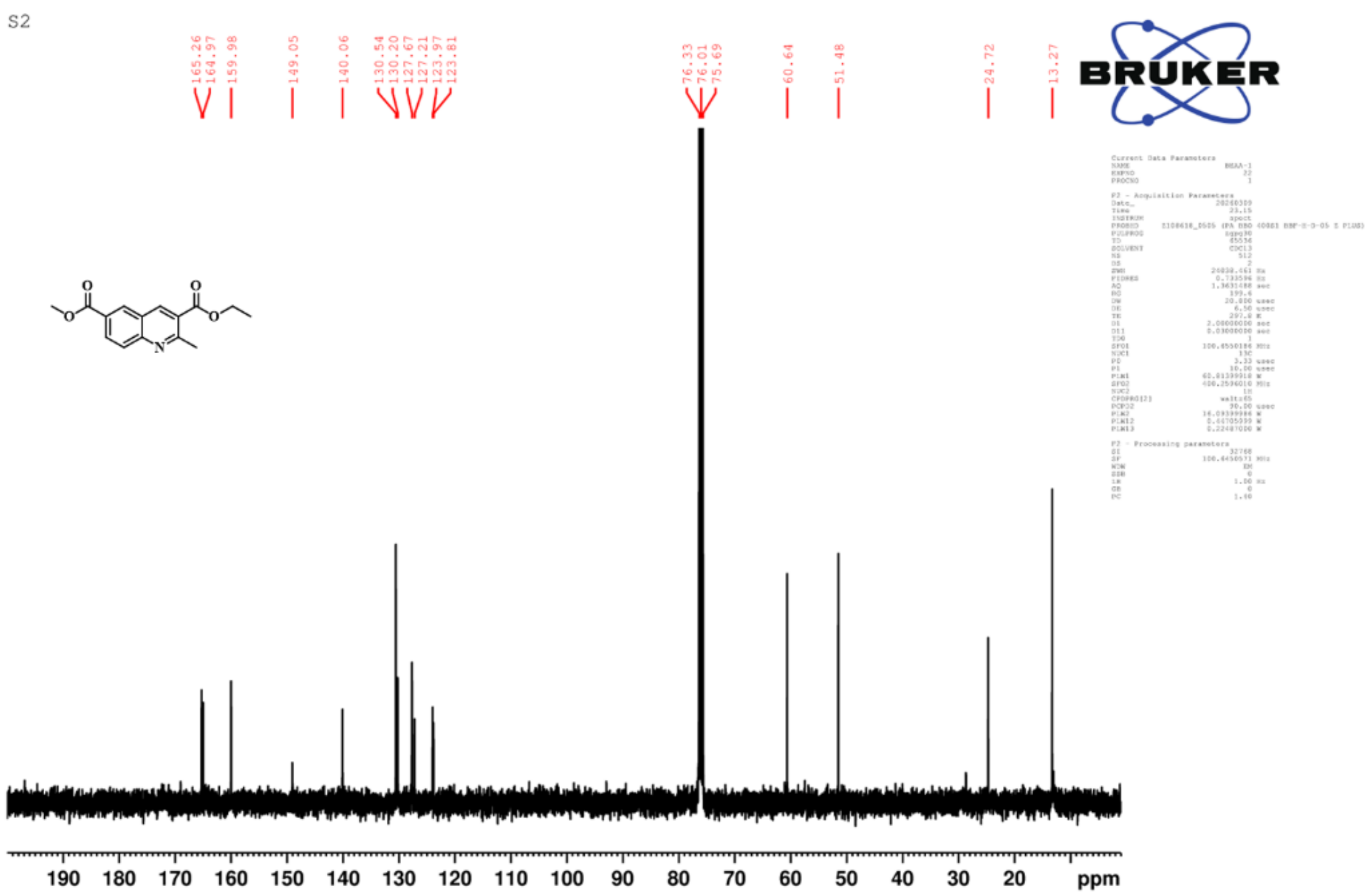

Figure S23. ^{13}C NMR spectrum of compound 3g

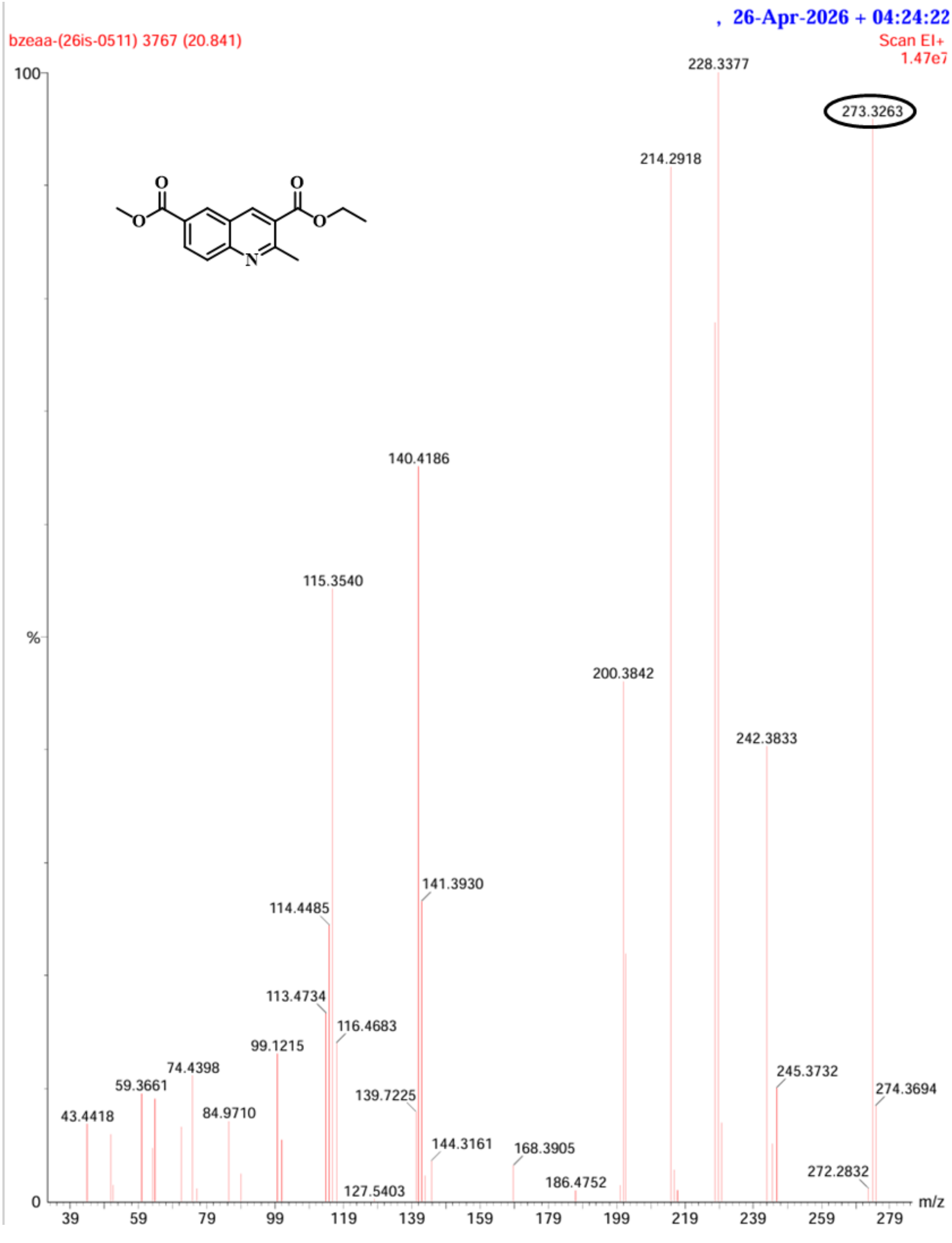

Figure S24. GC-MS spectrum of 3g

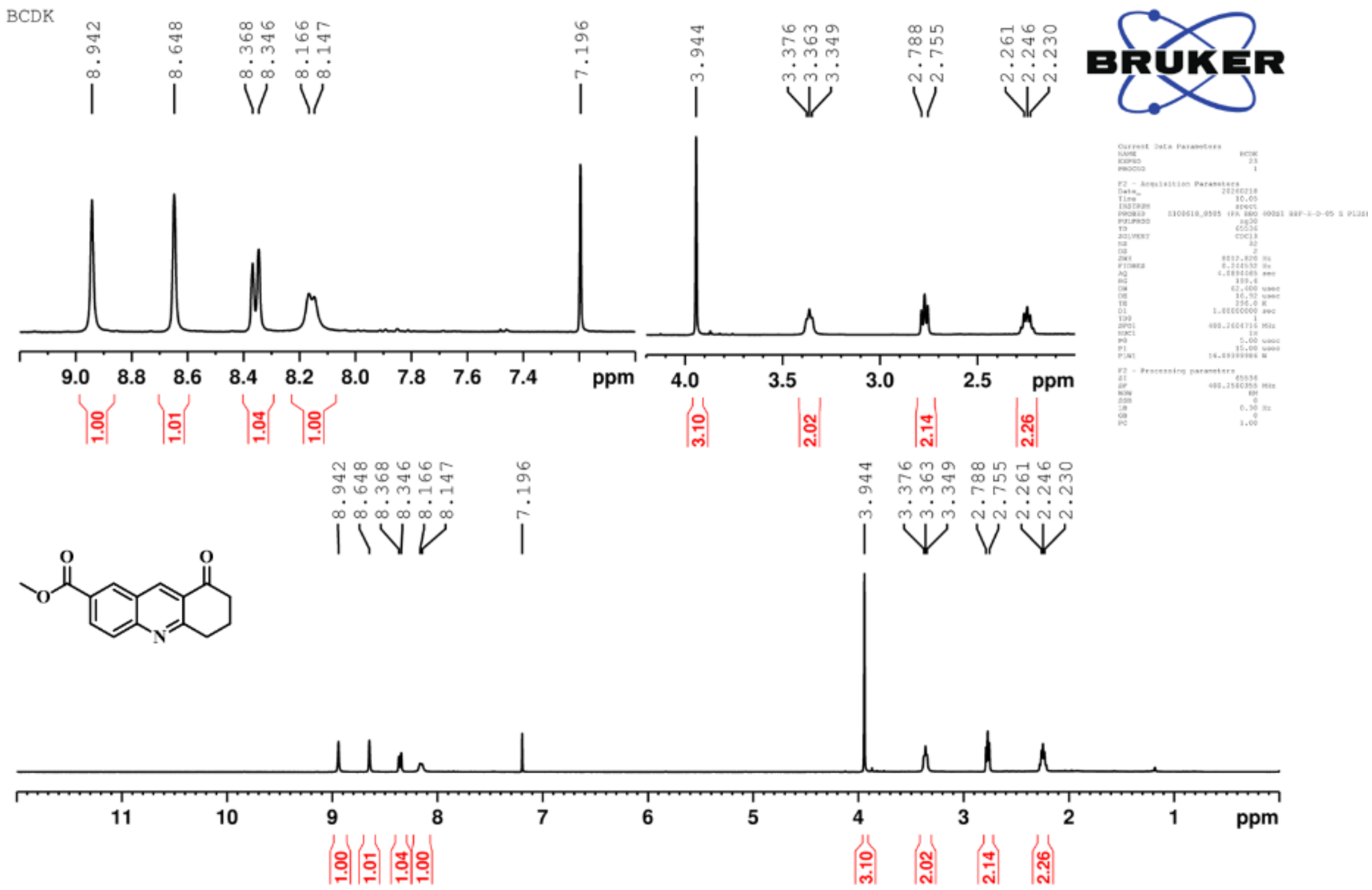

Figure S25. ^{1}H NMR spectrum of compound 3h

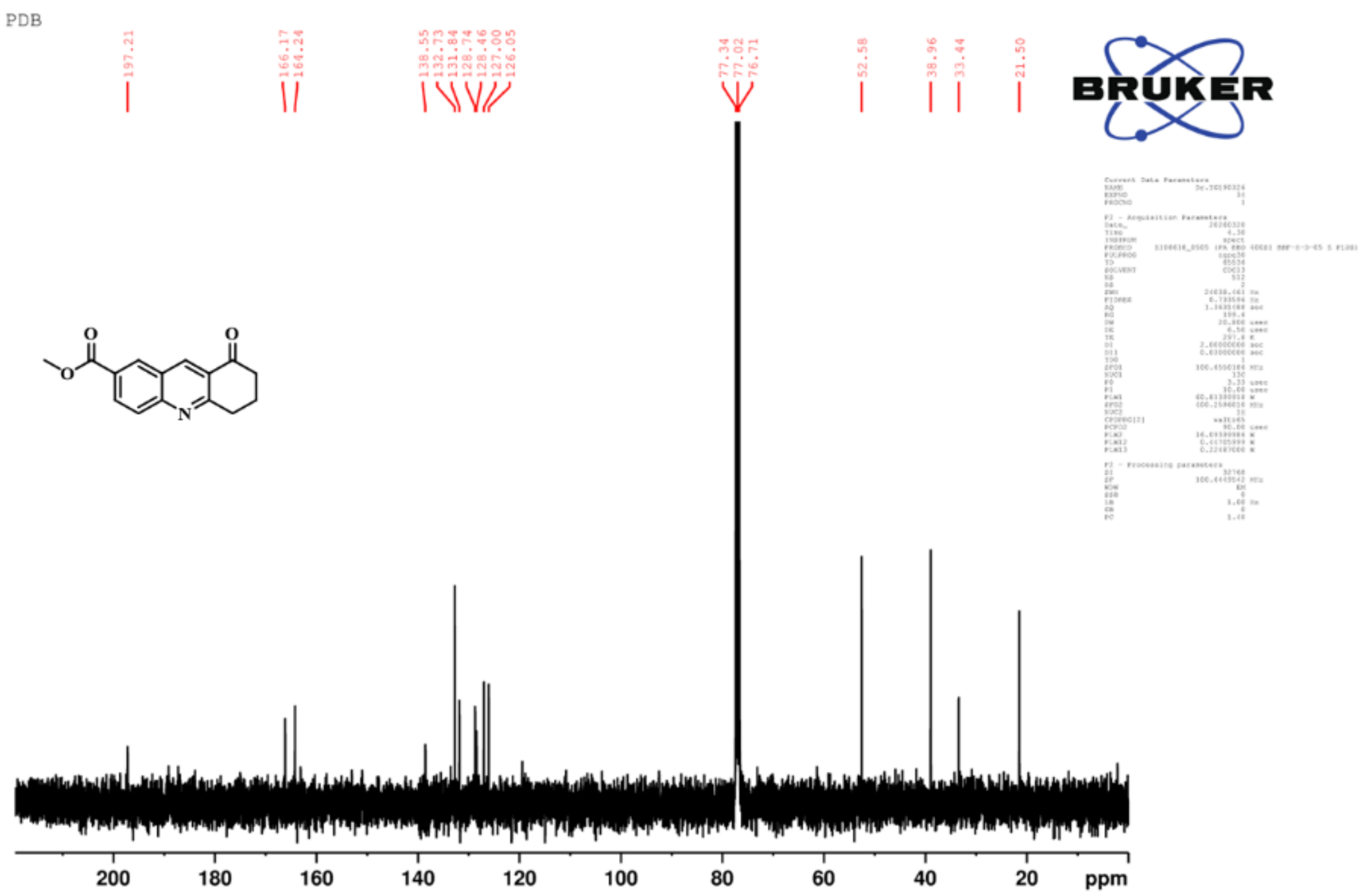

Figure S26. ^{13}C NMR spectrum of compound 3h

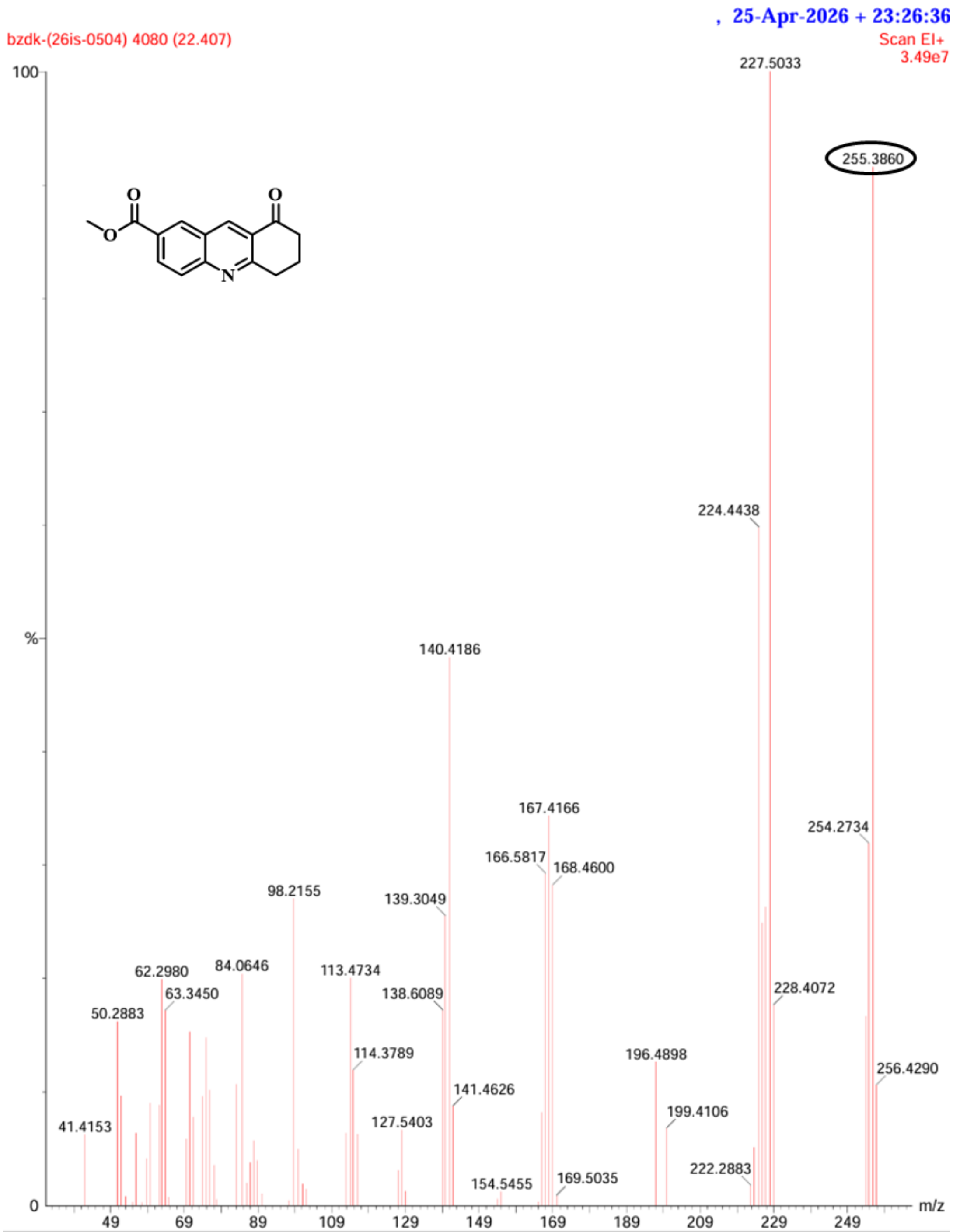

Figure S27. GC-MS spectrum of 3h

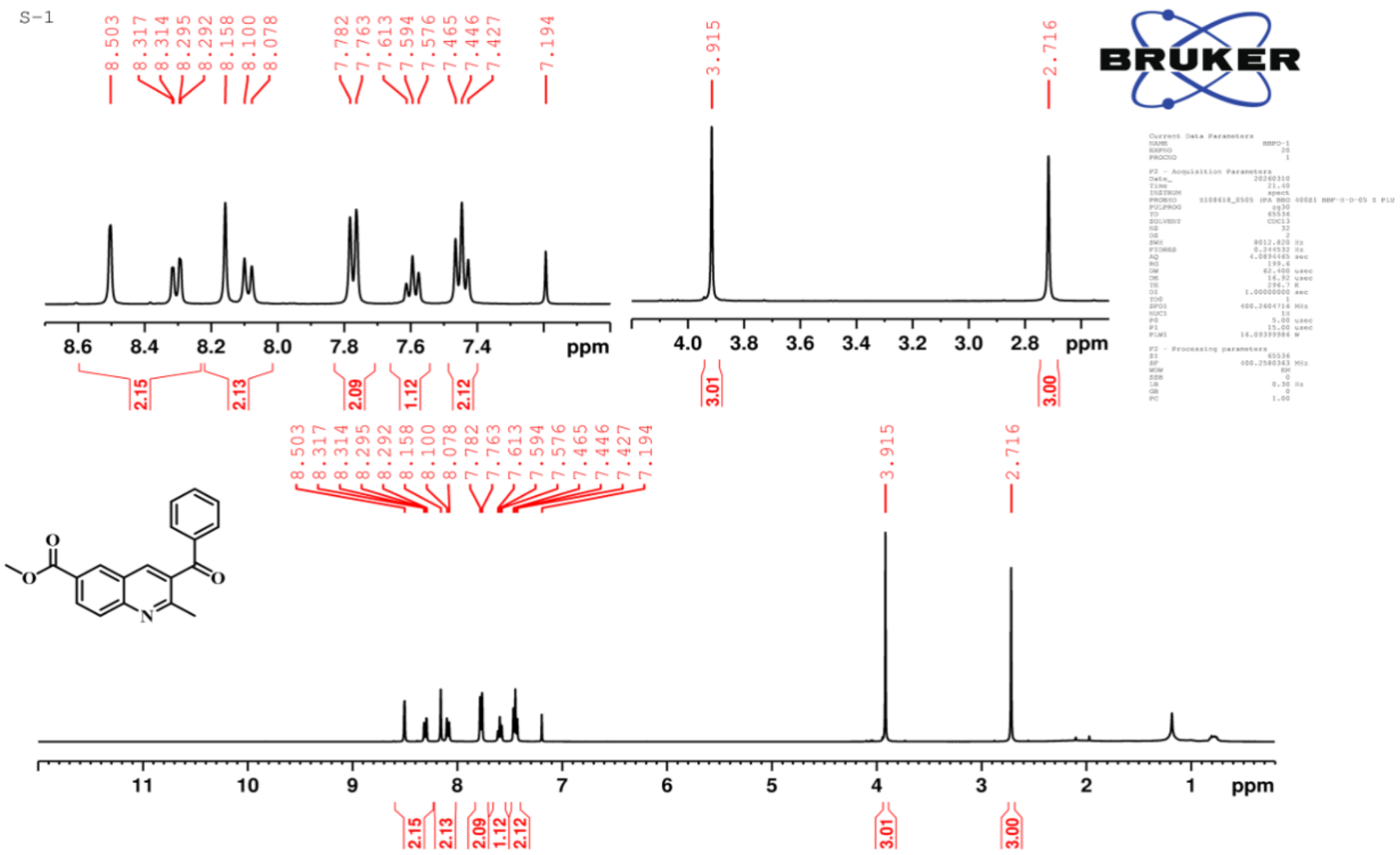

Figure S28. ^{1}H NMR spectrum of compound 3i

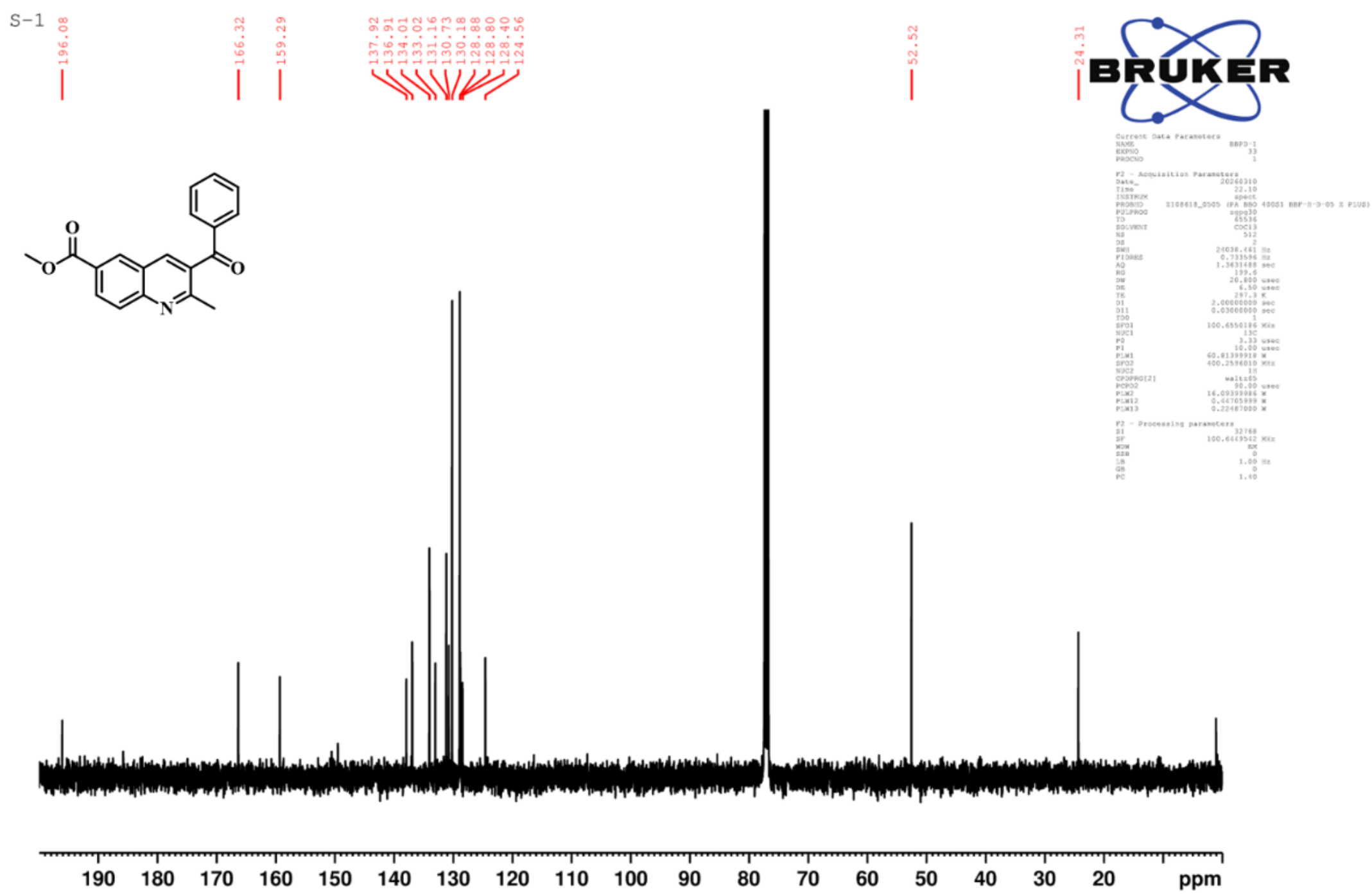

Figure S29. ^{13}C NMR spectrum of compound 3i

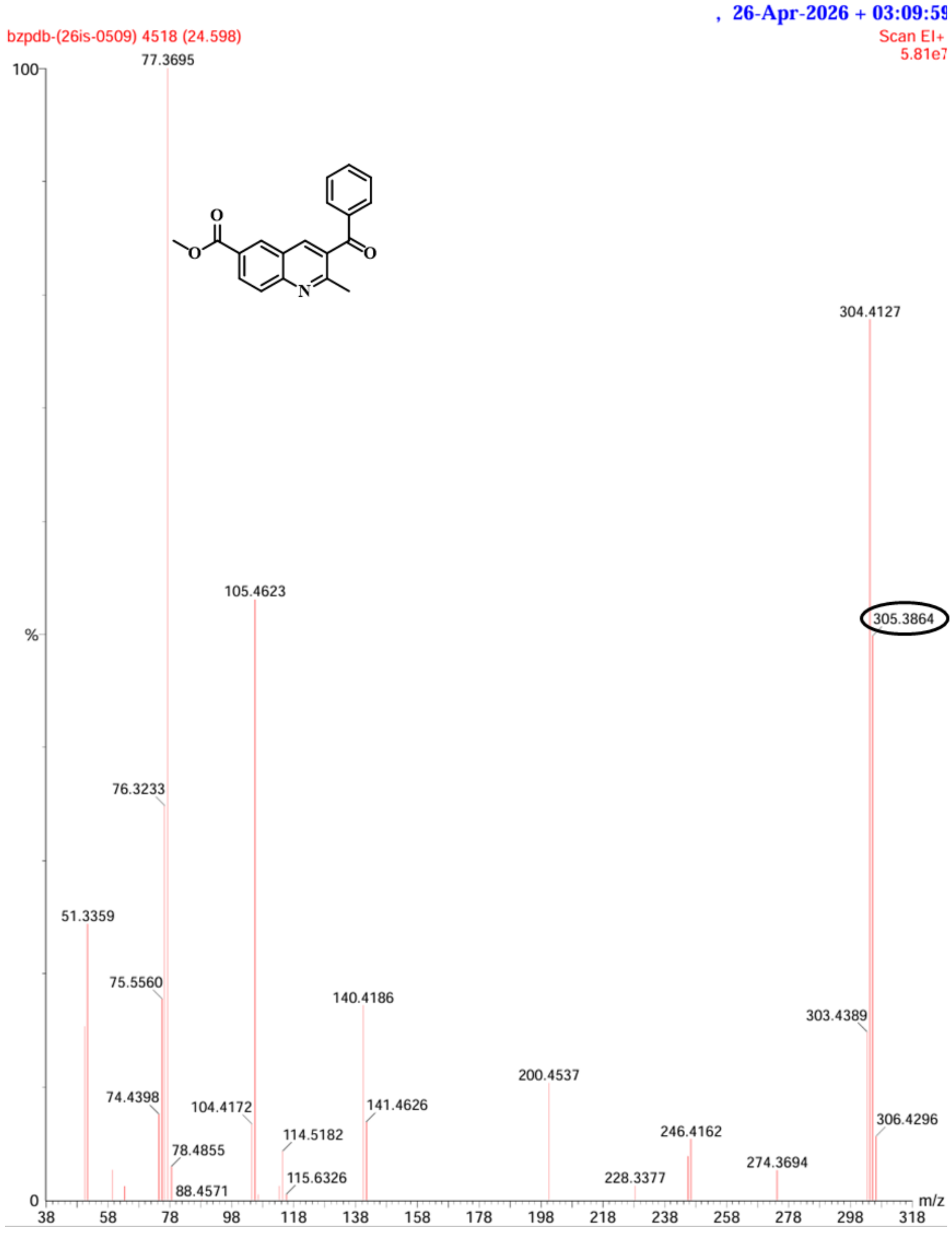

Figure S30. GC-MS spectrum of 3i

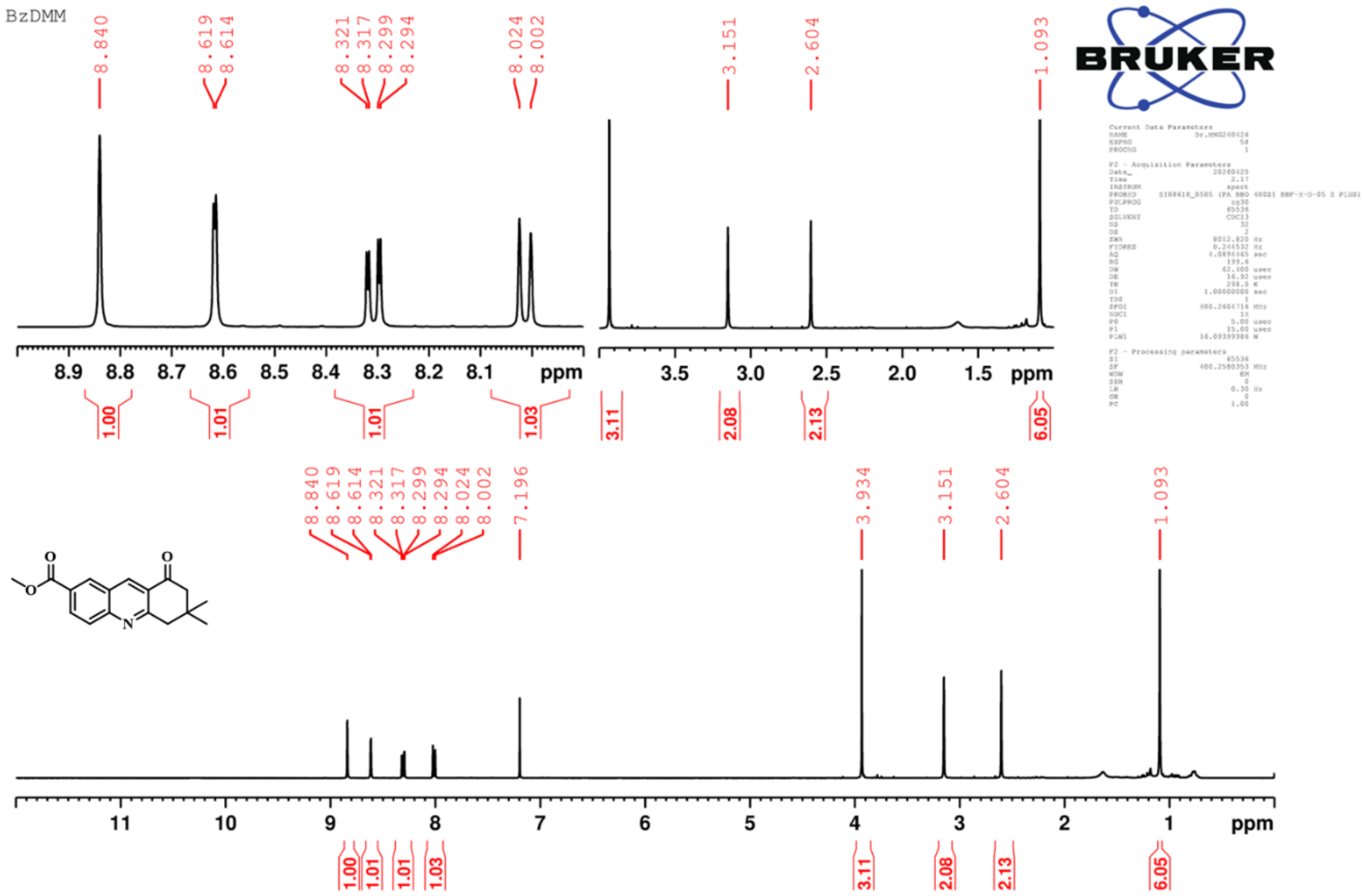

Figure S31. ^{1}H NMR spectrum of compound 3j

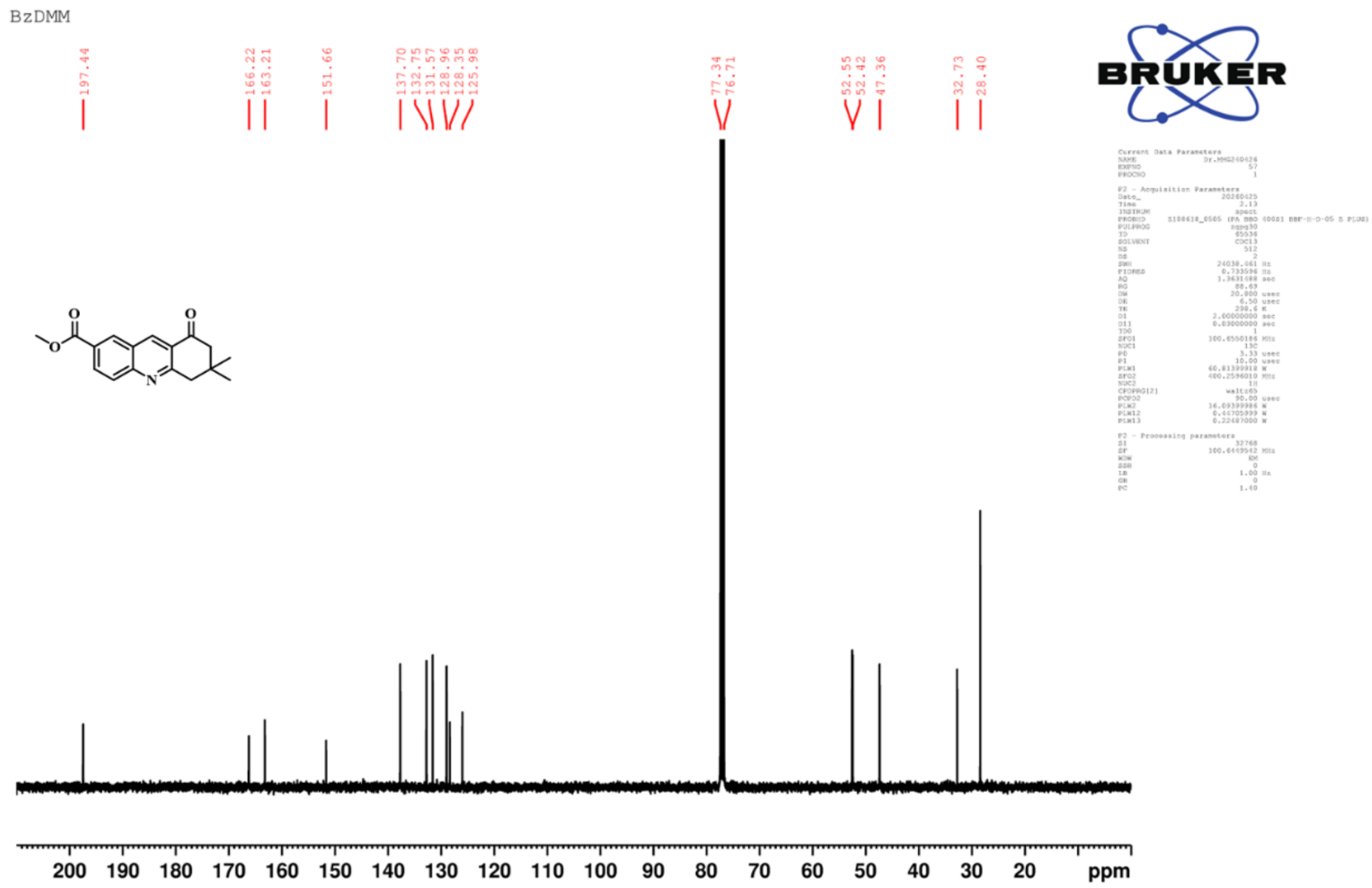

Figure S32. ^{13}C NMR spectrum of compound 3j

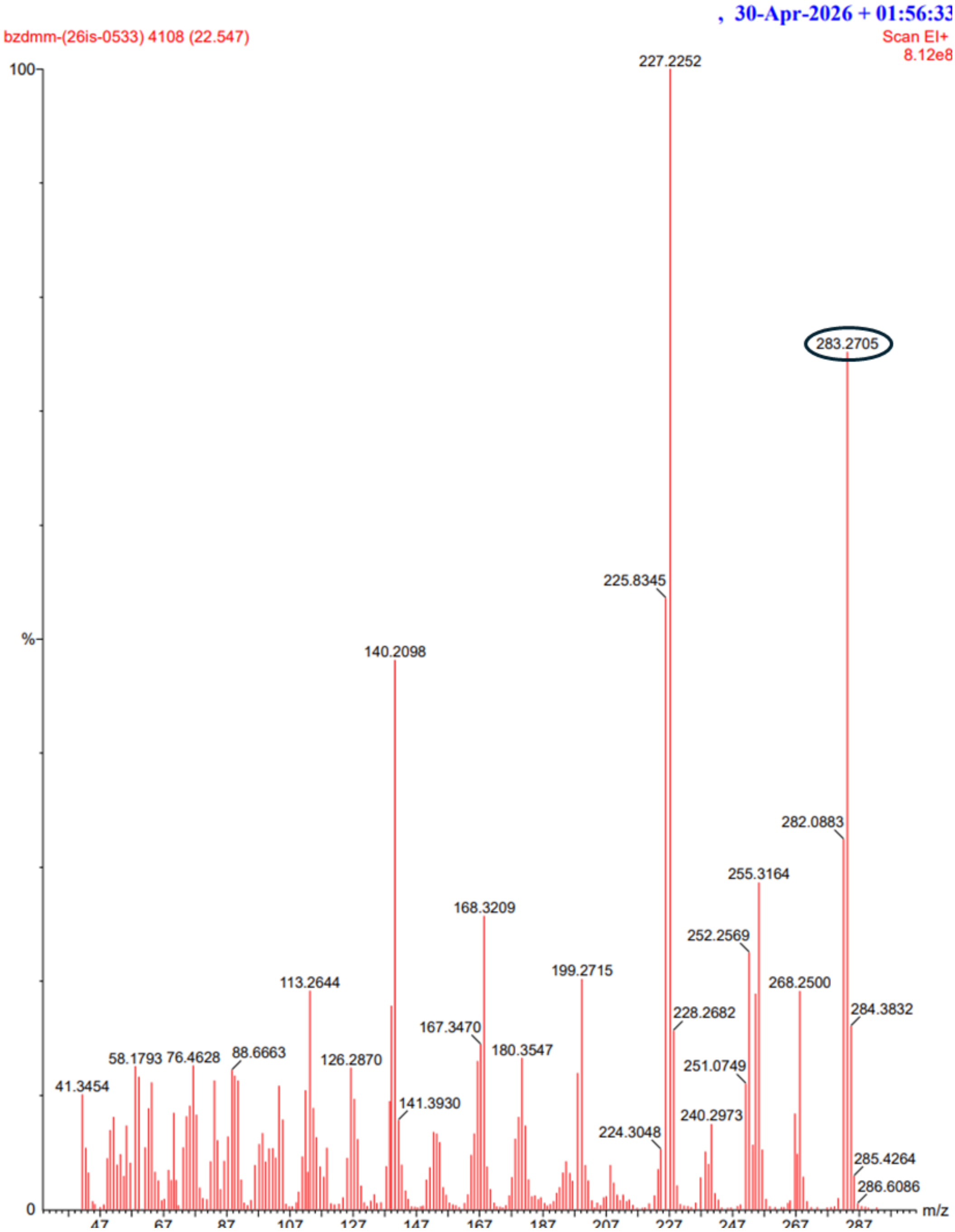

Figure S33. GC-MS spectrum of 3j

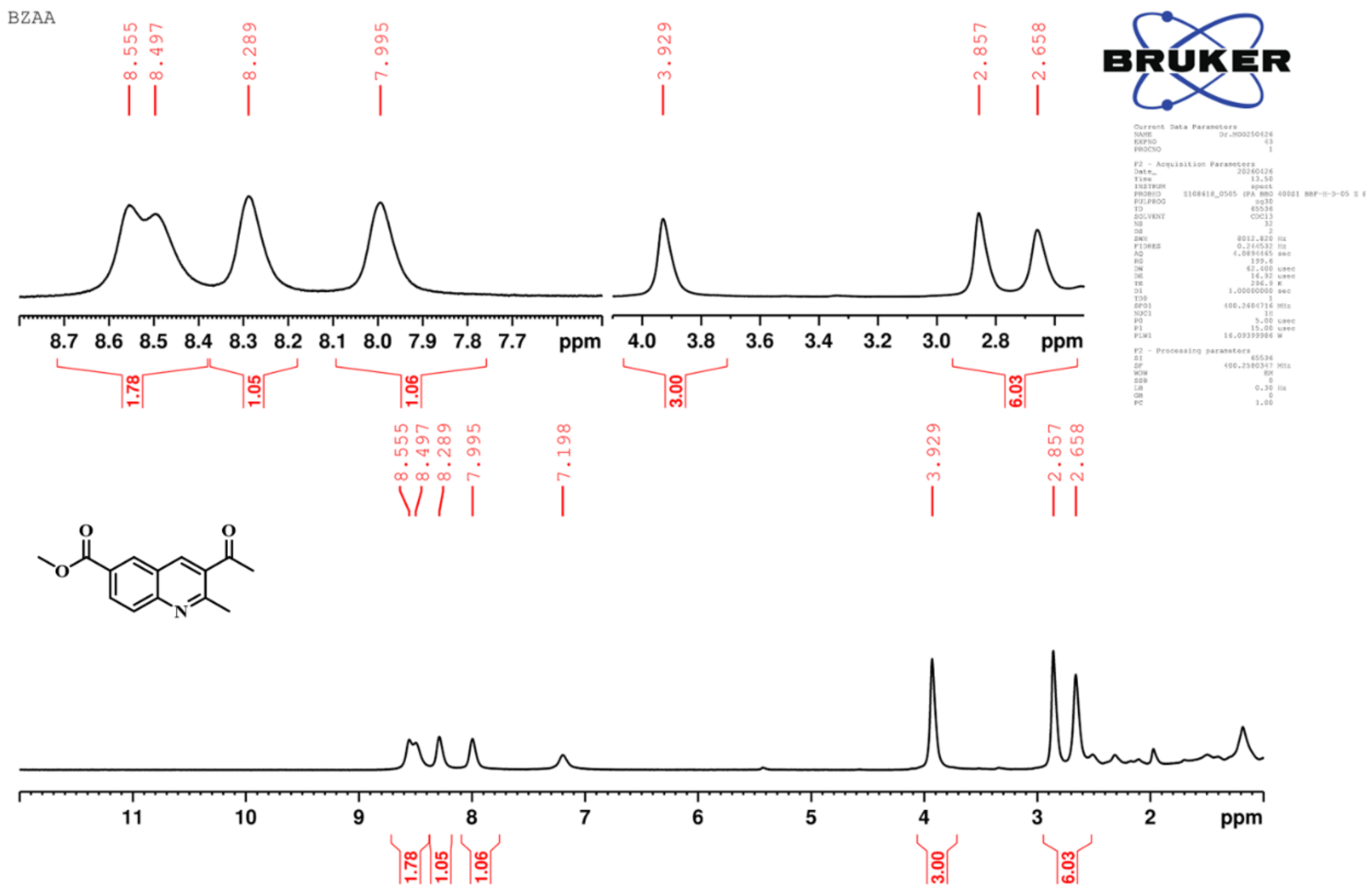

Figure S34. ^{1}H NMR spectrum of compound 3k

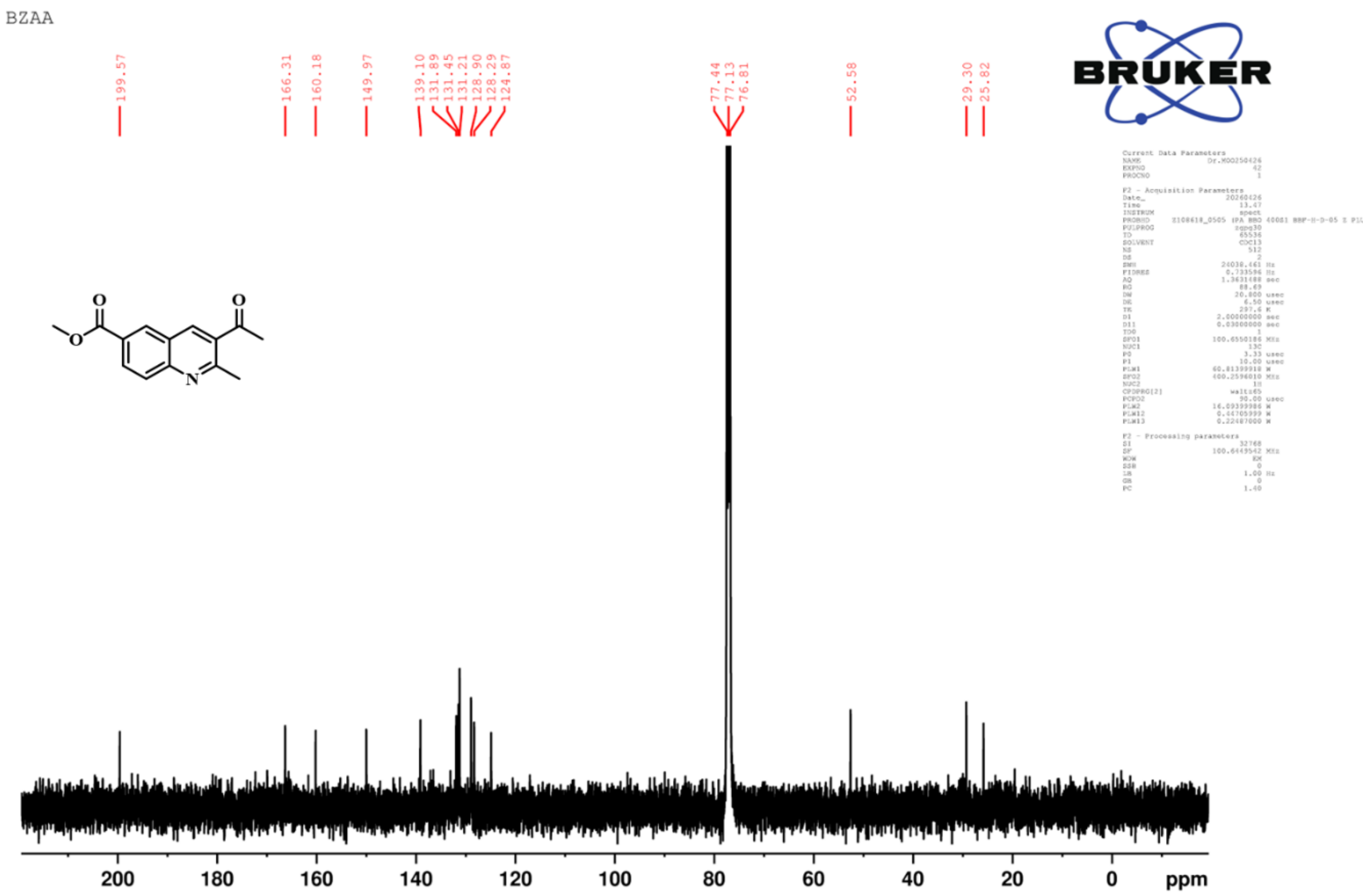

Figure S35. ^{13}C NMR spectrum of compound 3k

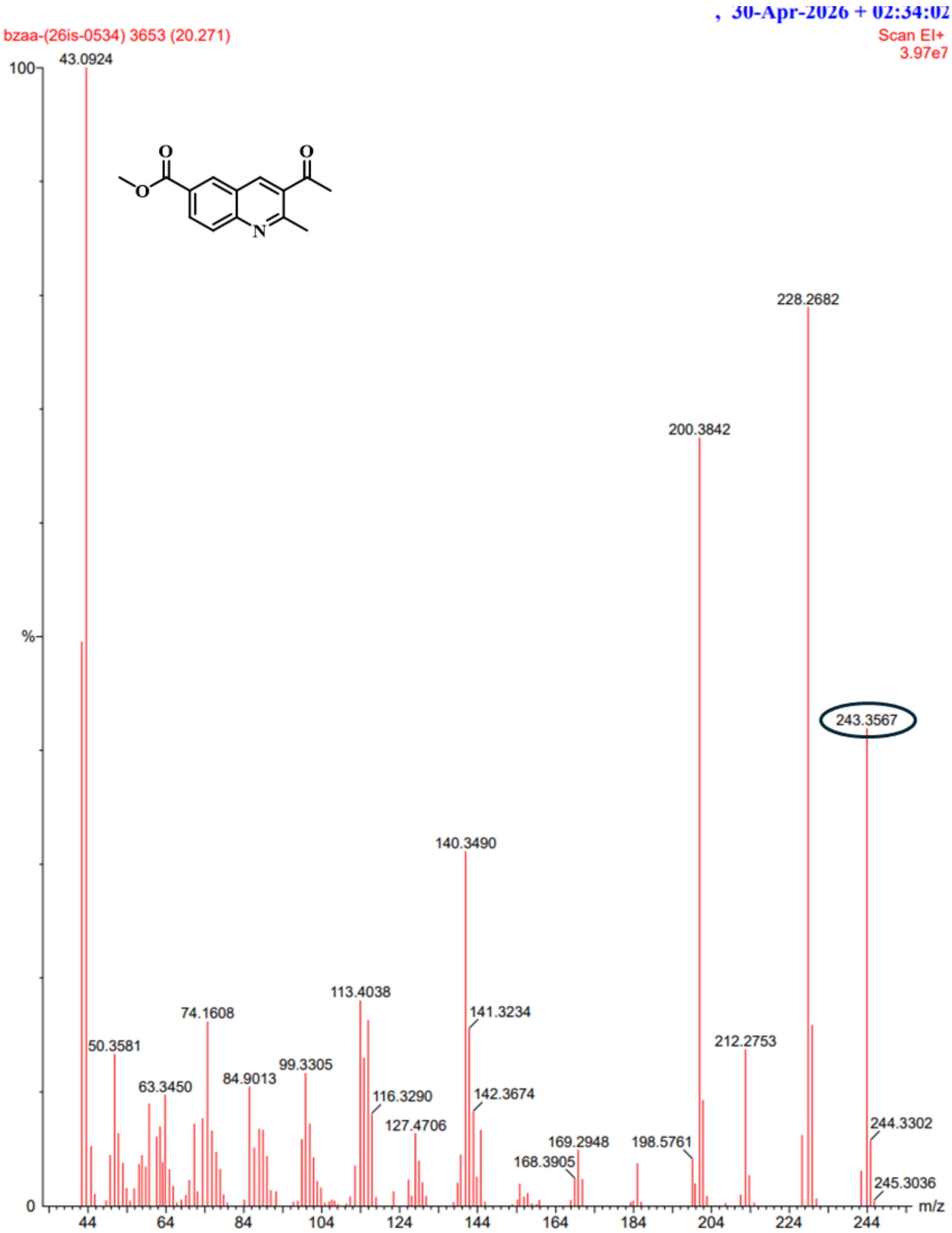

Figure S36. GC-MS spectrum of 3k

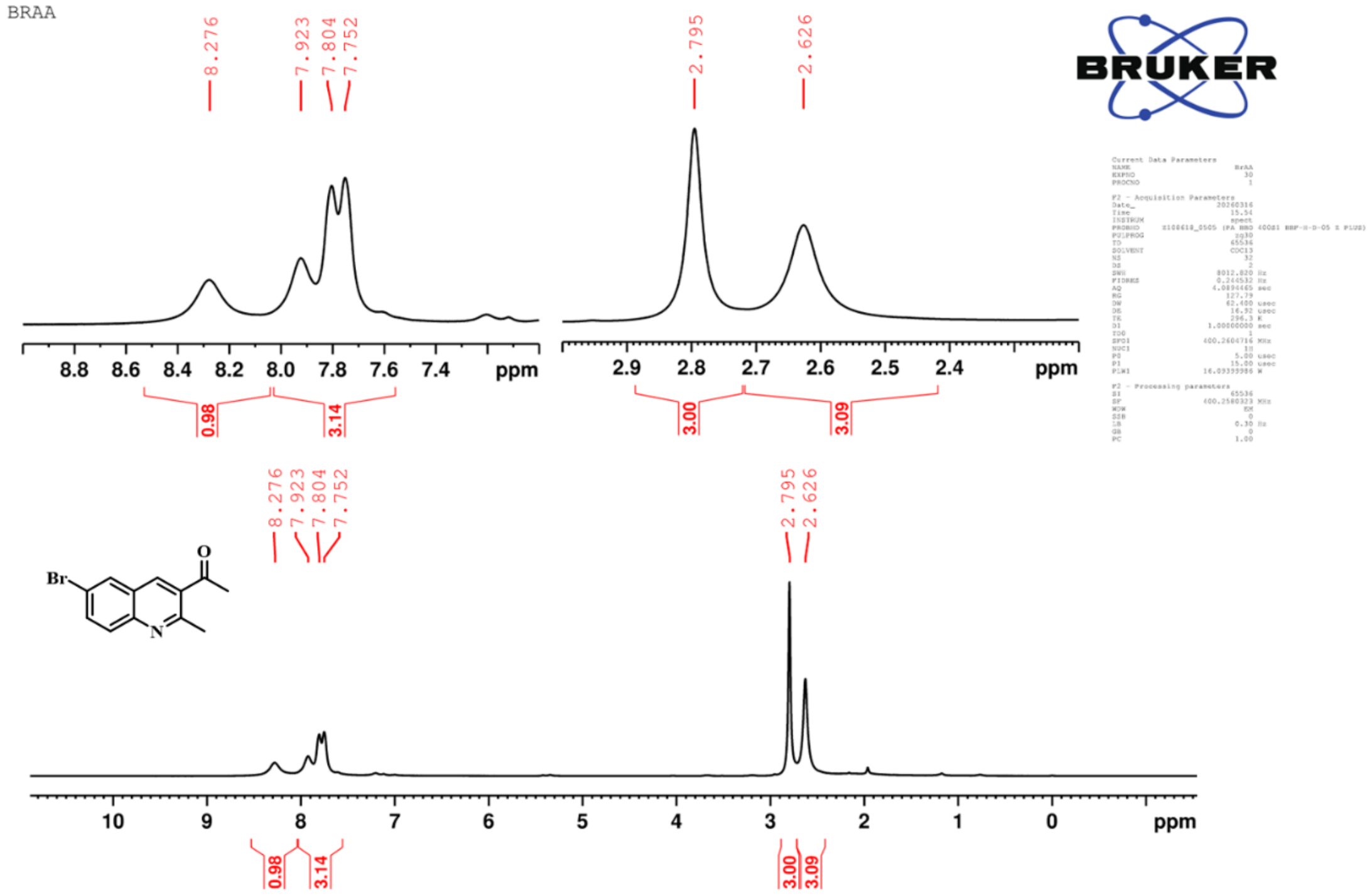

Figure S37. ^{1}H NMR spectrum of compound 3l

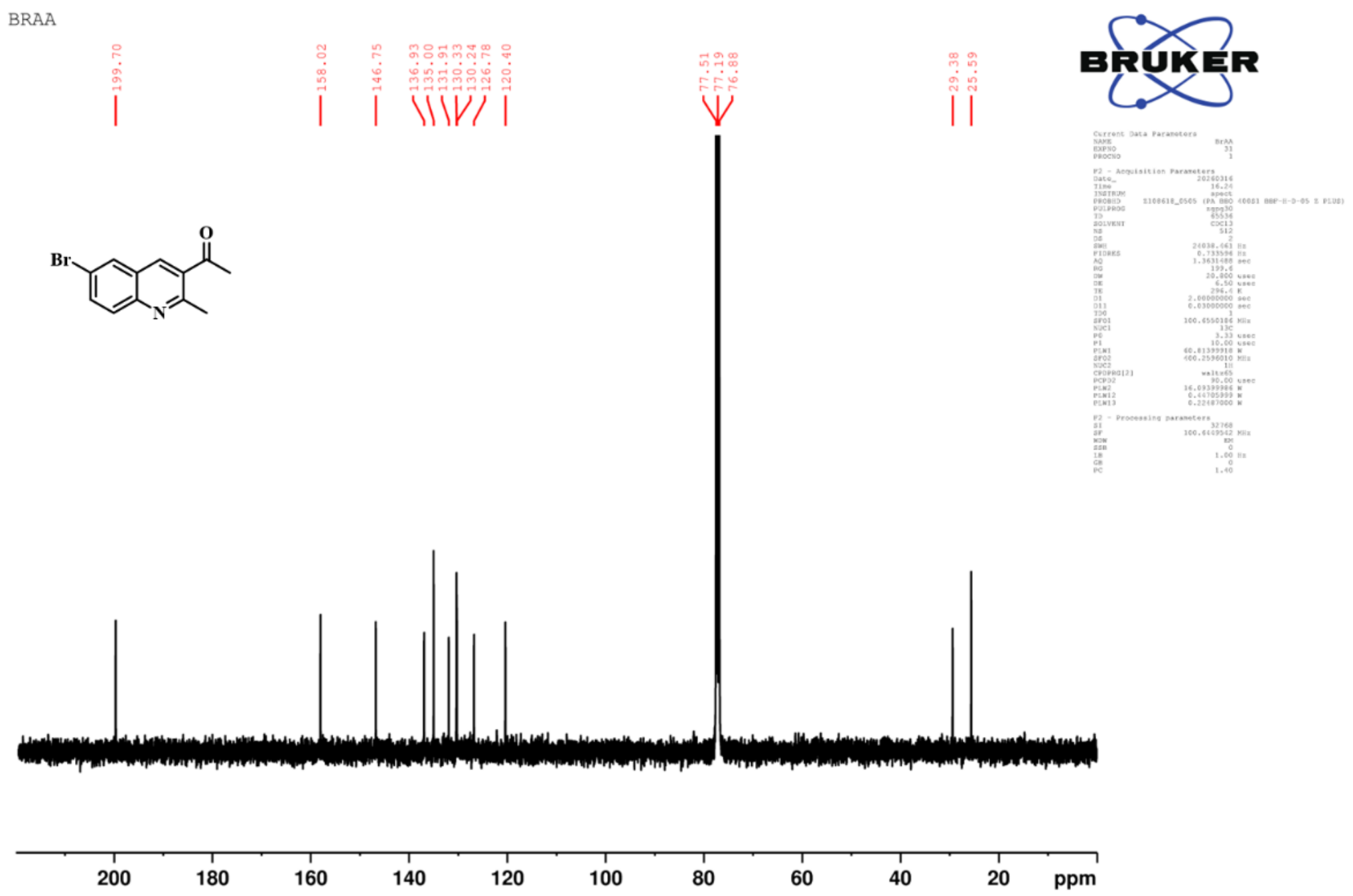

Figure S38. ^{13}C NMR spectrum of compound 3l

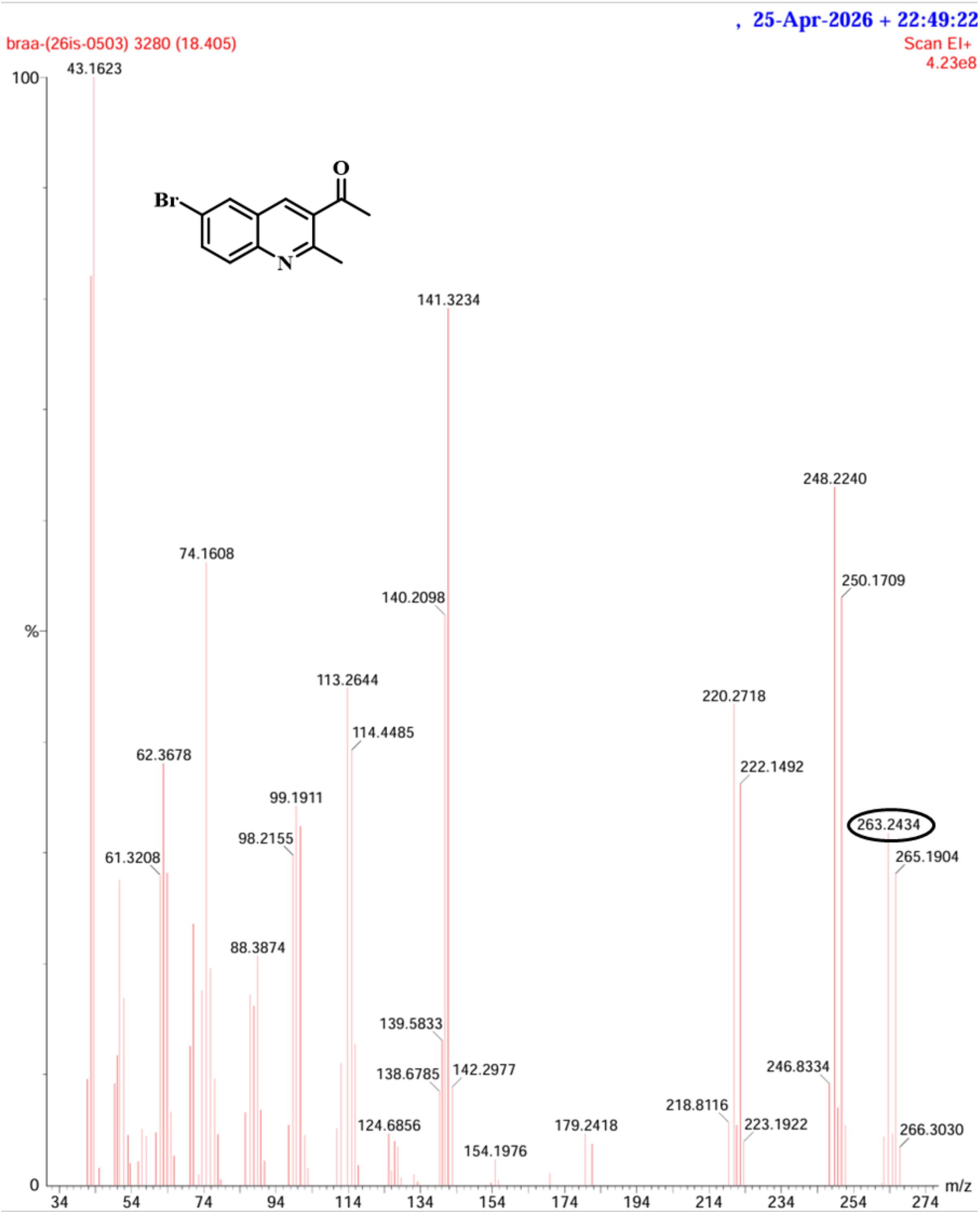

Figure S39. GC-MS spectrum of 3l

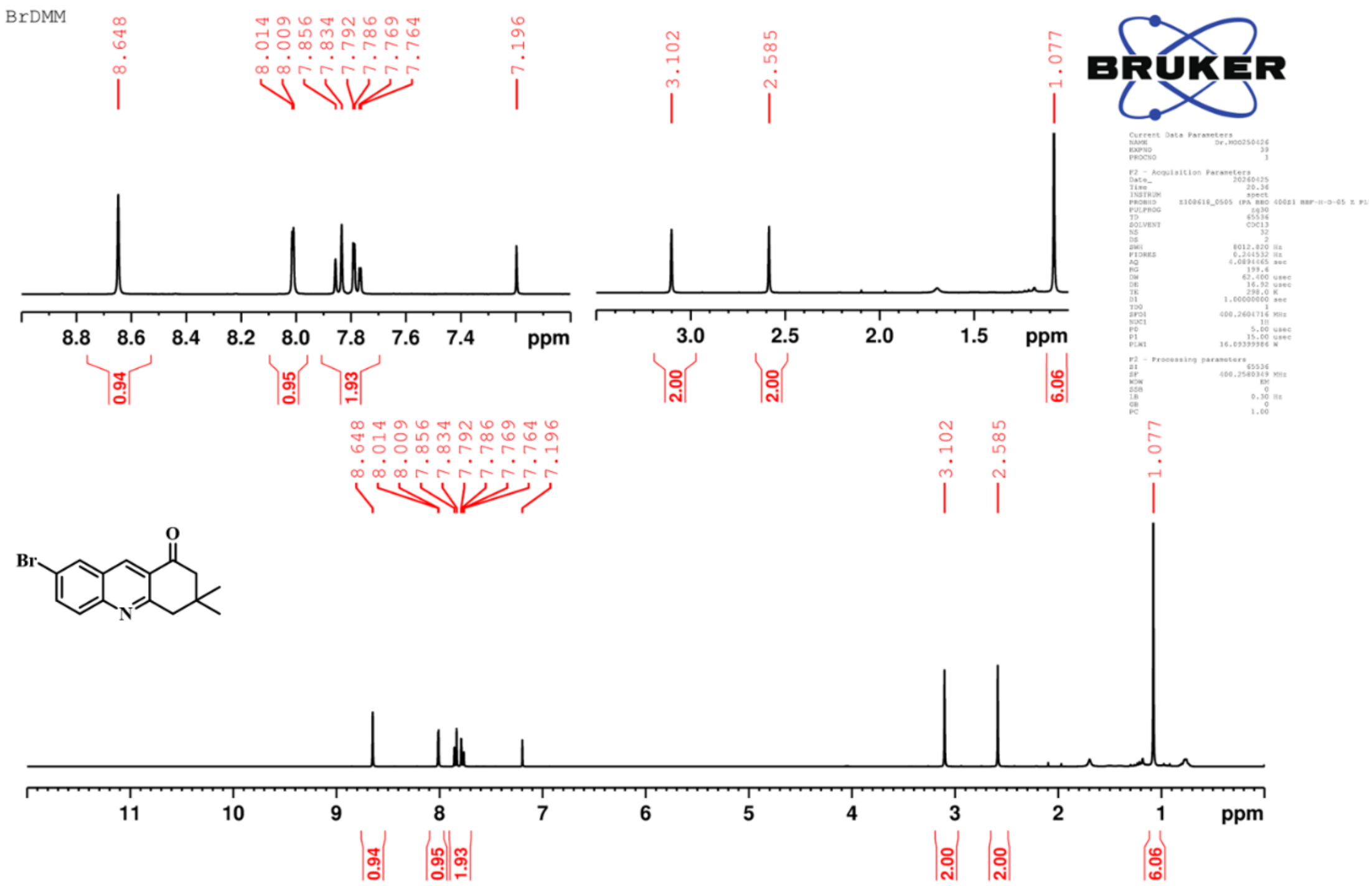

Figure S40. ^{1}H NMR spectrum of compound 3m

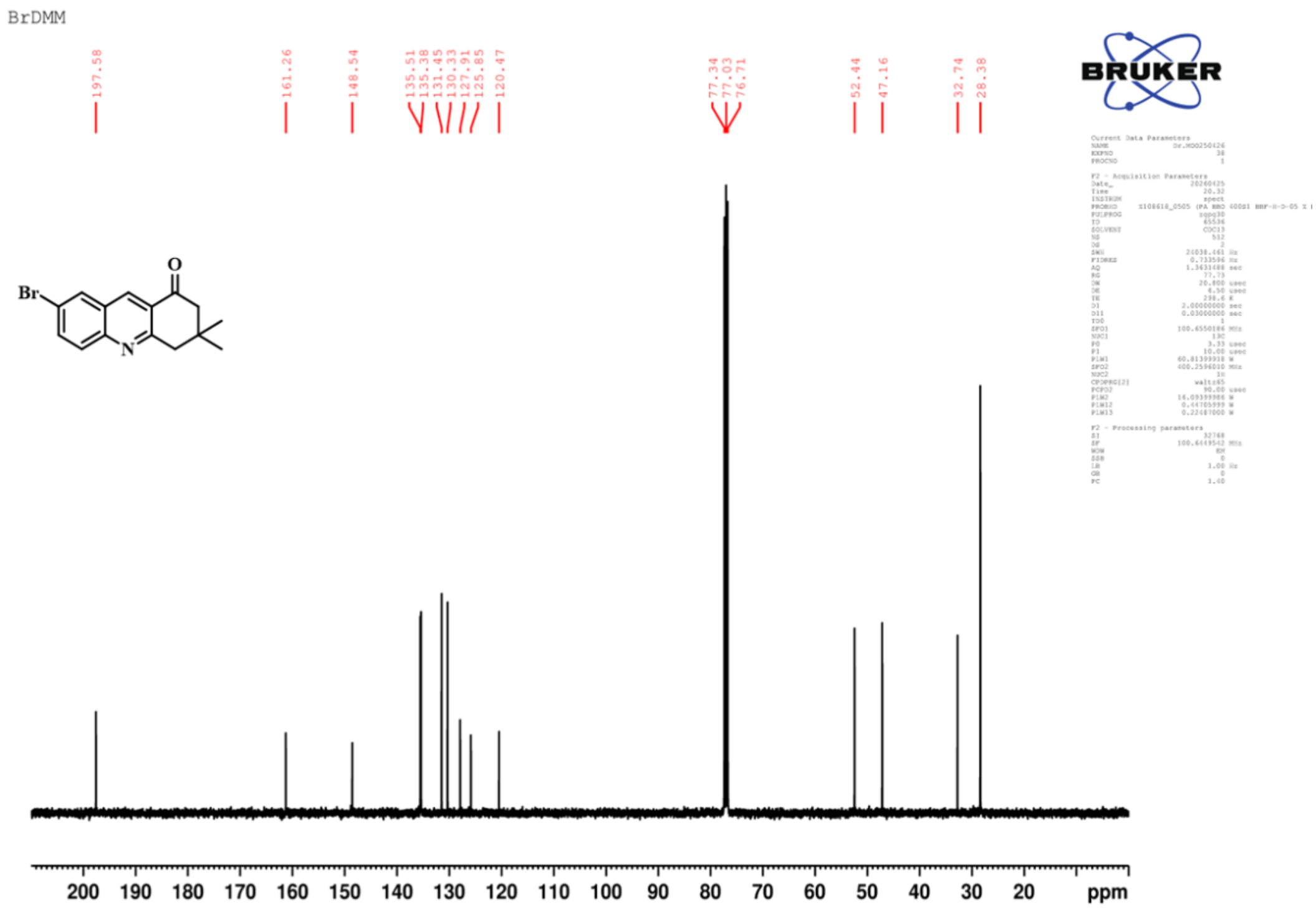

Figure S41. ¹³C NMR spectrum of compound 3m

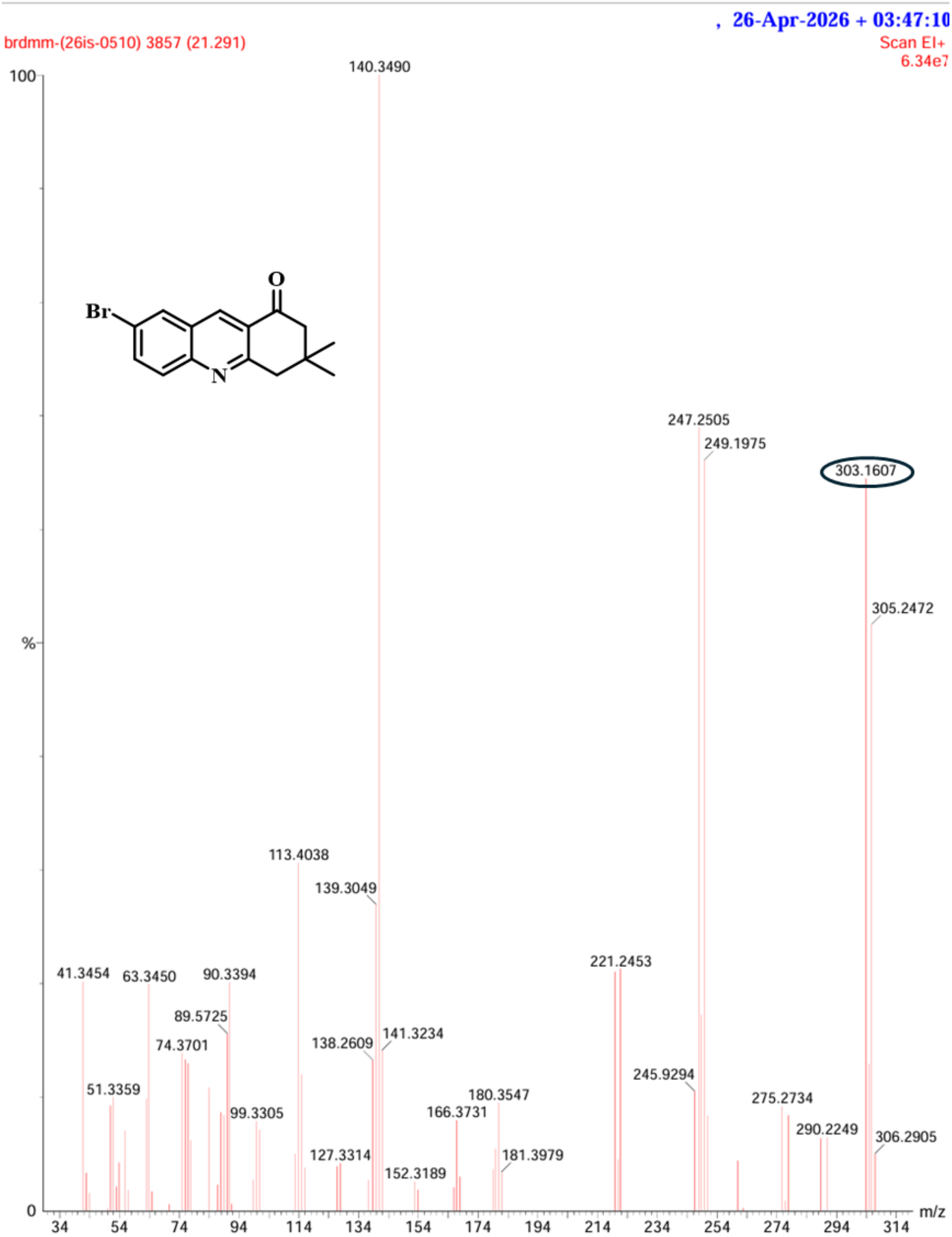

Figure S42. GC-MS spectrum of 3m

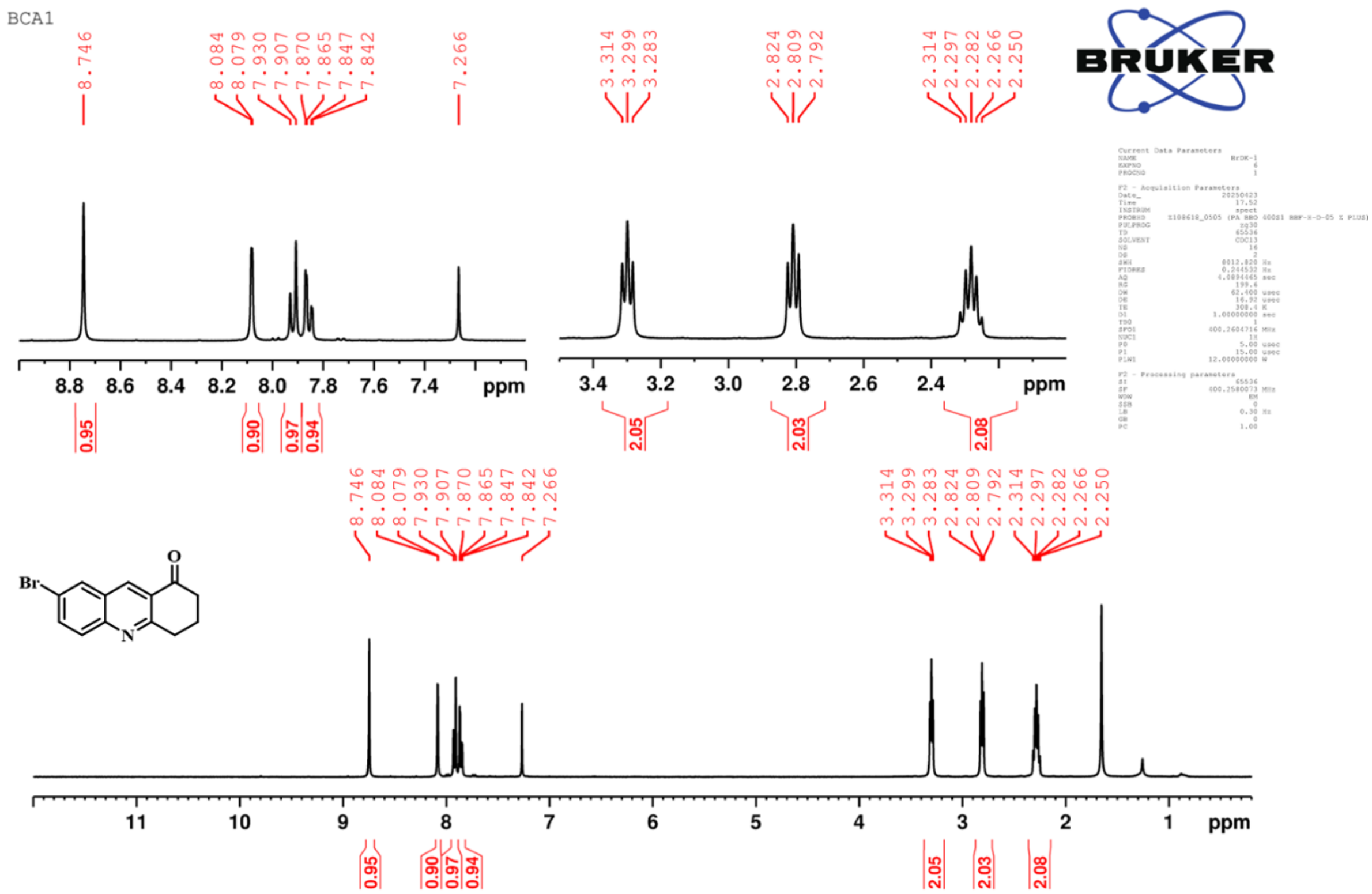

Figure S43. ^{1}H NMR spectrum of compound 3n

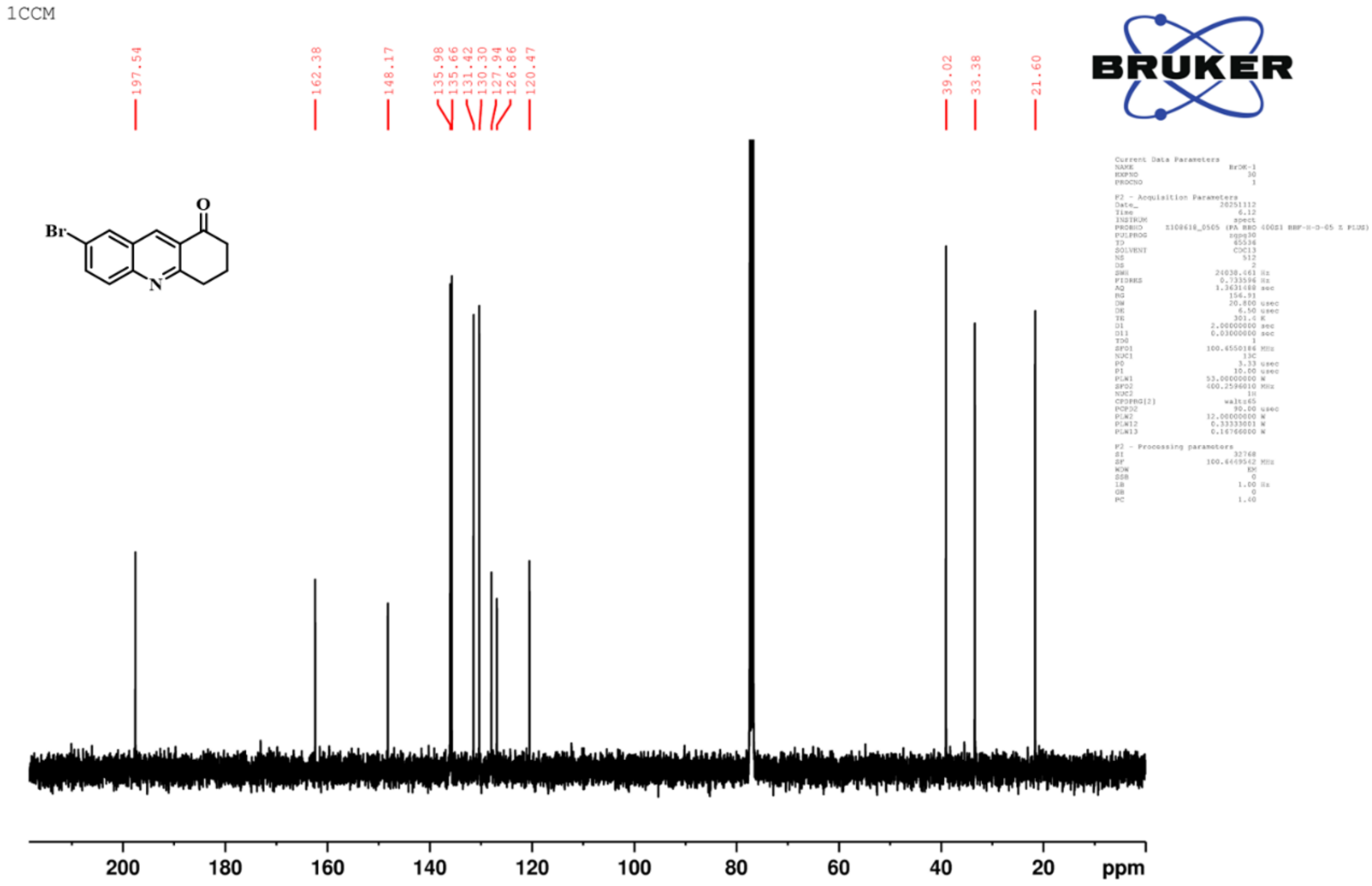

Figure S44. ^{13}C NMR spectrum of compound 3n

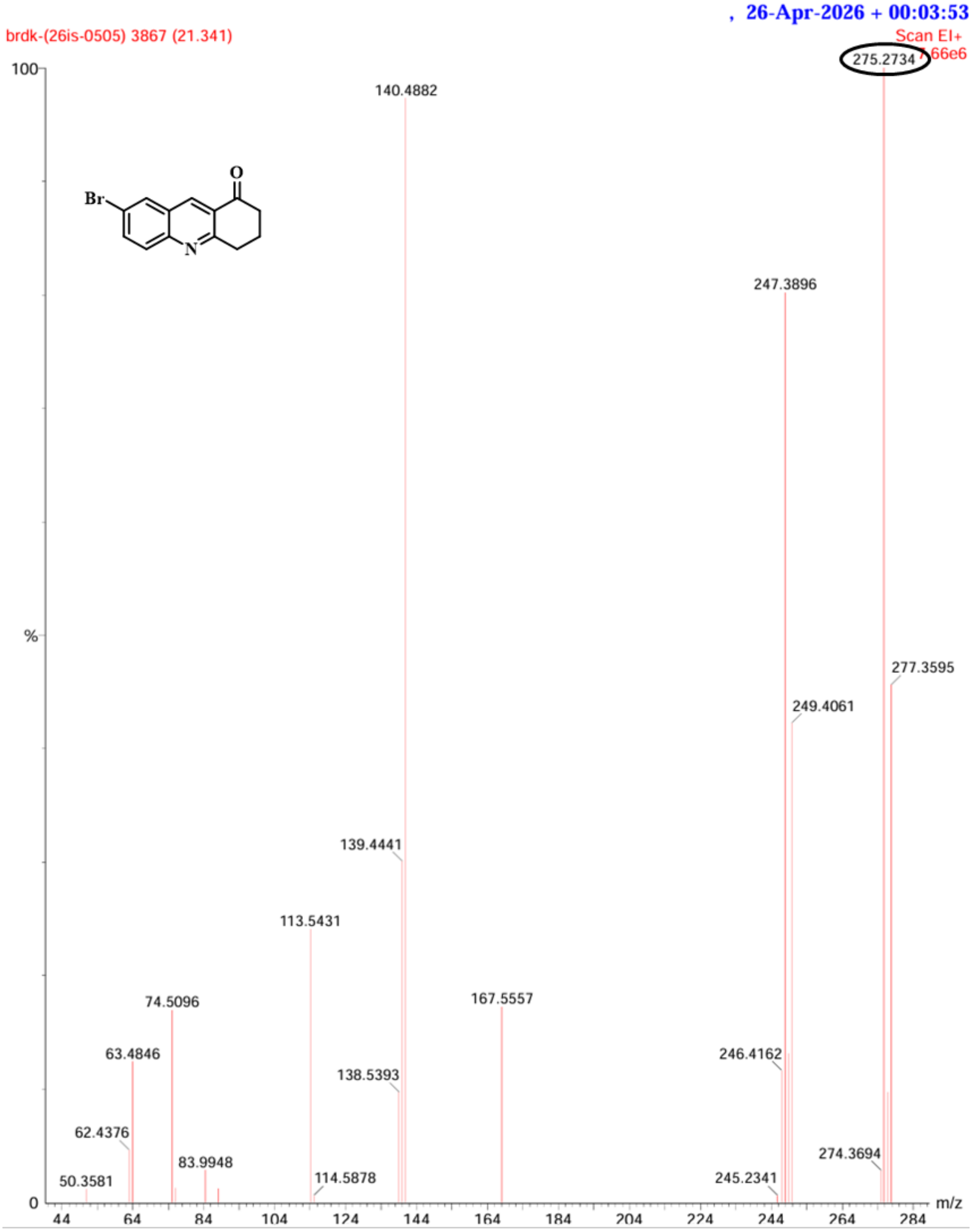

Figure S45. GC-MS spectrum of 3n

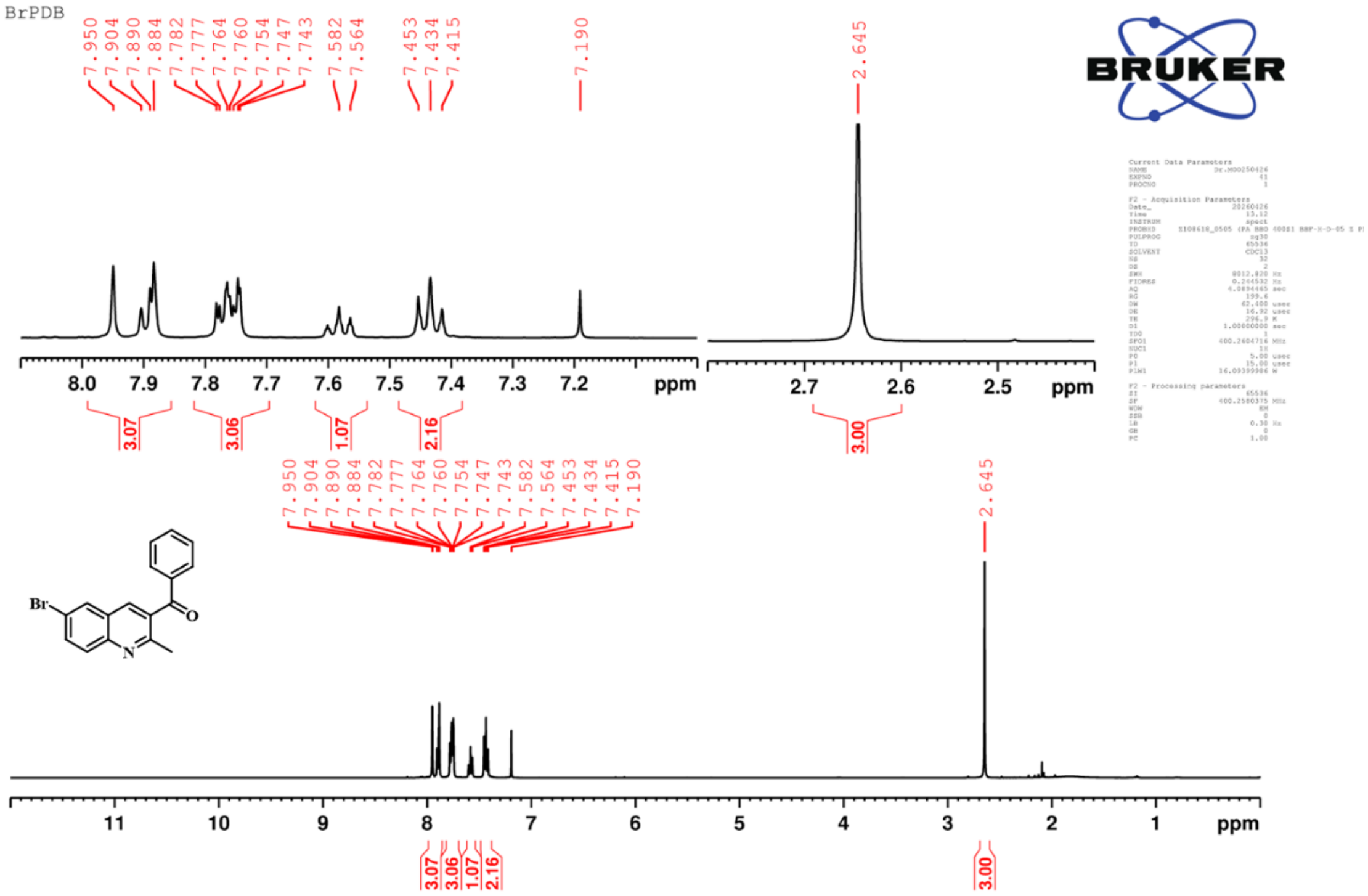

Figure S46. ^{1}H NMR spectrum of compound 30

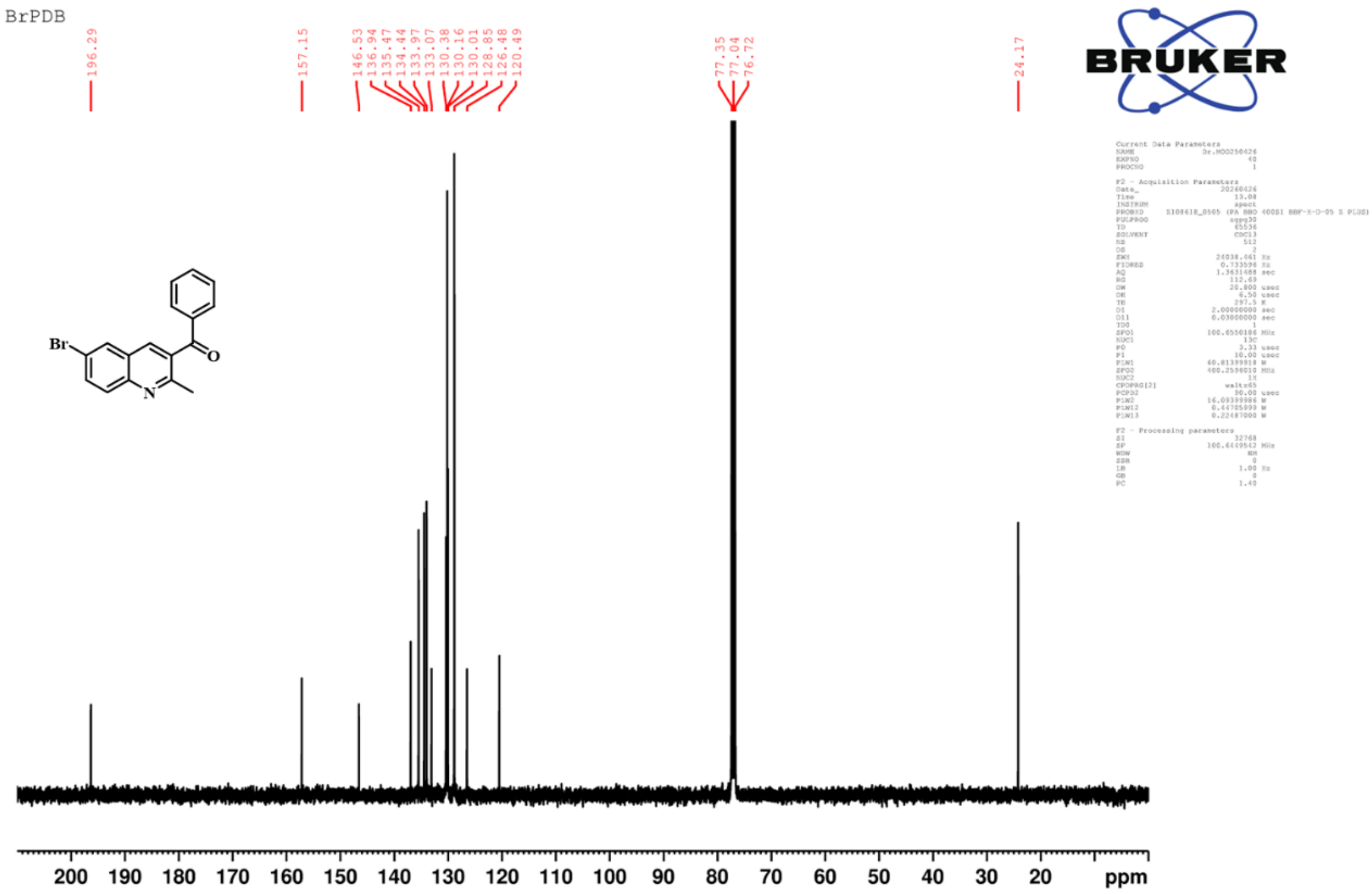

Figure S47. ^{13}C NMR spectrum of compound 30

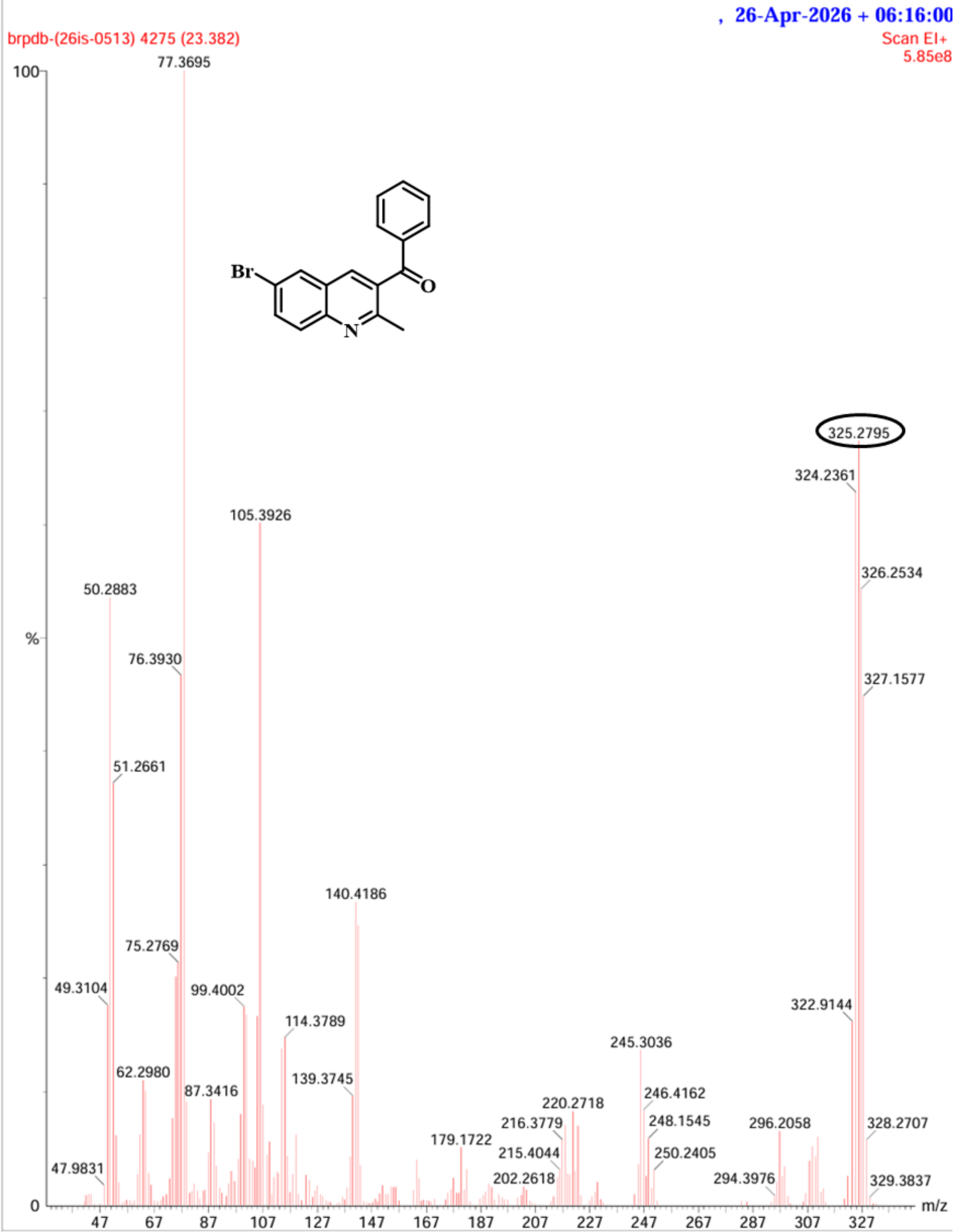

, 26-Apr-2026 + 06:16:00
brpdb-(26is-0513) 4275 (23.382)
Scan EI+
5.85e8
100
%
0
77.3695
325.2795
324.2361
105.3926
50.2883
326.2534
76.3930
327.1577
51.2661
140.4186
75.2769
49.3104
99.4002
114.3789
322.9144
245.3036
62.2980
87.3416
139.3745
246.4162
220.2718
216.3779
248.1545
296.2058
328.2707
179.1722
215.4044
250.2405
47.9831
202.2618
294.3976
329.3837
47
67
87
107
127
147
167
187
207
227
247
267
287
307
327
m/z
Br
N
O

Figure S48. GC-MS spectrum of 3o

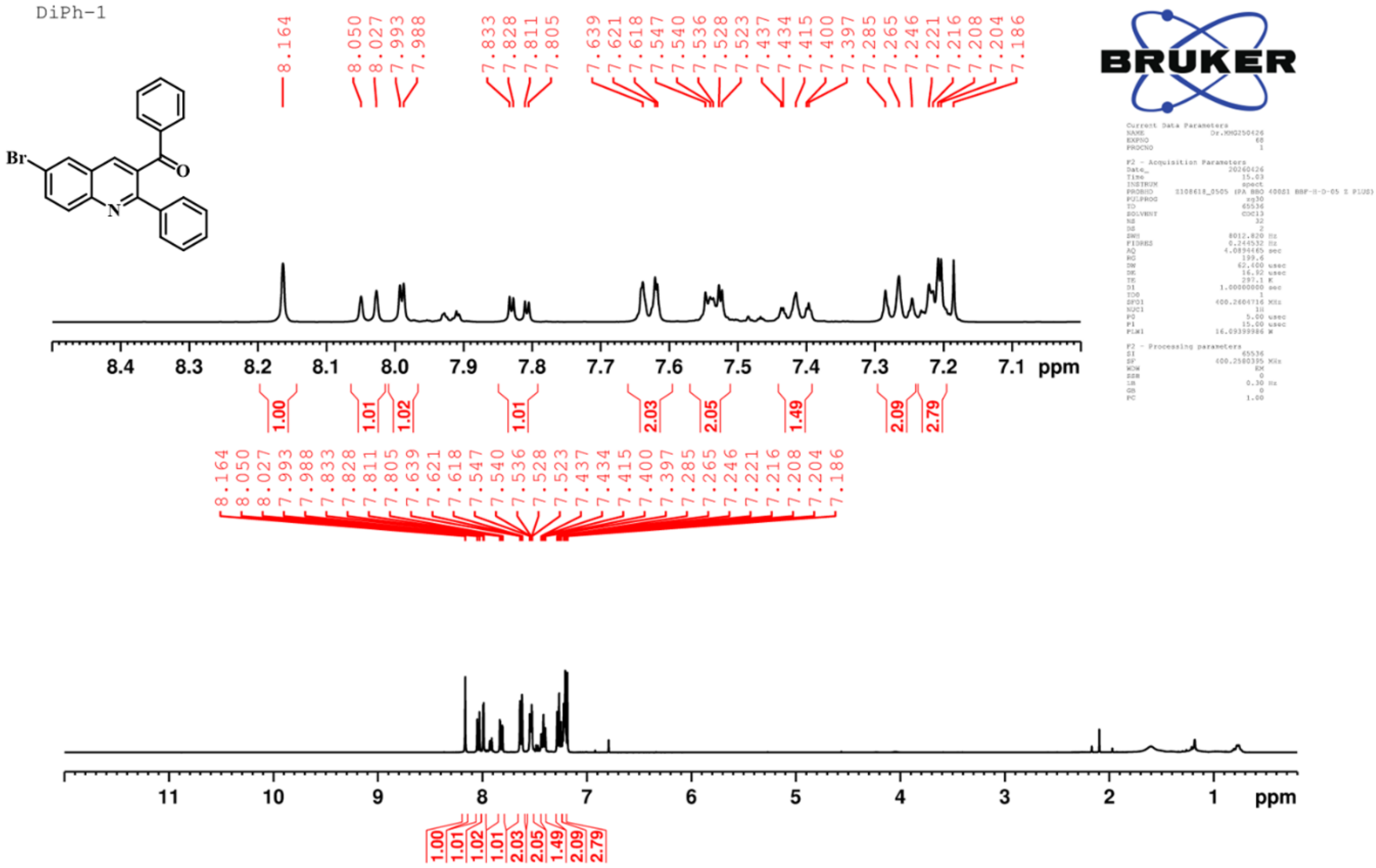

S49. ^{1}H NMR spectrum of compound 3p

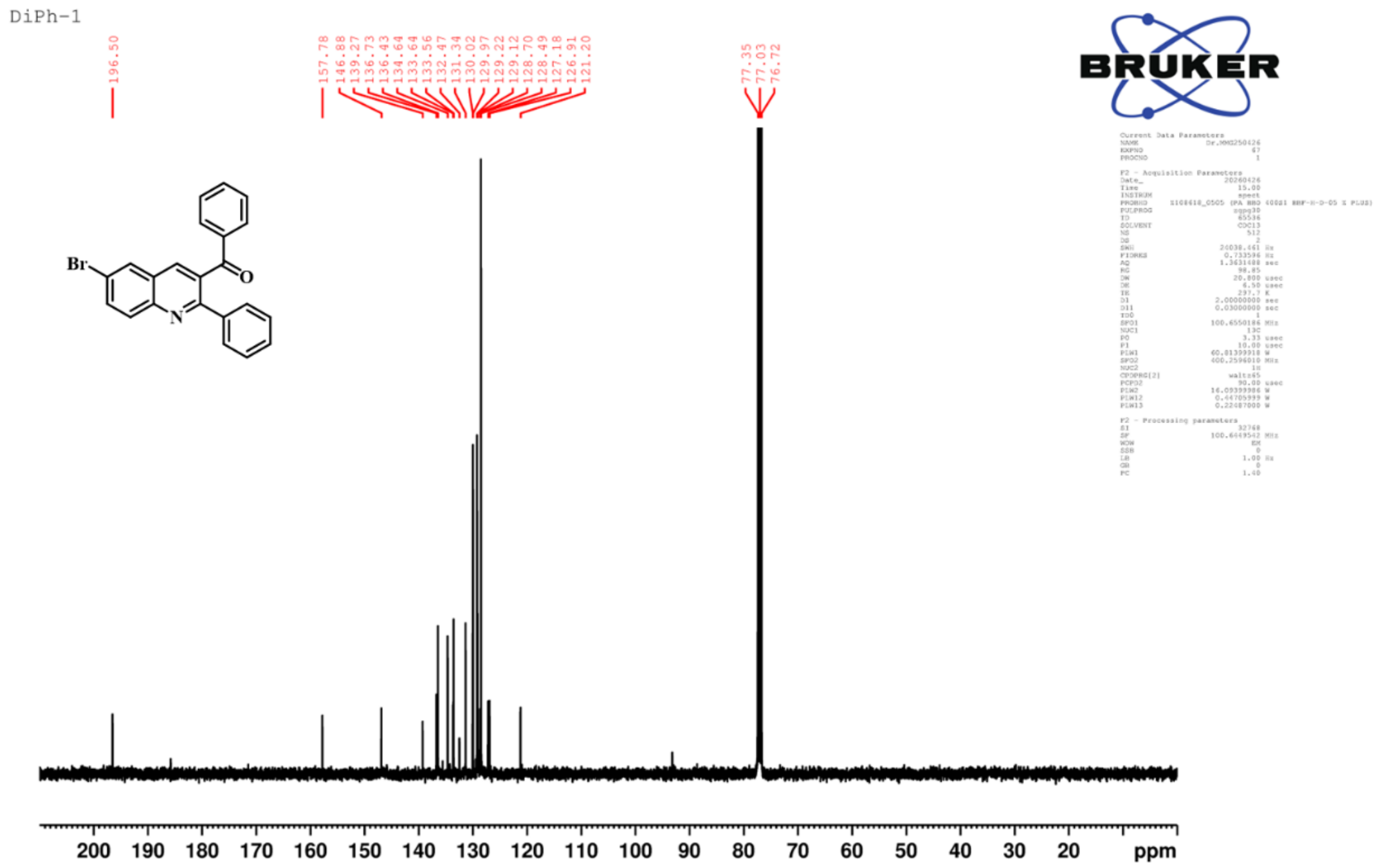

Figure S50. ^{13}C NMR spectrum of compound 3p

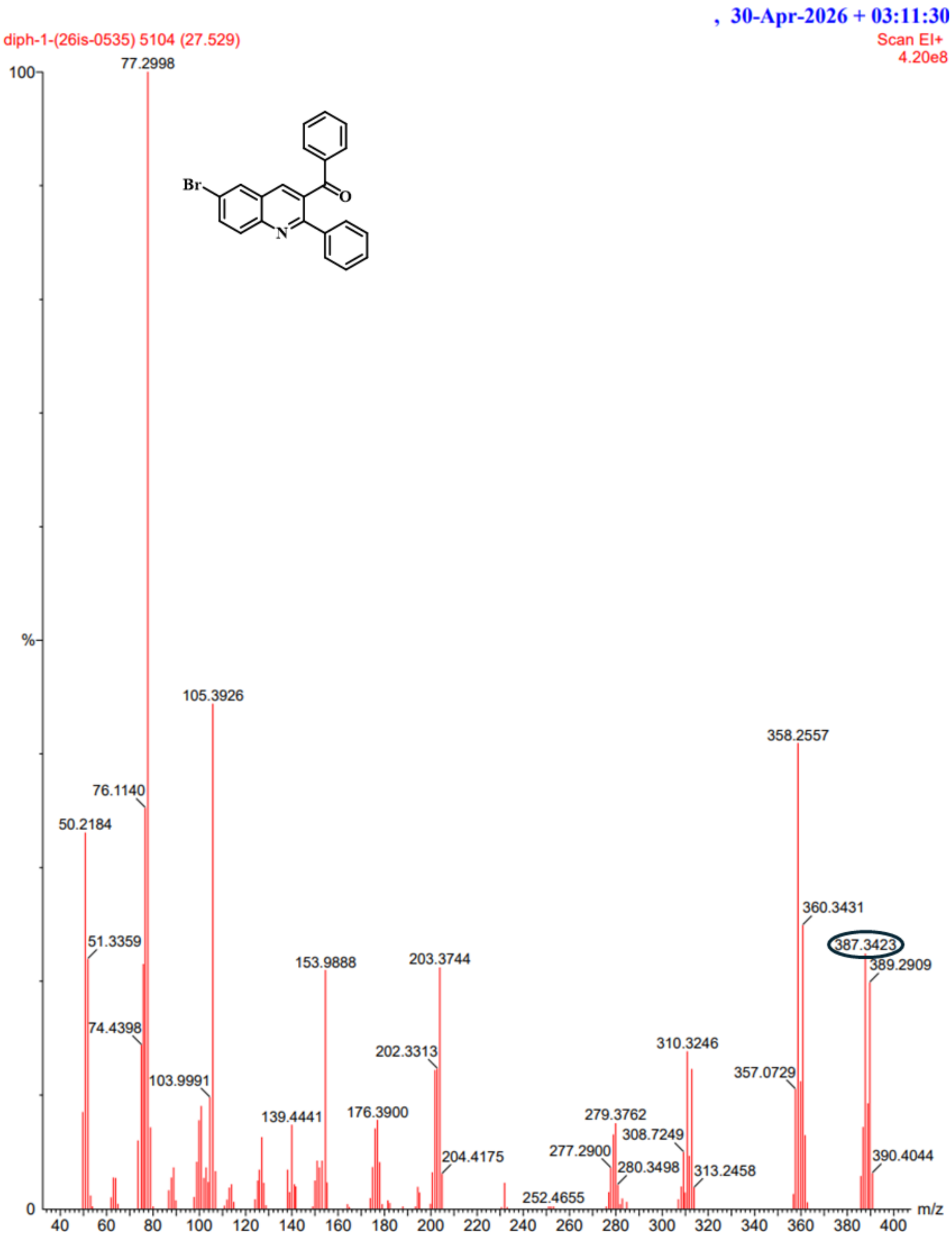

Figure S51. GC-MS spectrum of 3p

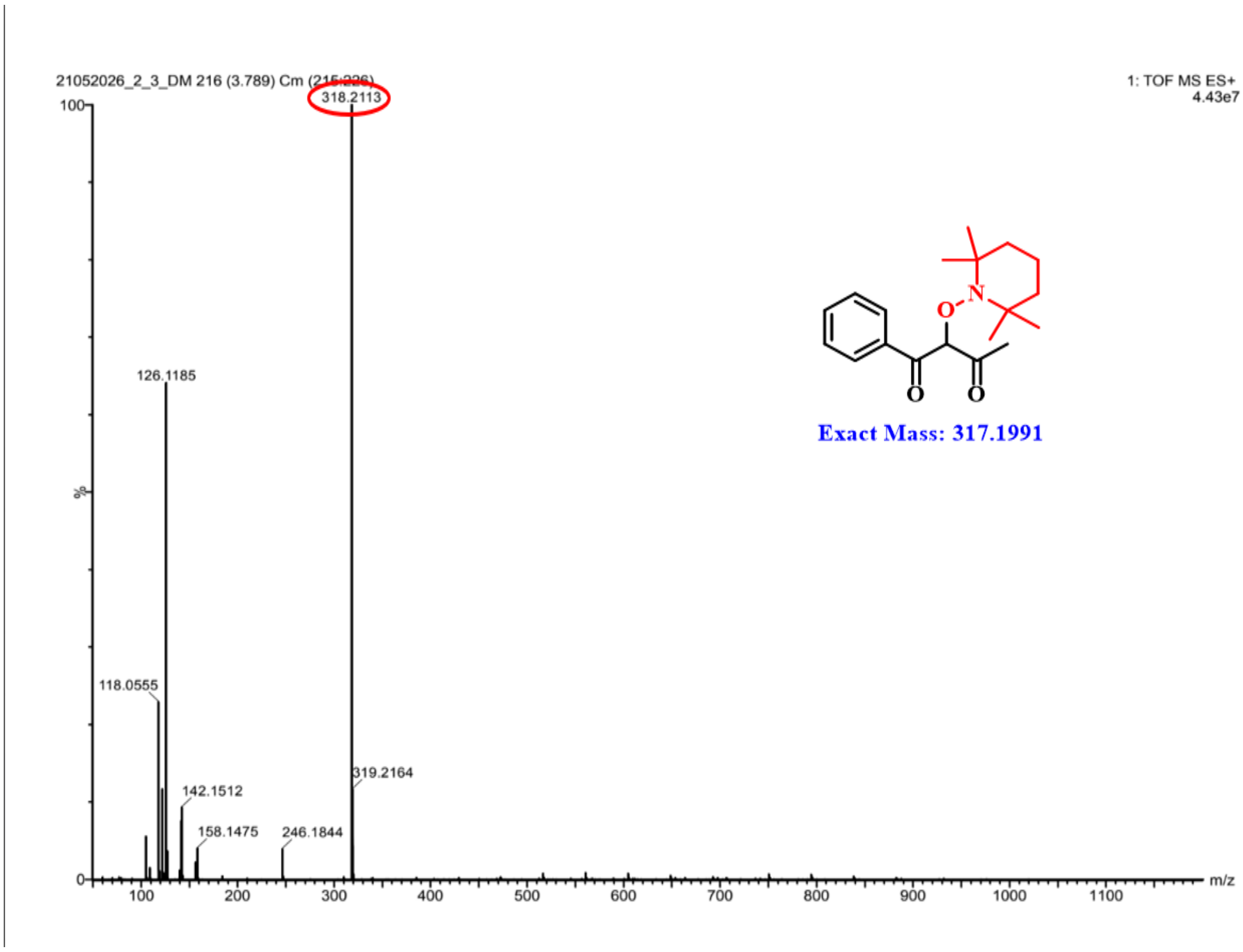

Figure S52. HRMS spectrum of TEMPO adduct
